# Orbitofrontal signals for two-component choice options comply with indifference curves of Revealed Preference Theory

**DOI:** 10.1038/s41467-019-12792-4

**Published:** 2019-10-25

**Authors:** Alexandre Pastor-Bernier, Arkadiusz Stasiak, Wolfram Schultz

**Affiliations:** Department of Physiology, Development and Neuroscience, University of Cambridge, Cambridge, CB2 3DY UK

**Keywords:** Neuroscience, Cognitive neuroscience, Computational neuroscience, Reward

## Abstract

Economic choice options contain multiple components and constitute vectorial bundles. The question arises how they are represented by single-dimensional, scalar neuronal signals that are suitable for economic decision-making. Revealed Preference Theory provides formalisms for establishing preference relations between such bundles, including convenient graphic indifference curves. During stochastic choice between bundles with the same two juice components, we identified neuronal signals for vectorial, multi-component bundles in the orbitofrontal cortex of monkeys. A scalar signal integrated the values from all bundle components in the structured manner of the Theory; it followed the behavioral indifference curves within their confidence limits, was indistinguishable between differently composed but equally revealed preferred bundles, predicted bundle choice and complied with an optimality axiom. Further, distinct signals in other neurons coded the option components separately but followed indifference curves as a population. These data demonstrate how scalar signals represent vectorial, multi-component choice options.

## Abstract (web summary)

Recording from monkey orbitofrontal cortex, the authors used composite reward bundles and found individual neuron and population responses that were suitable for economic choice. The responses followed behavioral indifference curves and predicted behavioral choices consistent with formalisms of Revealed Preference Theory.

## Introduction

Each choice option, irrespective of being a biological reward or an economic good, is composed of multiple components (also called attributes, dimensions, or aspects), and thus constitutes a bundle. Subjective preferences concern all components of a bundle and are revealed by observable choice (whereas unobserved preferences are bound to be capricious and unreliable). The bundle components may be integral parts of a reward or good, like the strawberries and icing of a cake, or consist of separable items, like the steak and vegetable of a meal, or are more abstract, such as quantity and probability of goods^1^ or quality and price^2^. The multi-component character allows a trade-off between the components; when desiring a cake without spending more money (or eating too much), I may give up some strawberries to get more icing. Thus, my preference relation concerns options with multiple components and transcends objective, physical characteristics. Indeed, recognition of the multi-component nature of choice options, rather than considering only single components, helps to explain human choices in health and disease^3–6^.

Stated formally, multi-component choice options constitute vectors. By contrast, neuronal signals are scalar; they can only go up or down in a single dimension. Then, how can a scalar neuronal signal code vectorial choice options? Revealed Preference Theory offers a solution^7^. Preference relations between choice options vary only along one dimension: even with multidimensional options, I prefer option **x** to **y**, or option **y** to **x**, or I am indifferent between the options (completeness axiom^8^). Stochastic preferences revealed by the probability of repeated choices are scalar, as are the subjective values (utilities) of the choice options. As neuronal data analysis requires repeated trials, one can use the theory to capture the emergence of scalar preference relations and utility from vectorial bundles: the trade-off at choice indifference between two bundles with different composition indicates that a reduction in one bundle component compensates for an increase in the other component. Thus, bundle vectors with different composition can be equally revealed preferred, and have the same utility. The trade-off within choice options becomes part of a graphic formalism: equally revealed preferred, but differently composed bundles are positioned on a single indifference curve (IC); and bundles on higher ICs are revealed preferred to bundles on lower ICs. This direct and intuitive concept constitutes the basis for Revealed Preference Theory^9–12^ and its extension to stochastic preferences^13^.

We used the formalisms of Revealed Preference Theory to investigate how scalar neuronal signals in the orbitofrontal cortex (OFC), one of the brain’s major reward structures^14–16^, code stochastic preferences among vectorial bundles of incommensurable bundle components. Previous studies varied single rewards to estimate common-currency scales at choice indifference (equal preference) between rewards of different type, probability, delay, risk, or workload^16–24^. Neurons in OFC integrate or code these different reward aspects separately^16,21,23–26^. However, none of these studies used different quantities of the same two rewards in both choice options that allow implementing the formalism of Revealed Preference Theory. Such a design facilitates the investigation of preference relations between bundles while varying their composition, including the graded, fractional, and continuous trade-off between components within equally preferred bundles. In monkeys, these choices satisfy the two basic axioms for rational preference relations, completeness (either preference or indifference) and transitivity (choice consistency)^8,27^. We now use the graphic formalism of Revealed Preference Theory to discover scalar neuronal signals in OFC for vectorial, multi-component choice options; these signals follow ICs, satisfy out-of-sample tests, predict choice, and respect Arrow’s Weak Axiom of Revealed Preference (WARP) defining optimal choice.

## Results

### Design

With exhaustive choice sets composed of finite numbers of mutually exclusive multi-component options, revealed preferences do not depend on one bundle component alone but their combination into a bundle. The preferences are revealed by measurable choice: bundles that are chosen with equal probability are considered to be equally revealed preferred and to have same utility; a bundle that is chosen with higher probability than any other bundle in that option set is considered to be revealed preferred to that bundle and inferred to have higher utility than that bundle. These notions can be tested by changing the bundle composition; increasing the quantity of at least one bundle component would make that bundle revealed preferred to the original bundle (assuming a positive monotonic value function; more is better); by contrast, two bundles are equally revealed preferred to each other if a higher quantity in one component compensates for less of the other component. The trial repetition required for neuronal data analysis introduced the notion of choice stochasticity, in contrast to single-shot trials typical for human experimental economics. Our design is compatible with basic assumptions of discrete choice theory, Revealed Preference Theory and its stochastic version (for details, see the Methods section: Implementation of basic concepts)^7,13,28,29^.

**Fig. 1:**
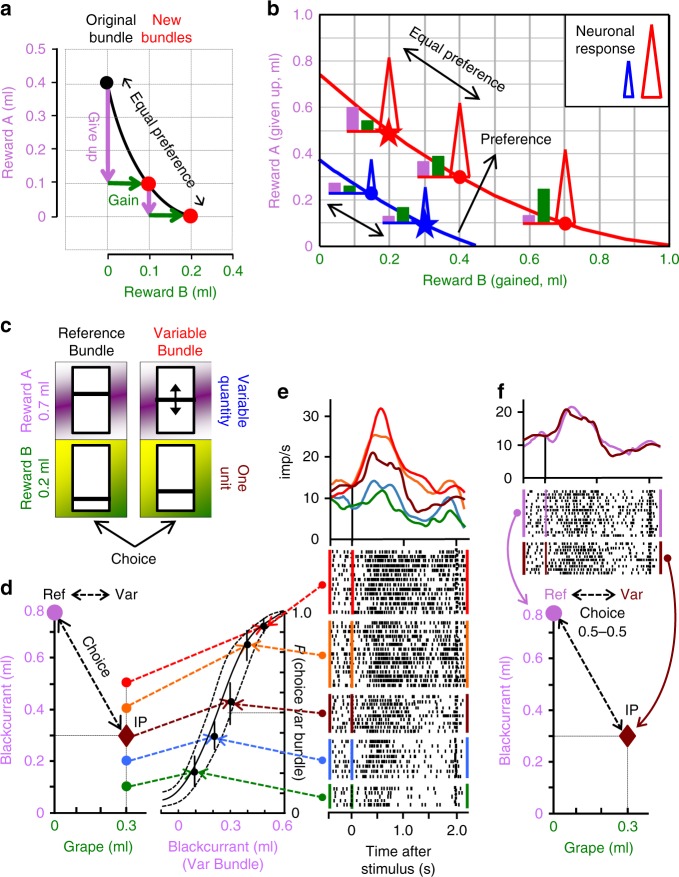
Design and defining the data. **a** Equal revealed preference from varying bundle composition (inspired by a textbook scheme^12^). The original (black) and new bundles (red) are equally revealed preferred (black curve) when a quantity of reward A is traded in (violet; initially 0.3 ml, then 0.1 ml) for one unit of reward B (green; 0.1 ml). **b** Neuronal revealed preference scheme: scalar neuronal signals (triangles) follow revealed preference for vectorial bundles across curves (blue, red), even with one smaller bundle component (stars); neuronal responses are similar along equal-preference curves despite varying bundle composition (pink, green). **c** Bundle stimuli. Each bundle contained the same two rewards (violet, green) with independently set quantity, as indicated by vertical bar position within each rectangle (higher is more). The Reference Bundle contained two preset reward quantities. The Variable Bundle contained a specific quantity of one reward and an experimentally varied quantity of the other reward. **d** Behavioral trade-off among bundle rewards. Left: choice between Reference Bundle (0.8 ml blackcurrant juice, 0 ml grape juice) and Variable Bundle (0.1–0.5 ml varying blackcurrant juice, 0.3 ml grape juice). Oblique double-arrow line connects two equally revealed preferred bundles (IP, indifference point). Right: psychophysical assessment: choice probability for Variable Bundle increases inversely with blackcurrant juice being given up (Weibull fit ± 95% confidence interval). At IP, 0.5 ml of blackcurrant juice were traded in for 0.3 ml of grape juice. Also, the animal preferred bundles with less blackcurrant to the Reference Bundle with more blackcurrant when grape juice compensated for the reduction (red, orange). **e** Neuronal bundle stimulus response increases with choice probability of Variable Bundle over Reference Bundle (from green to red) (*P* < 0.005; *t* test in linear regression; Eq. 5). Lower trial numbers at the bottom reflect infrequent choice of non-preferred bundles. Bundles alternated pseudorandomly; peri-stimulus time histograms (PSTH, top) and impulse rasters were post hoc ordered. **f** Similar neuronal responses to equally revealed preferred bundles at trade-off (*P* = 0.5 each option; double-arrow lines)

A two-component bundle is plotted at the intersection of the *x*-coordinate (reward B) and *y*-coordinate (reward A) (Fig. 1a). The *x*–*y* positions of equally revealed preferred bundles constitute choice indifference points (IP). The two red dots in Fig. 1a are IPs relative to the black dot, and relative to each other. Several IPs of equally revealed preferred bundles align as an indifference curve (IC), and are inferred to have equal utility. All bundles on higher ICs (farther from origin) are revealed preferred to, and have higher utility than, all bundles on lower ICs (Fig. 1b). The complete set of ICs summarizes the monkey’s preferences.

Scalar neuronal signals can represent vectorial, multi-component choice options in several ways. An integrated form would follow the graphic formalisms of Revealed Preference Theory, and its stochastic version^13,28^, that capture single-dimensional preferences of multidimensional bundles: responses should reflect the trade-off within bundles that characterizes the emergence of scalar preferences and utility from vectorial bundles. The neuronal responses should be indistinguishable between equally revealed preferred but differently composed bundles, and thus between all bundles on the same IC (Fig. 1b, along blue and red ICs). By contrast, the signal should change monotonically across ICs (from blue to red), despite varying bundle composition and even when one component is lower in the preferred bundle than its alternative (stars; partial physical non-dominance). This form requires neurons that are sensitive to multiple rewards, which has been shown for OFC and other brain structures^16,20,29,30^. Even if these revealed preference responses follow the rank order of choice probabilities (ordinal relation), they would not necessarily correlate in a numeric (cardinal) way with choice probability. The second possibility would be distinct neuronal signals for each component (single-reward coding), and then aggregation of the distinct signals into a common scalar signal that represents the vectorial choice option. Our design allows testing both versions.

### Behavior

We presented rhesus monkeys with a symmetric option set containing a preset Reference Bundle and a simultaneously presented Variable Bundle that contained the same two, independently variable, liquid rewards (Fig. 1c) (the axiomatic test described below employed three options). There was no unambiguous distinction between the two bundles; therefore, we could not assess neuronal coding of object value, which reflects the value of a distinct choice object. Each bundle was presented at equal reaching distance to the animal and was equally affordable. The task contained four epochs following a Pretrial control epoch: Bundle stimulus, Go signal, Choice, and Reward of the chosen bundle (first reward A, then reward B after 0.5 s) (Supplementary Fig. 1; see the Methods section: Visual stimuli and reward bundles, and Behavioral task).

To estimate an IP, we set both rewards of the Reference Bundle, and one reward of the Variable Bundle, to specific quantities and varied the other Variable Bundle reward psychophysically (Fig. 1d). The animal’s choice probability followed the single-variable reward of the Variable bundle. We considered a bundle as revealed preferred when chosen with *P* > 0.5 over the alternative bundle; as conceptualized and typically found with stochastic choices^13,28,29^, the animals chose the alternative bundle on some trials. Two bundles were considered as equally revealed preferred when each was chosen with *P* = 0.5, as judged from fit by a Weibull function. At the IP in Fig. 1d, the animal gave up 0.5 ml of blackcurrant juice (pink) to obtain 0.3 ml of grape juice (green). This trade-off, by which the partial gain in grape juice compensated for the partial loss of blackcurrant juice, explained the equal revealed preference documented by the IP, despite varying composition of the Variable Bundle. Trading-off other quantities of blackcurrant juice for 0.3 ml of grape juice led to consistent, monotonically changing choice probabilities (Fig. 1d, right). With limited loss of blackcurrant juice (red and orange), the animal revealed preferred the Variable Bundle even when it contained less blackcurrant juice than the Reference Bundle (pink) (partial physical non-dominance).

To obtain an IC, we set the Reference Bundle to specific quantities and fitted hyperbolic functions to the IPs of all equally revealed preferred bundles (Eq. 1; see the Methods section: Estimation of behavioral ICs). We obtained 30 ICs from 545 IPs of bundles containing blackcurrant juice and either grape juice, water, apple squash, or mango juice, some of them with added monosodium glutamate (MSG) or inositol monophosphate (IMP) (Supplementary Fig. 2).

Logistic regressions suggested that choice of the Variable Bundle was based on reward difference rather than spatial choice, bundle stimulus position, previous bundle or spatial choice, or consecutive trial number (Supplementary Fig. 3). The animals fixated their eyes on both bundles before the choice and then on the chosen bundle until it disappeared with reward delivery (Supplementary Fig. 4).

To assure stable economic conditions, we abandoned neuronal recordings when satiety was detected by psychophysical choice functions that exceeded the confidence intervals of initial tests and indicated changed currency relations between the two bundle rewards (Supplementary Fig. 5: blue, orange, and red functions outside green zone).

### Neuronal database

We recorded extracellular electrophysiological activity in OFC (Supplementary Fig. 5) from 694 single neurons with 3–7 bundles per IC on 3–5 ICs, testing at least 9 bundles per neuron (average of 15 bundles; total of 618 different bundles). Of the 694 neurons, 441 showed task-related activity during binary choice over zero bundle (*n* *=* 325), between two nonzero bundles (*n* *=* 391, including the 325 neurons), and between three nonzero bundles (*n* *=* 56, including 6 neurons from the binary tests), as defined by significant activity difference between one task epoch and pretrial control (*P* < 0.01; paired Wilcoxon test and one-factor ANOVA). We excluded trials from 163 of the 694 neurons after satiety had set in toward the end of daily testing (23.5%), while enough trials remained for data analysis (>15 trials/bundle).

Neuronal revealed preference responses were defined according to the ICs of Revealed Preference Theory: insignificant variation between IP bundles on single ICs, but monotonic change across bundles on increasing ICs (Fig. 1b) (we use “revealed preference responses” to refer to Revealed Preference Theory, although preferences can be revealed in any observable choice). We subjected the 441 task-related neurons to three tests: (1) double linear regression assessing change with both bundle rewards across ICs with minimal assumptions (Eq. (4); *P* < 0.05; *t* test), which captured the scalar neuronal activity representing the vectorial bundle: *y* = f (reward A, reward B); (2) Spearman rank-correlation confirming change monotonicity across ICs (*P* < 0.05); (3) two-factor ANOVA demonstrating change across ICs (*P* < 0.05), but not within ICs (*P* > 0.05) (significance in first factor indicates sensitivity that renders insignificance in second factor indicative of response similarity). A neuronal revealed preference response had to satisfy all three tests.

### Neuronal responses during IP estimation

Bundle stimulus responses in single OFC neurons increased monotonically with increasing choice probability (Fig. 1e). The responses were stronger to the revealed preferred bundle, even with less blackcurrant juice in the preferred than in the alternative bundle (red, orange) (partial physical non-dominance, stars in Fig. 1b). Responses were similar to two equally revealed preferred bundles when 0.5 ml of blackcurrant juice was traded-off for 0.3 ml of grape juice (Fig. 1f, pink vs. Bordeaux). Thus, the scalar neuronal changes reflected the variations in the composition of the vectorial bundles.

### Revealed preference coding during choice over zero-reward bundle

**Fig. 2:**
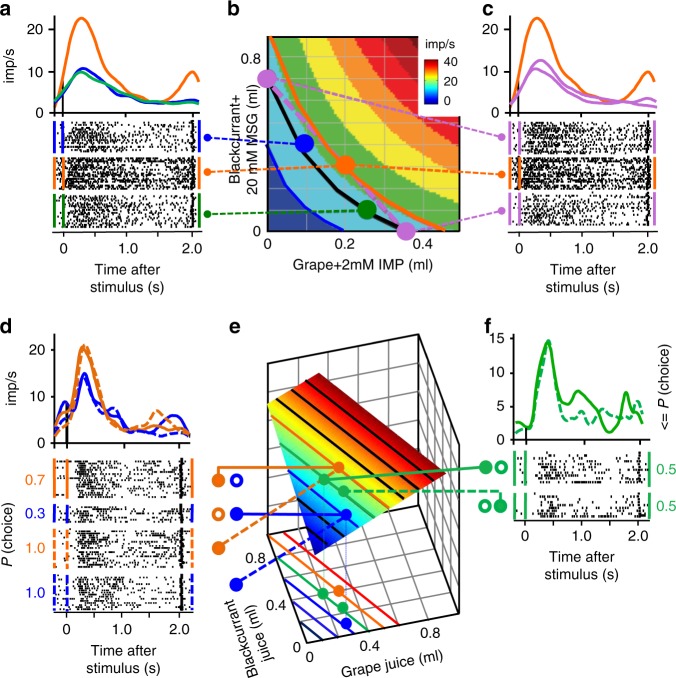
Revealed preference coding in single orbitofrontal neurons: scalar neuronal signals representing vectorial reward bundles. **a** Similar stimulus response to equally preferred bundles on same indifference curve (IC) (blue, green), and stronger response to revealed preferred bundle on higher IC (orange). The response to the revealed preferred orange bundle was stronger, despite one of its rewards being smaller than in the alternative blue and green bundles. Choice over zero-reward bundle. **b** Bundle positions (colored dots) on hyperbolically fitted behavioral ICs (pink, blue, orange) tested in **a** and **c**. Bundles on orange IC were revealed preferred to bundles on black IC. Traverse dotted lines connect bundle positions to neuronal rasters in **a** and **c**. Colored bands show neuronal stimulus responses for bundles whose components are specified by their *x*–*y* coordinates (blue to green to red) (modeled from Eq. (7b); imp/s: impulses/s). MSG monosodium glutamate, IMP inositol monophosphate. **c** Response in same neuron follows IC convexity rather than simple liquid quantity exchange: responses were lower for bundles positioned at ends of dotted pink line in **b**, compared with center bundle on higher IC (orange dot). The full test with this neuron is shown in Supplementary Fig. 7. **d**, **e** Chosen value response during choice between two nonzero bundles. The stimulus response was stronger with choice of the revealed preferred bundle (choice probability *P* = 0.7; solid orange), compared with choosing the alternative bundle (*P* = 0.3; solid blue); the respective responses were similar when each bundle was chosen separately with *P* = 1.0 over a zero-reward bundle (dotted orange and blue), demonstrating independence from alternative bundle and suggesting absolute chosen value coding. **e** shows behavioral ICs (lines) and modeled neuronal responses (bands). More responses of this neuron during choice over zero bundle are shown in Supplementary Fig. 8. **f** Trade-off in chosen value response: similar response to equally revealed preferred bundles on same behavioral IC, despite different bundle composition (*P* = 0.5 choice each, solid vs. dotted lines). Dots between **d**–**f**: filled: chosen nonzero bundle, open: non-chosen nonzero bundle, dot absence: non-chosen zero-reward bundle

The full revealed preference test followed the scheme of Fig. 1b and used bundles at specific IPs on established behavioral ICs. To facilitate data interpretation, we focussed the animal’s choice on a single bundle by presenting an alternative valueless, zero-reward Reference Bundle (see the Methods section: Trial types for neuronal tests). The neuronal responses followed the scheme of Fig. 1b: they were similar with different bundles located on the same IC, even though their reward quantities varied in opposite directions (Fig. 2a, b, gain of 0.15 ml grape–IMP and loss of 0.3 ml blackcurrant–MSG between blue and green bundles); thus, they reflected the characteristic trade-off between bundle components. Furthermore, the neuronal responses were stronger to the revealed preferred bundle (Fig. 2a, b, orange PSTH and IC) and involved both rewards; the preferred bundle (orange) contained less blackcurrant–MSG than the blue bundle, and less grape–IMP than the green bundle (partial physical non-dominance). The increase did not reflect monotonic coding of a single reward: the orange bundle response should be lower than the blue bundle response if coding only blackcurrant–MSG quantity, and lower than the green bundle response if coding only grape–IMP quantity, but neither was the case. Thus, the scalar neuronal signal followed the revealed stochastic preference of vectorial bundles as represented by the ICs despite oppositely varying bundle composition, suggesting that it captured the well-ordered empirical preference relations. The neuronal compliance with equal-preference trade-off and partial physical non-dominance is compatible with the emergence of unidimensional, scalar neuronal responses from multidimensional changes in the vectorial bundle.

A convex IC indicates a change in trade-off between bundle components; the animal trades in more quantity for a gain in the other bundle reward in the IC center compared with the IC periphery. The location on a higher IC suggested that the animal revealed preferred the center bundle to the peripheral bundles, despite all bundles being located on the same straight line (Fig. 2b, orange and pink bundles). Accordingly, neuronal responses were stronger to the center bundle compared with the two peripheral bundles on the lower IC (Fig. 2c, orange vs. pink; complete test in Supplementary Fig. 7). The stronger response in the center than the periphery of the straight line reflected well the nonlinear behavioral trade-off.

A linear IC indicates constant trade-off. As with convex ICs, neuronal bundle responses increased across ICs and were similar for bundles on the same linear IC (Supplementary Fig. 8). Finally, a concave IC indicates trading-in less quantity for a gain in the other bundle reward in the IC center compared with IC periphery. As with linear and convex ICs, neuronal bundle responses were similar along concave ICs and increased across ICs (Supplementary Fig. 9). Other OFC neurons showed inverse coding; their responses decreased with increasing preference across ICs while being similar along ICs (Supplementary Fig. 10). As with convex ICs, the increases or decreases across linear and concave ICs occurred, despite one liquid being lower in the revealed preferred compared with the alternative bundle (Supplementary Fig 8a, b, orange vs. green; 9a, b, red vs. green; 10a, b, green vs. orange). Thus, OFC responses followed closely the convex, linear, and concave ICs that characterized the well-ordered revealed preferences.

In total, during choice over zero-reward bundles, our basic three-test procedure involving Eq. (4) identified revealed preference coding in 263 responses in 139 of 325 task-related neurons (43%) with all five bundle types and in any of the four task epochs (Table 1; Supplementary Table 1; see Methods: Statistical analysis of neuronal revealed preference coding). Besides this conservative analysis, regressions with interaction respecting the nonlinearity of ICs (Eqs (7–9)) yielded higher adjusted R2s in these 139 neurons (*P* < 0.05; Fisher’s test; Supplementary Tables 2, 3), with the gain being strongest with the simplest model (Eq. (7)). Spearman rank-correlation across ICs yielded mean rho = 0.5921 for 98 positive coding neurons and rho = −0.6100 for 41 inverse coding neurons (*P* < 0.05). Polar plots illustrate the responses increases (or decreases with inverse coding) with increasing reward quantity (Supplementary Figs. 11, 12). The extended regression (Eq. (10)) confirmed coding of rewards and choice of the Variable Bundle, but not of previous choice (Supplementary Fig. 13a). By contrast, additional 68 of the 325 task-related neurons (21%) showed significant response changes both across and along ICs (i.e., significance with both factors of the two-factor ANOVA; excluding single-reward responses); in failing to follow the characteristic IC scheme, these neurons did not code revealed preference.

**Table 1:** Revealed preference coding during choice over zero bundle

Bundle type	Tested neurons	Neurons responding	Responses
Blackcurrant, grape	81	22 + 6 = 28	37 + 8 = 45
Blackcurrant, water*	138	39 + 20 = 59	72 + 41 = 113
Blackcurrant, apple	29	12 + 5 = 17	20 + 10 = 30
Blackcurrant, mango*	53	20 + 4 = 24	42 + 8 = 50
Bc–MSG, grape–IMP	24	5 + 6 = 11	16 + 9 = 25
SUM	325	139 (43%)	263

Tested neurons refers to task-related neurons in both animals, as assessed by significance in the Wilcoxon and one-factor ANOVA tests (*P* < 0.01) against pretrial control activity. In table cells with multiple entries, the first two numbers refer, respectively, to positive and negative (inverse) relationships to increasing reward quantity, as inferred from the regression slope of neuronal coding (β in Eq. (4)). Revealed preference coding was defined by a combination of three statistical tests (see Methods: Statistical analysis of neuronal revealed preference coding): (1) multiple linear regression, Eq. (4), across indifference curves (IC): *P* < 0.05 (*t* test), (2) Spearman rank-correlation across ICs: *P* < 0.05, (3) two-factor ANOVA: *P* < 0.05 across-IC, *P* > 0.05 within-IC and *P* > 0.05 interaction. During choice over zero-reward bundle, one additional neuron showed significant changes between bundles across-IC (*P* < 0.05; two-factor ANOVA) but not within-IC (*P* > 0.05), but failed to show significant changes with Eq. (4) and/or Spearman correlation; during choice between two nonzero bundles, ten additional neurons showed equivalent changes. Each response derived from multiple trials in one neuron in one of the four epochs, and regressed significantly on all bundles tested on that neuron. Thus, a given neuron could have distinct responses in more than one task epoch (the four task epochs were Bundle stimulus, Go signal, Choice, and Reward); therefore, the number of significant responses typically exceeded the number of significant neurons. A neuron was designated as revealed preference neuron if it had a significant response in our three-test statistics in at least one of the four task epochs. *The data collapsed from Monkeys A and B

Trial-by-trial oculomotor analysis demonstrated significant, albeit weak, gaze relationships in 11 of the 441 task-related OFC neurons (2.4%; Supplementary Fig. 14; rho = 0.2 ± 0.2, mean ± standard error of the mean, SEM, *P* < 0.001; Spearman rank-correlation), which was less than during free gaze observed previously (8.8%)^35^.

### Revealed preference coding during choice between two nonzero bundles

As preference is revealed by choice, we investigated all OFC neurons during choice between two nonzero bundles. The revealed preference neuron in Fig. 2d, e showed stronger responses to the more frequently chosen bundle (choice probability *P* = 0.7), and weaker responses to its alternative (*P* = 0.3; brown vs. blue); responses were similar with equally preferred bundles on the same IC (*P* = 0.5 choice of each bundle; Fig. 2f). The responses conformed to the decision variable of chosen value, which reflects the value of the chosen option^16,33,34^ (Eq. (13)). The response coded absolute chosen value by failing to differ when the unchosen options varied and, accordingly, the choice probabilities differed (*P* = 0.7/*P* = 0.3; choice between two nonzero bundles, and *P* = 1.0/*P* = 0.0; choice over zero bundle) (Fig. 2d, solid vs. dotted lines). These neurons coded the chosen value of both bundle components combined rather than of single rewards reported before^16,31,32^.

In total, during choice between two nonzero bundles, 322 responses in 152 of 391 task-related OFC neurons (39%) coded revealed preference, including all 139 neurons responding during choice over zero bundles (Table 2) (13 neurons failed to code revealed preference during choice over zero bundle, of which 7 neurons coded only single reward A and 6 neurons coded only single reward B). Regressions with interaction (Eqs (7–9)) yielded higher adjusted R2s in these 152 neurons (*P* < 0.05; Fisher’s test; Supplementary Tables 4, 5). Spearman rank-correlations across ICs yielded mean rho = 0.6917 for 107 positive and rho = −0.6234 for 45 inverse coding neurons (*P* < 0.05). By contrast, additional 57 of the 391 task-related neurons (15%) showed significant response changes both across and along ICs, and thus failed to code revealed preference.

**Table 2:** Revealed preference coding during choice between two nonzero bundles

Bundle type	Tested neurons	Neurons responding	Responses
Blackcurrant, grape	89	32 + 6 = 38	53 + 12 = 65
Blackcurrant, water*	159	32 + 18 = 50	67 + 36 = 103
Blackcurrant, apple	35	16 + 7 = 23	30 + 11 = 41
Blackcurrant, mango*	70	20 + 8 = 28	63 + 20 = 83
Bc–MSG, grape–IMP	29	7 + 6 = 13	16 + 14 = 30
SUM	391	152 (39%)	322

For conventions, see Table 1

Chosen value coding, as shown in Fig. 2d–f, occurred irrespective of alternative options (absolute chosen value; 110 responses in 51 of the 152 revealed preference neurons, 34%) or relative to the unchosen option (43 responses in 18 of the 152 neurons, 12%) (Table 3), based on our conservative analysis with linearly combined reward quantities (Eq. (13) with Eqs (11) and (12); see Methods: Neuronal chosen value coding). These numbers were slightly higher when taking the nonlinear nature of ICs into account (Eq. (13) with Eqs (11a) and (12a); Table 3). Other OFC neurons coded the value of the unchosen option and the total value of both options (Table 3). The extended regression (Eq. (14)) confirmed coding of chosen value, but not of previous choice or bundle stimulus position (Supplementary Fig. 13b). Polar plots characterize chosen value coding types and indicate prevailing chosen value responses to the bundle stimuli and at choice, extending to reward, but less often with the Go signal (Supplementary Figs. 15, 16).

**Table 3:** Chosen value coding of bundles

	Neurons (linear)	Responses (linear)	Neurons (interaction)	Responses (interaction)
Absolute chosen value	42 + 9 = 51 (34%)	94 + 16 = 110 (34%)	49 + 13 = 62 (41%)	112 + 17 = 129 (40%)
Relative chosen value	11 + 7 = 18 (12%)	27 + 16 = 43 (13%)	11 + 3 = 14 (9%)	35 + 3 = 38 (12%)
Unchosen value	17 + 22 = 39 (26%)	30 + 48 = 78 (24%)	26 + 30 = 56 (37%)	56 + 65 = 121 (38%)
Total value	15 + 2 = 17 (11%)	35 + 3 = 38 (12%)	13 + 3 = 16 (11%)	20 + 10 = 30 (9%)
Tested	152	322	152	322

The data are from both animals, all bundle types and choice between two nonzero bundles. Absolute chosen value refers only to the option the animal is choosing, relative chosen value refers to the difference chosen value minus unchosen value; unchosen value refers only to the option the animal is not choosing; total value is chosen value plus unchosen value. The type of chosen value coding was inferred from the significance of the neuronal coding slope coefficients (β) in regression Eq. (13) (*P* < 0.05; *t* test) during binary choice between two nonzero bundles. Chosen and unchosen value were estimated from Eqs (11) and (12) with conservative, linear combination of reward quantities (left two columns), and from Eqs (11a) and (12a) with quantity interaction (right two columns). There were 5–15% more chosen value neurons when revealed preference coding was tested with Eq. (7) alone instead of our three-test statistics including Eq. (4). In table cells with multiple entries, the first two numbers refer, respectively, to positive and negative relationships to increasing chosen value, as inferred from the sign of neuronal coding slope coefficients β (Eq. (13)). For conventions, see Table 1

### Population properties of revealed preference neurons

**Fig. 3:**
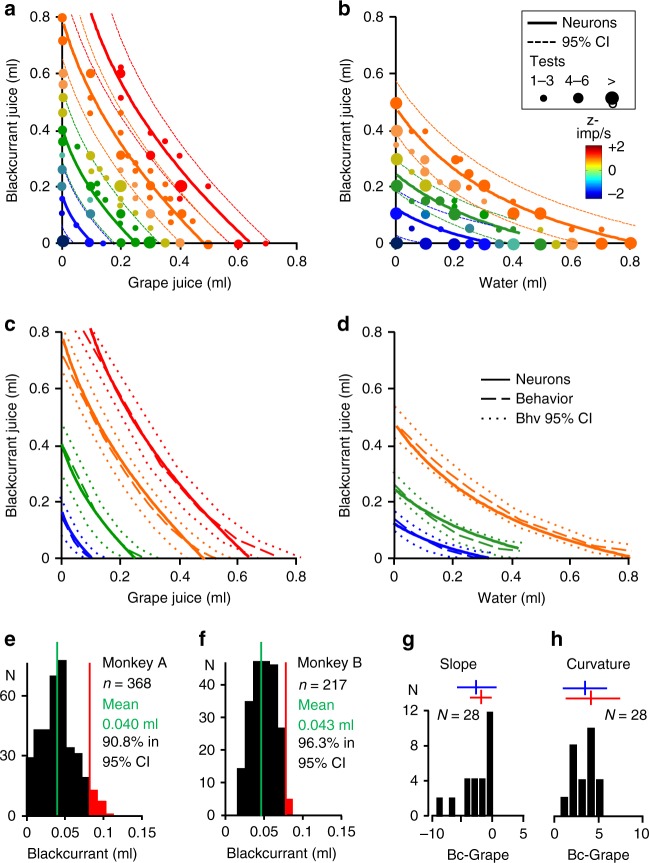
Neuronal indifference curves (IC). **a** Neuronal ICs for bundle (blackcurrant, grape). Each dot indicates 1 to >6 tested bundles (*x*–*y* coordinates: liquid quantities; color: response strength). Each IC fits neuronal revealed preference responses of similar strength (same color dots; ± 95% CI, confidence interval; plotted from Eq. (7b)). ICs were estimated from 37 positive coding responses in any task epoch of 22 revealed preference neurons (choice over zero-reward bundle, 78 bundles; Monkey A; dots: tested bundles; Table 1; Supplementary Table 6; see Methods: Population plots 5a). Color binning reflects ordinal, step-wise property of ICs. z-imp/s: impulses/s in task epoch analysis window, z-score normalized to pretrial control. **b** As **a** but for bundle (blackcurrant juice, water), using 36 positive coding responses of 23 revealed preference neurons (82 bundles). **c, d** Correspondence between neuronal ICs and behavioral ICs. Bundles and responses as **a, b**, respectively. Behavioral ICs were constructed from hyperbolic fits (Eq. (1)). **e, f** Out-of-sample correspondence between neuronal and behavioral ICs for the two animals. Average difference between z-scored neuronal responses to new out-of-sample bundles and established neuronal ICs was measured in blackcurrant juice quantity (reward A, *y*-axis; 0.040 ml and 0.043 ml for 368 and 217 positive- or inverse coding neuronal responses to 238 and 158 new bundles during choice over zero-reward bundle or between two nonzero bundles; blackcurrant vs. grape and blackcurrant vs. water). Vertical red lines indicate ± 95% CI; *n* *=* number of responses. **g** The corresponding neuronal and behavioral IC slope (currency) parameters for bundle (blackcurrant, grape). Vertical bars: mean; horizontal bars: ± 95% CI. Blue: neurons (β_2_/β_1_; Eq. (7)). Red: behavior (Eq. (1)). *n* *=* number of neurons with 45 positive- or inverse coding responses in any task epoch (choice over zero-reward bundle; Monkey A). The neuronal responses reflected the behaviorally estimated lower value of blackcurrant relative to grape juice (steeper slope than −1; mean ~−2). For all other bundle types, see Supplementary Fig. 19. **h** As **g** but for curvature (utility gained (>1) by combined bundle rewards, requiring less quantity for choice indifference). Blue: neurons (β_3_, Eq. (7)). Red: behavior (Eq. (1))

The responses to several hundred bundles with different juice combinations conformed to maps of neuronal ICs (Fig. 3a, b; Supplementary Table 6; see Methods: Neuronal population plots 5a): every response was similar to every other response to bundles on same-colored neuronal ICs; responses increased monotonically (or decreased with inverse coding) across neuronal ICs (from blue to orange to red), even when higher IC bundles contained one smaller reward than lower-IC bundles. The neuronal ICs failed to overlap with other neuronal ICs and followed closely the behavioral ICs within their 95% confidence intervals (Fig. 3c, d). This was seen for positive and for inverse coding responses, with bundles showing left-leaning or right-leaning slopes and convex, linear, or concave curvatures, during choice over zero-reward bundle and during choice between two nonzero bundles (Fig. 3a–d; Supplementary Fig. 17) and in specific task epochs (Supplementary Fig. 18). Thus, neuronal ICs matched well the behavioral ICs.

To validate the neuronal ICs, we used out-of-sample tests with 585 neuronal responses to new 396 bundles not used for establishing neuronal ICs. We computed the vertical (*y*-axis) distance between the new bundle and the neuronal IC with the most similar neuronal response strength (indicated by matching colors; Fig. 3a–d; see Methods: Population plots 5b). More than 90% of the 585 responses were within the 95% confidence interval of same color neuronal ICs in both animals (Fig. 3e, f). These results confirmed robust bundle representation by neuronal ICs.

To scrutinize numerically the match between neuronal ICs and behavioral ICs, we compared the defining parameters slope (currency) and curvature (separately, avoiding false matches from mutually canceling differences). We estimated behavioral IC slope and curvature from hyperbolic coefficients b and a, respectively (Eq. (1)). We estimated neuronal IC slope from regression coefficient ratio β_2_/β_1_, and neuronal IC curvature from interaction coefficient β_3_ of Eq. (7). Means of neuronal IC slopes and curvatures fell within the 95% confidence intervals of the respective behavioral IC parameters, and vice versa, for positive and for inverse coding responses, separately for all five bundle types, and in both animals (Fig. 3g, h; Supplementary Fig. 19); differences between neuronal and behavioral parameters were insignificant (slope: *P* = 0.1747 to *P* = 0.6952; curvature: *P* = 0.1723 to *P* = 0.88, *t* test). Using a separate method for robustness, we also assessed behavioral IC slopes from anchor bundles at *x*- and *y*-axes psychophysically, and estimated neuronal IC slopes from coefficient ratio β_2_/β_1_ of Eq. (4). The neuronal IC slopes correlated well with behavioral IC slopes (rho = 0.369; *P* = 8 × 10^−6^, Pearson; rho = 0.390, *P* = 2 × 10^−5^, Spearman; Supplementary Fig. 20). These numeric measures substantiated quantitatively the graphic correspondence between neuronal ICs and behavioral ICs shown in Fig. 3a–d.

### Bundle and choice decoding

**Fig. 4:**
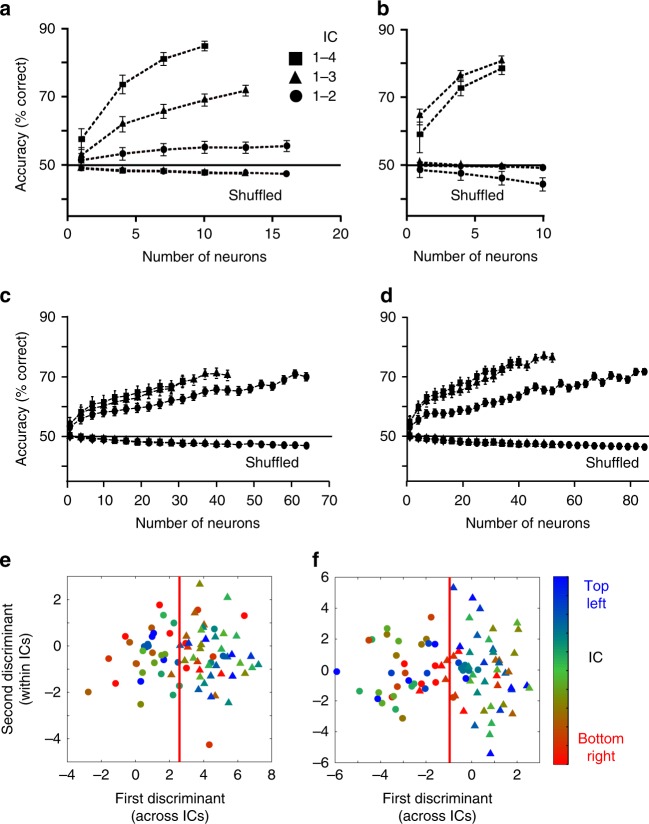
Bundle decoding. **a** Support vector machine decoding of bundle distinction between indifference curves (ICs 2–4, farther from origin, vs. IC1, closest to origin; z-scored revealed preference responses to bundle stimulus; choice over zero bundle; *n* *=* 38 bundles; *P* < 10^−20^ against the shuffled data; Wilcoxon rank-sum test). Bundles on ICs 2–4 had one smaller component than bundles on IC1. Decoding within same ICs was insignificant (IC1: *P* = 0.34 ± 0.11, mean ± SEM from 150 repetitions of 45 comparisons against the shuffled data; IC4: *P* = 0.23 ± 0.05; Supplementary Tables 7, 8). **b** As **a** but choice prediction between two nonzero bundles (*n* *=* 54 bundles; *P* < 10^−20^). Insignificant encoding within ICs (IC1: *P* = 0.24 ± 0.06; IC4: *P* = 0.26 ± 0.04; Supplementary Tables 9, 10). **c** As **a** but bundle distinction by unmodulated neuronal activity (neither bundle nor single-reward coding) following bundle stimulus. One component of bundles on ICs 2–4 had one larger, equal, or smaller component than IC1 bundles (*n* *=* 129 bundles; *P* < 10^−20^). **d** As **c** but choice prediction between two nonzero bundles (*n* *=* 225 bundles; *P* < 10^−20^). **e** Visual bundle distinction IC1 vs. IC4 using linear discriminant analysis of z-scored bundle stimulus revealed preference responses (*n* *=* 12 neurons; choice over zero bundle). IC4 bundles had one smaller component than IC1 bundles. Significant decoding across ICs (dots vs. triangles, red line separating ICs; five random selections of 10 bundle response trials each; *P* < 10^−5^ against the shuffled data; Wilcoxon rank-sum test), but not within ICs (colors indicate bundle position from top left to bottom right of ICs; IC1: *P* = 0.232 ± 0.107, *n* *=* 6 bundles; IC4: *P* = 0.089 ± 0.029, *n* *=* 10 bundles; Supplementary Tables 11, 12). **f** As **e** but choice prediction between two nonzero bundles (*n* = 6 neurons). Choice prediction was numerically significant across ICs (*P* < 10^−33^ against the shuffled data), but not within ICs (IC1: *P* = 0.162 ± 0.045, *n* *=* 8 bundles; IC4: *P* = 0.098 ± 0.031, *n* *=* 10 bundles; Supplementary Tables 13, 14)

We used classifiers to further test whether the neuronal responses followed the graphic formalism of Revealed Preference Theory, namely bundle distinction and prediction across ICs, but not on same ICs. We applied a linear support vector machine (SVM) to our identified revealed preference responses (see Methods: Neuronal decoders). Decoding accuracy for bundle distinction (during choice over zero-reward bundle) and choice prediction (during choice between two nonzero bundles) exceeded 80% across ICs when higher IC bundles had two larger rewards, or one smaller reward, than lower-IC bundles (Fig. 4a, b), and in all four task epochs (Supplementary Figs. 21, 22) (*P* < 10^−20^ against shuffled data; Wilcoxon rank-sum test). Accuracy increased meaningfully with neuron number and increasing IC distance. By contrast, accuracy was at chance level along same ICs (*P* > 0.2; pairwise bundle comparisons; Supplementary Tables 7–10), suggesting lack of distinction between equally revealed preferred but differently composed bundles. Accuracy was lower, yet still significant, with unmodulated neuronal responses coding neither bundles nor single-rewards (Fig. 4c, d; Supplementary Fig. 23a, b). With all responses irrespective of modulation, accuracy of choice prediction between ICs was intermediate (for same neuron numbers) (Supplementary Fig. 23c) between that for modulated and unmodulated activity (Fig. 4b vs. d), but remained at chance along same ICs (e.g., along lowest IC: *P* = 0.0924 ± 0.0172, mean ± SEM; highest IC: *P* = 0.188 ± 0.031).

To visualize bundle distinction and choice prediction, we performed linear discriminant analysis (LDA). Using the neuronal responses, LDA discriminated bundles numerically and graphically across ICs (*P* < 0.0001, Wilcoxon rank-sum test against the shuffled data; Fig. 4e, f; dots vs. triangles), but not along ICs (*P* > 0.08; colors; pairwise bundle comparisons; Supplementary Tables 11–14). This differential discrimination pattern was also seen with entirely unselected neuronal responses (Supplementary Fig. 23d). Thus, LDA confirmed the SVM results, including bundle indiscriminability within ICs.

Thus, the decoders using scalar neuronal signals accurately distinguished vectorial bundles and predicted choice across ICs from neuronal responses, thus confirming the validity of the neuronal revealed preference code. Given this discriminatory accuracy, the decoders found no distinctions between bundles located on same ICs during choice over zero bundle and choice between two nonzero bundles.

### Arrow’s Weak Axiom of Revealed Preference (WARP)

**Fig. 5:**
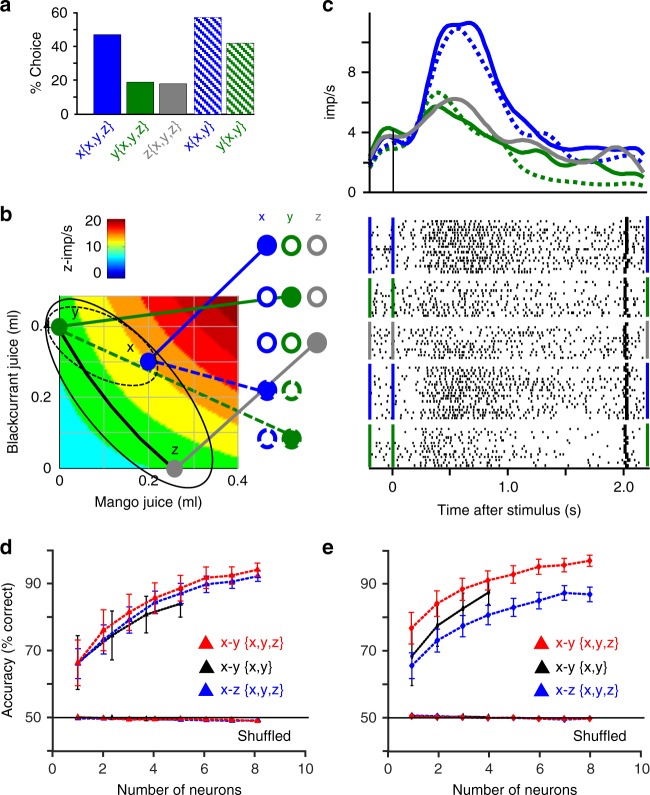
Testing Arrow’s Weak Axiom of Revealed Preference (WARP). **a** Behavioral compliance: bundle **x** remained revealed preferred (**x**{**x, y, z**}) when restricting the three-bundle set {**x, y, z**} to two bundles {**x, y**} (bundle **x** was chosen with highest probability in both sets). Bars show % choice while recording the neuron shown in **b, c** (*n* *=* 83 choices between two nonzero bundles of (blackcurrant, mango)). **b** Positions of test bundles on behavioral indifference curve (**y, z**) and above (**x**). Colored heatmap bands show bundle responses of this neuron (plotted from Eq. (7b); increasing from turquoise to red). Elipsoids and lines refer to choice between three bundles ({**x, y, z**}; solid) and choice between two bundles ({**x, y**}; dotted). **c** Neuronal responses of revealed preference neuron to revealed preferred bundle x remained highest despite restriction from three-bundle set {**x, y, z**} (solid blue) to two-bundle set {**x, y**} (dotted blue), compared with alternative bundles **y** and **z** (green, gray). The chosen value response was higher with choice of revealed preferred bundle **x** (blue) compared with choice of alternative bundles **y** (green) and **z** (gray), even when bundle **x** contained one smaller reward. Filled dots at left indicate chosen bundles. **d, e** Similar Support vector machine decoding with three-bundle set (bundles **x** vs. **y** in {**x, y, z**}; red) as with restricted two-bundle set (bundles **x** vs. **y** in {**x, y**}; black). The SEMs for bundle stimulus decoding overlapped between the two bundle sets (red and black): **d** distinction during choice over zero-reward bundle; **e** choice prediction between two nonzero bundles. Blue: comparable decoding of bundles **x** vs. **z** (all bundle decoding *P* < 10^−20^ against the shuffled data; Wilcoxon rank-sum test)

Arrow’s WARP defines a necessary condition for optimal choice and utility maximization^36^. An option that is revealed preferred to all other options in a given option set should remain revealed preferred when the option set is reduced to a smaller subset by removing one or more of the alternative options. In general compliance with Luce’s stochastic choice theory^29^, we asked whether the same bundle that is revealed preferred to all other options within a set of three nonzero bundles {**x, y, z**} (choice *P* > 0.33) would remain revealed preferred within a subset of two nonzero bundles {**x, y**} (choice *P* > 0.5). This requirement was satisfied with convex ICs (Fig. 5a, b) and concave ICs (Supplementary Fig. 24a, b)^27^.

For neuronal compliance with Arrow’s WARP, the revealed preferred bundle x eliciting the strongest neuronal response in the three-bundle set {**x, y, z**} should elicit also the strongest response in the restricted two-bundle set {**x, y**}. Indeed, 30 of 56 tested neurons (54%) showed significantly stronger chosen value responses to bundle **x** compared with bundle y in both {**x, y, z**} and {**x, y**} nonzero bundle sets (90 responses) (*P* < 0.002 for bundle factor **x** vs. **y** and **z** in two-factor ANOVA; *P* < 0.0001 in Spearman rank-correlation), with convex ICs and concave ICs, even when one reward in the alternative bundle **y** (or **z**) was larger than in the revealed preferred bundle **x** (Fig. 5b, c; Supplementary Fig. 24b, c). The responses to the revealed preferred bundle **x** varied insignificantly between three- and two-bundle sets in 16 of the 30 neurons (*P* > 0.05; factor {**x, y, z**} vs. {**x, y**} bundle set in two-factor ANOVA), suggesting absolute chosen value coding. An SVM decoder using responses of 10 of the 16 neurons showed high bundle distinction (choice over zero-reward bundle) and choice prediction (choice between two nonzero bundles) with both three- and two-bundle sets (Fig. 5d, e). Responses differed significantly in the remaining 14 neurons between three- and two-bundle sets (*P* < 0.01) but, importantly, remained strongest to the commonly revealed preferred bundle **x**. Together, most OFC revealed preference signals complied with behavioral WARP as necessary condition for optimal choice.

### Single-reward coding

**Fig. 6:**
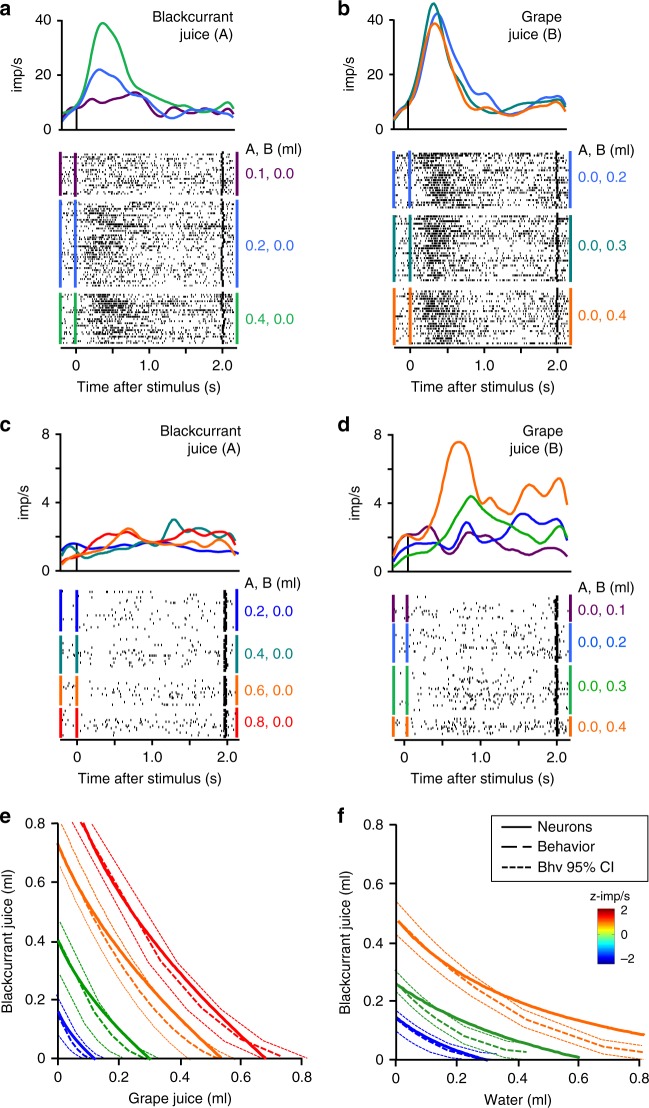
Single-reward coding aggregating into scalar population signals for vectorial bundles. **a** Increasing responses in a single neuron with blackcurrant juice quantity (juice A, from red to green). A, B (ml) refers to respective quantity of juices A, B of the nonzero bundle chosen over the zero-reward bundle. **b** Invariant responses to grape juice (juice B) (same neuron as **a**). **c** Completely insensitive activity with blackcurrant juice in a different neuron. **d** Increasing responses with grape juice (same neuron as **c**). **e, f** Neuronal population ICs derived from aggregated individual, single-reward responses. Neuronal ICs (solid lines) were estimated from positively coding, nonsimultaneously recorded responses and aligned same neuronal responses to different bundles (plotted using Eq. (7b) from all single-reward coding neurons initially identified by Eqs (5) and (6). Behavioral ICs (dotted lines) were plotted from hyperbolic fits (Eq. (1)). ICs in **e, f** were estimated from respective 34 and 40 responses in any of the four task epochs from 24 and 25 single-reward positive coding neurons in Monkey A (z-imp/s: response strength)

A distinct set of OFC neurons coded the quantity of only one of the two bundle rewards (*n* *=* 144 neurons with 246 responses during choice over zero-reward bundle, and 223 neurons with 432 responses during choice between two nonzero bundles, identified by regression Eqs (4–6); see Methods: Population plots 5c; Supplementary Tables 15,16). Single-reward coding occurred with all five tested bundle types and in all four task epochs (Supplementary Figs. 11, 12). It came in two versions: response to both rewards, but response increase (or decrease with inverse coding) with quantity of only one reward (Fig. 6a, b), or exclusive response and variation with only one reward (Fig. 6c, d). During choice between two nonzero bundles, up to 38% of single-reward responses reflected some form of chosen value (Supplementary Table 17). Comparable, so-called lexicographic behavioral choices following only a single reward were not observed, indicating that these single-reward responses were not explained by sporadic behavioral changes (Supplementary Fig. 2).

When analyzing all single-reward responses together (while excluding revealed preference neurons coding both bundle components), we found that their aggregated population signal followed well the revealed preference scheme; their neuronal ICs showed the same left-leaning or right-leaning slopes and similar curvatures as the behavioral ICs; they followed the behavioral ICs close to, and often within, their confidence intervals (Fig. 6e, f), although slightly less closely than populations of single, fully revealed preference coding neurons (Fig. 3a–d). Thus, single-reward coding, scalar OFC responses can aggregate into population signals for vectorial bundles.

We constructed neuronal ICs from the total of all positive and inverse preference responses and single-reward responses, which together comprised >85% of all task-related OFC neurons (Tables 1, 3). The neuronal ICs overlapped with the behavioral ICs within their 95% confidence intervals for the two most tested bundle types (Supplementary Fig. 25). Thus, although inverse coding responses conceivably reduced the averaged population signal, a substantial revealed preference population signal emerged from the large majority of task-related OFC neurons.

## Discussion

**Fig. 7:**
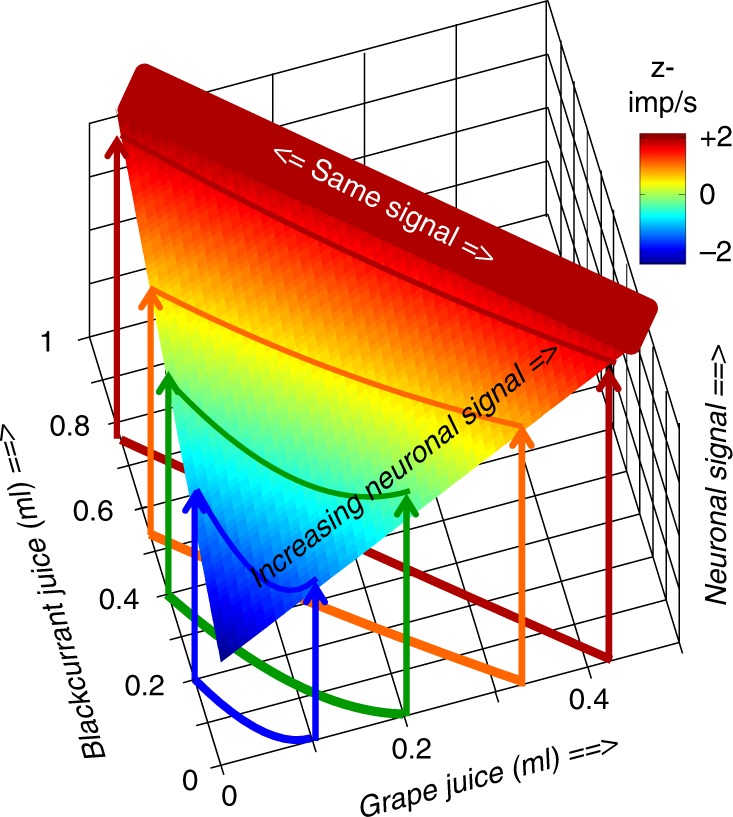
A scalar neuronal signal for two-dimensional, vectorial bundle choice options. Color-coded intensity of neuronal revealed preference response in orbitofrontal cortex: same signal with equal revealed preference along indifference curves (indicated by same color), increasing signal with increasing behavioral revealed preference across indifference curves (from blue to red). Single neuron, choice between two nonzero bundles (600 trials; z-imp/s: z-scored impulses/second)

To investigate how scalar neuronal responses emerge from vectorial bundles, our experimental design employed concepts of Revealed Preference Theory and used different quantities of the same two reward types in both choice options. We found two forms of neuronal signals. The integrated form followed the characteristic two-dimensional ICs: differently composed but equally revealed preferred bundles located on same ICs elicited indistinguishable neuronal responses, reflecting the graded, fractional, and continuous component trade-off within bundles; revealed preferred bundles on higher ICs elicited stronger neuronal responses than bundles on lower ICs, even when one bundle component was smaller in the preferred bundle than in its alternative (partial physical non-dominance). These neuronal responses formed well-ordered ICs that matched visually and numerically the behavioral ICs (Fig. 7). The neuronal signal was robust; it occurred with all tested rewards, bundle types and task epochs during asymmetric choice over zero-reward bundle and during choice between two nonzero bundles, satisfied out-of-sample prediction, decoded bundles and predicted their choice, and complied with Arrow’s WARP as necessary condition for utility maximization. In the second form, individual OFC neurons coded only single rewards, but their aggregated population signal formed well-ordered neuronal ICs. These signals implemented basic concepts of Revealed Preference Theory in neuronal hardware, which makes the concepts biologically plausible and allows future neuronal investigations of economic choice and its violations.

The individual revealed preference responses, and the populations responses of single-reward neurons, demonstrate the emergence of scalar neuronal responses from vectorial bundles. At trade-off and with partial physical non-dominance, the same and a larger neuronal signal, respectively, result from oppositely varying bundle components, thus showing the integration of both components of the vectorial bundle into a scalar signal.

The OFC signals followed in four ways the characteristic ICs for multi-component choice options, as graphically formalized by Revealed Preference Theory. First, neuronal responses were similar, and varied only insignificantly, for bundles located on same ICs. As our ANOVA was sensitive enough to detect neuronal changes across ICs, its insignificant along-IC factor suggests response similarity despite varying bundle composition (trade-off). Second, neuronal responses changed monotonically across ICs even when one component in a bundle on a higher IC was lower than in a bundle on a lower IC, reflecting overcompensation by the other bundle component (partial physical non-dominance). Third, the curvature of neuronal ICs paralleled that of behavioral ICs, thus following the fine, local variations in graded trade-off. Thus, with a convex IC, a bundle located on a higher IC in the center of a straight line elicited stronger responses than bundles on a lower IC at line end (Fig. 2a, b). Fourth, while SVM and LDA decoded the presented bundles and predicted the animal’s choice, both classifiers found no distinction between equal-preference bundles on same ICs. These four results capture the emergence of scalar measures from vectorial choice options

In contrast to our design with different quantities of the same two reward types in both choice options, previous psychophysical assessments of choice indifference used a different reward type in each choice option^16,20^. One reward with specific quantity was compared with another reward with a specific quantity in the other option. In our graphs, at choice indifference, such two equally preferred rewards of 0.4 ml of blackcurrant juice and 0.2 ml of grape juice would be located at distinct positions of the two-dimensional plot (Supplementary Fig. 26a), but without forming a connecting IC that represents the graded trade-off typical for revealed preference concepts. When plotted in our graphic convention (Fig. 1a, b), the two psychophysical IPs are positioned along the *x*- and *y*-axes of the two-dimensional graph, but not in its interior (Supplementary Fig. 26b). This scenario has an equivalent in our experiment when only one component in each bundle varied during IP estimation and the other component had zero quantity (Fig. 1d). Supplementary Fig. 26c, d shows corresponding bundle stimuli in the format of our displays (Fig. 1c). These previous studies^16,20^ tested the subjective equivalence between two different choice options. However, their approach does not allow to assess the graded, fractional and continuous trade-off within each choice option that underlies the integration of components of vectorial, multi-component bundles into a scalar variable as conceptualized and graphed by Revealed Preference Theory. Such a vector-to-scalar transformation is precisely the reason for varying multiple components in each choice option.

Further, previous multi-reward sensitive OFC and dopamine responses integrated the common-currency value from different rewards and changed monotonically with reward quantity (U-shaped profile^16,20,21^. Maybe their responses would have reflected the graded trade-off in being indistinguishable between the bundles that lined up as IPs of ICs, as found presently, had they been tested accordingly. However, almost one-third of our multi-reward sensitive OFC neurons showed an incompatible pattern; they changed responses both across ICs and between bundles on same ICs, and hence failed to follow the equal preference that defines an IC. Thus, multi-reward sensitivity is not necessarily associated with the graphic IC formalism.

Further, previously reported multi-reward sensitive OFC responses reflected the value of the imposed or chosen reward^15,16,24^. Chosen value refers both to the option the animal is choosing and to the value of that option. The present study reports chosen value responses in two ways: First, in revealed preference neurons, which were sensitive to both bundle components, chosen value refers to the integrated, scalar value of the whole bundle. Second, in single-reward neurons, chosen value refers only to the value of the specific reward the neuron is coding. Thus, chosen value coding may occur with any form of choice option irrespective of how many components it contains.

Further, the use of multi-component bundles allowed us to distinguish chosen value coding from multi-reward sensitivity. A multi-reward sensitive neuron might have a weak response when the animal chooses the low-value reward and a strong response when choosing the other, high-value reward, thus seemingly coding chosen value. However, the responses would look exactly the same if the neuron coded only the value of both rewards irrespective of choice, which would amount to choice-independent object value coding. By contrast, our use of the same two reward types in both choice options allowed the distinction: about one fifth of our multi-reward sensitive revealed preference neurons failed to code chosen value and defied simple categorization or coded total value; reversely, one half of our single-reward coding OFC neurons coded chosen value, analogous to previous findings^16,24,31,37–41^. Thus, not all multi-reward-sensitive OFC neurons coded the chosen value, and not all chosen values coding OFC neurons were multi-reward sensitive.

Further, our single-reward coding neurons may correspond to previously reported offer value neurons that code the value of either one or the other component, but not both (L-shaped or anti-L-shaped profiles of offer value neurons^16^. However, there are two distinctions. First, our use of the same two rewards in both choice options uncovered that some single-reward coding responses reflected chosen value (Supplementary Tables 15–17), rather than the reported offer value. Second, our single-reward coding responses aggregated into a population signal that reflected the scalar value of vectorial bundles by conforming to neuronal ICs, rather than simply coding reward value on a common-currency scale. Thus, our design with different quantities of the same two reward types in both choice options identified a rich repertoire of coding multi-component choice options and facilitated disambiguation of neuronal decision variable coding.

The SVM and LDA decoded the presented bundles and predicted behavioral choices from neuronal responses. The decoding accuracy may surpass that of nearest neighbor classifiers for visual stimulus identification^42^ and choice and learning prediction^43–45^. Decoders work opposite to standard statistics: regressions, correlations, and ANOVAs use independent external variables (reward bundles and choices) to explain the dependent neuronal responses, whereas decoders use neuronal responses as independent variables to discriminate and predict dependent external variables (reward bundles and choices). Our decoders distinguished bundles and predicted choices from neuronal responses; their accuracy increased meaningfully with the number of neurons and, importantly, was substantial even when one component of the revealed preferred bundle was smaller than in its alternative. These results suggest a neuronal signal with sufficient accuracy to potentially shape the animal’s behavior. Interestingly, decoding accuracy was still appreciable with responses showing neither significant revealed preference nor single-reward coding and, as a control, with entirely unselected responses, which suggested appreciable population coding below single-neuron statistical thresholds.

Our monkeys’ choices complied with Arrow’s WARP when we removed a non-preferred option from the three-bundle set^27^. Our OFC responses followed Arrow’s WARP by staying strongest with the bundle that remained revealed preferred when reducing the three-bundle set to two bundles. The observation that the responses varied only insignificantly between the two bundle sets in one-half of these OFC neurons might suggest robust coding of revealed preference. By contrast, the changed responses in the other half of neurons may partly reflect the number of bundle options or the slightly varying, but rank-maintaining choice probability. The SVM decoding remained significant, despite bundle set reduction, and thus supported the neuronal compliance. The stability of the neuronal revealed preference signal between bundles of different set size contrasts with the frequent violations when extending two-bundle sets to three-bundle sets in humans (similarity effect, compromise effect, asymmetric dominance effect, attraction effect^2,3^). The failure to see such behavioral violations in our monkeys^27^ may be due to the long-term nature of the experiment during which the animals might have recognized smaller distributions as parts of the full distribution and failed to adapt, as seen before with more simple choices^46,47^. Thus, in this stable situation, OFC neurons coded revealed preference irrespective of what else was on offer, suggesting their responses complied with utility maximization in this situation.

The single-reward responses failed individually to code revealed preference, but did so as a population. The signal emerged from the combined regression coefficients of the single-reward responses to the two bundle rewards, matched the behavioral ICs around their 95% confidence intervals, and followed the formalism of Revealed Preference Theory: same strength with bundles along ICs, despite varying bundle composition, increase across ICs even with one smaller bundle component, and non-diagonal slope and nonlinear curvature. Further work should elucidate the underlying mechanisms; econometric analyses could provide out-of-sample tests for the precision of the aggregate population code, and optogenetic stimulation could unravel the contributing circuitry. Being a population signal, it should exist also in human neuroimaging and could be used for translational studies on decision disorders, such as gambling, drug addiction, and obesity.

The observation that individual OFC neurons coded revealed preference for bundles either in an integrated or in a segregated fashion may inform the debate about the nature of economic choice of multiple-component options. In some situations, decision-makers may integrate bundle components with particular weights rather than according to their respective, common-currency utilities^1,3,5,48^. Reasons for these heterogeneous findings may include unequal attention to individual components and sensory discrimination limits. The current two forms of bundle coding might provide a neuronal basis for the different ways multi-component choices are made and may go wrong.

## Methods

### Animals

Two adult male macaque monkeys (*Macaca mulatta*; Monkey A, Monkey B), weighing 11.0 kg and 10.0 kg, respectively, were used in the experiments. Neither animal had been used in any other study; the behavioral results have been published in detail^27^.

### Ethical approval

This research has been ethically reviewed, approved, regulated, and supervised by the following UK and University of Cambridge (UCam) institutions and individuals:

- UK Home Office, implementing the Animals (Scientific Procedures) Act 1986, Amendment Regulations 2012, and represented by the local UK Home Office Inspector
- UK Animals in Science Committee
- UK National Centre for Replacement, Refinement and Reduction of Animal Experiments (NC3Rs)
- UCam Animal Welfare and Ethical Review Body (AWERB)
- UCam Biomedical Service (UBS) Certificate Holder
- UCam Welfare Officer
- UCam Governance and Strategy Committee
- UCam Named Veterinary Surgeon (NVS)
- UCam Named Animal Care and Welfare Officer (NACWO).

### Implementation of basic concepts

We modeled the multi-component nature of choice options as vectorial bundles that contained the same two distinct, independently variable, scalar liquid rewards. We presented the animal with an option set of two bundles, the Reference Bundle and the Variable Bundle, that appeared simultaneously at equal reaching distance; only our axiomatic tests employed three-bundle option sets. Thus, in keeping with general notions of discrete choice models, the employed choice sets were exhaustive and had finite numbers of options (two or three), and their options were mutually exclusive. The statistical analysis of neuronal responses requires the use of multiple trials. Therefore, we made our design compatible with basic assumptions of stochastic choice theories^13,28,29^ (although testing Luce’s probability ratio formalism would have required trial numbers beyond the scope of this study^27^). Thus, we assessed revealed preference from the probability of multiple choices, rather than by traditional single-shot economic tests.

We implemented the following notions of Revealed Preference Theory:

1. Revealed preference indicates a relation between all options in a choice set and depends on all components of all bundles.
2. Revealed preference for a given bundle is inferred from the measured probability of choice of that bundle over all alternative bundles in the same option set during multiple trials. We inferred revealed preference from a choice probability of *P* > 0.5 with two options and *P* > 0.333 with three options
3. Increasing quantities of one component of one bundle, with all other components constant, leads to monotonically increasing probability of choosing this bundle over its alternative, as modeled by an S-shaped psychophysical choice function.
4. Bundles are equally revealed preferred, and inferred to have equal utility for the animal, when the animal chooses them with equal probability (*P* = 0.5 each in a two-option set; *P* = 0.333 each in a three-option set).
5. Every bundle has a subjective value for the decision maker, called utility, that depends only on the reward quantities of both juice components. A bundle is chosen with a higher probability than any other bundle in the same option set if and only if its utility for the animal is higher than that of any other bundle in that option set. A bundle that is chosen with equal probability against another bundle is assumed to have the same utility as the other bundle. These choices were complete; our previous report showed that they were also transitive^27^.
6. A bundle that is revealed preferred to all other bundles in a given option set should remain revealed preferred when one or more of the alternative bundles are removed from the option set (Arrow’s Weak Axiom of Revealed Preference^36^).
7. Each two-component bundle is graphically represented at the intersection of the *x*-coordinate (reward B) and *y*-coordinate (reward A) of a two-dimensional plot (Fig. 1a).
8. A bundle that is as revealed preferred as another bundle, as shown by choice indifference, is graphically represented as a two-dimensional indifference point (IP). The two red dots in Fig. 1a are IPs relative to the black dot, and relative to each other.
9. Several IPs align as an indifference curve (IC) on which each bundle is as revealed preferred as any other bundle on that same IC, despite different physical bundle composition. The black curve in Fig. 1a is an IC. Bundles on higher ICs (farther from origin) are revealed preferred to bundles on lower ICs, and all bundles on a given IC are revealed preferred to bundles below that IC. Bundles on the red IC in Fig. 1b are revealed preferred to bundles on the blue IC.
10. A neuronal revealed preference signal should be indistinguishable between equally revealed preferred bundles, and thus between all bundles on the same IC (along blue and red ICs in Fig. 1b), but should increase monotonically, or decrease monotonically with inverse coding, with the utility of the bundle across increasing ICs (from blue to red ICs). Although such revealed preference-related changes vary monotonically with choice probability, they need not necessarily correlate numerically with probability. In this study, the terms revealed preference neuron and revealed preference response refer specifically to Revealed Preference Theory, although revealed preferences can be elicited in any observable choice; use of this term does not necessarily imply that the responses carry a numeric code for preference as defined by choice probability.
11. Variations in the composition of equally revealed preferred bundles on the same IC illustrate the graded, fractional and continuous trade-off between bundle components: some quantity of one component is given up in order to gain one unit of the other component without a preference change (Fig. 1a). The trade-off, or compensation in utility, determines the asymmetric IC slope and nonlinear IC curvature, thus dissociating revealed preference from physical bundle composition. A neuronal revealed preference signal should follow IC slope and curvature (Fig. 1b).
12. As a control for meaningful revealed preference, a revealed preferred bundle must remain revealed preferred even when one of its components is smaller than in the alternative bundle (requiring overcompensation by the other bundle component). A neuronal revealed preference signal should satisfy this test (Fig. 1b; blue vs. red stars).

The stochastic choices in our monkeys were meaningful, as shown by the systematic trade-off, well-ordered ICs, transitivity despite one smaller bundle component, and compliance with Arrow’s WARP^27^. Thus, the behavior elicited with our design seemed to follow the IC scheme of Revealed Preference Theory.

The symmetric design with two-component bundles and two-dimensional ICs specified the minimum conditions for studying revealed preference for multi-component choice options. Continuous variation of both reward components allowed us to test the graded, fractional, and continuous component trade-off within choice options, as a change in one component can compensate for a change in the other component. Such a trade-off reflects the emergence of scalar measures, such as stochastic preferences or neuronal responses, from vectorial bundles and is characterized by ICs with IPs away from the axes, and towards the center, of the two-dimensional plot (Fig. 1a, b; Supplementary Fig. 2)^27^. By contrast, standard psychophysical assessments of choice indifference compare one reward with specific quantity in one choice option with its alternative in the other choice option^16,20^. Put graphically, these tests assess IPs only at the axes (or on specific points, depending on scale points) of a two-dimensional graph and do not allow to construct ICs from bundles in between (Supplementary Fig. 26b).

### General behavior

The animals were habituated during several months to enter and then sit relaxed in a primate chair (Crist Instruments) for a few hours each working day. They were trained in specific, computer-controlled behavioral tasks in which they contacted visual stimuli on a horizontally mounted touch-sensitive computer monitor (Elo) in front of them. Following task training for 4 to 6 months, animals were surgically implanted with a recording chamber for electrophysiological recordings, which typically lasted for another 6–10 months. The animals performed the behavioral task during most working days of the week. Each daily task training session lasted for 1 to 2 h; each daily electrophysiological recording session lasted for 1.5 to 3 h. The animals were exposed to a constant, full reward range.

During the experimental sessions, a single animal sat in the primate chair 30 cm away from the computer touch monitor. Its eye positions in the horizontal and vertical plane were monitored with a noninvasive infrared oculometer (Iscan). MATLAB software (Mathworks) running on a Microsoft Windows XP computer controlled the behavior and collected, analyzed, and presented data online. A solenoid valve (ASCO, SCB262C068) controlled by the same Windows computer served to deliver specific quantities of liquids. A Microsoft SQL Server 2008 Database served for Matlab offline data analysis.

### Visual stimuli and reward bundles

The computer-touch monitor presented the animal with two visual stimuli at the left and right (angle of 4°) representing two bundles, respectively (Fig. 1c; Supplementary Fig. 1). Each stimulus indicated a bundle that contained the same two distinct liquid rewards with independently set quantities (reward A, plotted along the *y*-axis of a 2D graph, and reward B, plotted along the *x*-axis). The stimuli were also the touch targets in the task; they appeared at equal reaching distance to the animal, which performed one arm movement to touch the stimulus of the chosen bundle; thus, each bundle was equally affordable to the animal. Each reward was indicated by a distinctly colored stimulus with a superimposed rectangle containing a value bar whose vertical position indicated the quantity of that reward (higher bar = farther away from the animal = more liquid). The notion of higher being better constitutes a valid metaphor across species^49^ (Fig. 1c). In the standard option set, two bundles appeared at the fixed, pseudorandomly alternating left and right positions on the computer monitor; each bundle contained two stimuli. By contrast, the option set for testing Arrow’s Weak Axiom of Revealed Preference (WARP)^36^ consisted of three bundles presented side by side, each with two stimuli. The symmetry in number, presentation and type of reward between the two bundles served to reduce confounds. In both bundles, reward A (top, violet) was always blackcurrant juice, whereas reward B (bottom, green) could be any of four rewards, namely grape juice, water, apple squash or mango juice. A fifth bundle type contained monosodium glutamate (MSG) added to blackcurrant juice (reward A) and inositol monophosphate (IMP) added to grape juice (reward B); this bundle composition was used to test synergistic, enhancing effects of the combination of MSG with IMP. Occasional tests used other combinations of these rewards.

### Behavioral task

After the animal’s hand had contacted a resting-touch stimulus on the computer monitor, the two visual bundle stimuli (or three-bundle stimuli for WARP) appeared on the monitor (Supplementary Fig. 1). Left and right positions of the bundle stimuli alternated pseudorandomly, but there was no further distinction between the stimuli that could serve for identifying them as distinct objects. The animal held the touch key for 0–2 s (0–3 s for three bundles), and then a Go signal appeared (blue dots underneath the bundles). Without any further imposed delay, the animal released the touch key after an average reaction time of 445 ms (Monkey A: 396 ms, Monkey B: 493 ms) and touched the blue Go spot underneath the bundle of its choice. This choice revealed the animal’s preference at this moment. The animal kept touching the Go spot for 1 s, after which it received the reward quantities of the chosen bundle, consisting first of reward A, always followed 500 ms later by reward B. By always delivering the reward components in the same sequence, and by always using blackcurrant juice as reward A, we incurred a consistent discount of reward B that contributed a constant, non-varying, constituent factor to the subjective value (utility) of that reward. No attempt was made to separate this temporal discounting-derived factor from other factors of the bundle reward. The delay, rather than simultaneous delivery of both rewards, was introduced to reduce possible interactions between the different rewards of the bundles.

### Estimation of behavioral ICs

All bundles along a single IC are equally revealed preferred, and thus constitute IPs, and bundles on ICs further away from the origin are revealed preferred over those closer to the origin. Our design tested the graded, fractional and continuous trade-off at equal revealed preference between a gain in one bundle component and a loss in the other bundle component. The trade-off is a defining feature of Revealed Preference Theory and implemented as Marginal Rate of Substitution (MRS), defined as the quantity of one component that is given up in order to obtain one additional unit of the other component at choice indifference between the two bundles (equal revealed preference for each bundle). Formally, MRS is the negative first derivative of the IC slope (MRS = −d*y*/d*x*). In Fig. 1a, the MRS is initially 3:1 and becomes 1:1 with increasing reward B. An non-1:1 trade-off (nondiagonal slope) indicates different subjective value for identical reward amounts, which can be estimated on a common-currency scale of one reference reward (“numeraire”). Thus, MRS, slope and currency define equally well the trade-off.

The behavioral method used to obtain a choice IP has been presented in full detail^27^. With two bundle options, the animal chose between the preset Reference Bundle (left in Fig. 1c) and the Variable Bundle (right) in multiple trials. Thus, the constant Reference Bundle provided a stable reference against the changing bundle composition in the Variable Bundle. We set one reward in the Variable Bundle to one unit (≥0.1 ml) above the quantity of the same reward in the Reference Bundle, while pseudorandomly varying the quantity of the other reward widely. The variation of the animal’s repeated choice with that single varying reward allowed us to construct a full psychophysical function and estimate the IP from a Weibull fit (point of subjective equivalence; *P* = 0.5 choice of each bundle; Fig. 1d). We obtained each IP from a total of 80 trials (two left–right stimulus positions with five equally spaced reward quantities in eight trials). To avoid known adaptations in OFC neurons^15,46,50^, we always tested the full reward range of the experiment.

To obtain an IC, we fit a series of IPs with several different functions using weighted least mean squares. These functions included a linear (first-degree) polynomial (*y* = *ax* + *b*), a quadratic (second-degree) polynomial (*y* = *ax*^2^ + *bx* + *c*) and a hyperbolic function (*d* = *ax* + *by* + *cxy*). Polynomials and hyperbolas provided the equally best fits to the behavioral data (adjusted *R*^2^s of 0.80–0.97)^27^. As the neuronal data were fit somewhat better by a hyperbola (regression with interaction) than a quadratic polynomial, we used only the hyperbolic function

1 $$d = ax + by + cxy$$

with *x* and *y* as reward quantities, a as slope (currency), c as curvature. The hyperbolic function provided the slope (coefficient *b*) and curvature (coefficient *a*) of the behavioral IC. The hyperbolic function can be written in an equivalent form to the regression with interaction used for analyzing neuronal responses (β_0_ = β_1_A + β_2_B + β_3_AB; see Eq. (7) below). Supplementary Fig. 2 shows hyperbolic IC fits to empirically estimated behavioral IPs for all bundles used for neuronal recordings.

### Control regressions for behavioral choice

To test whether the animal’s choice reflected the quantity of the bundle rewards, rather than other, unintended variables such as spatial bias, we used the logistic regression

2 $$P\left({\mathrm{V}} \right) = {\mathrm{\beta }}_0 + {\mathrm{\beta }}_1{\mathrm{CT}} + {\mathrm{\beta }}_2{\mathrm{RA}} + {\mathrm{\beta }}_3{\mathrm{RB}} + {\mathrm{\beta }}_4{\mathrm{VA}} + {\mathrm{\beta }}_5{\mathrm{VB}} + {\mathrm{\beta }}_6{\mathrm{CL}} + {\mathrm{\beta }}_7{\mathrm{RefL}} + {\mathrm{\beta }}{\mathrm{8VT}} - 1 + {\mathrm{\varepsilon }}$$

with *P* (V) as probability of choice of Variable Bundle, β_0_ as offset coefficient, β_1_–β_7_ as strength (slope) coefficients indicating the influence of the respective regressor, CT as trial number within block of consecutive trials, RA as quantity of reward A of Reference Bundle, RB as quantity of reward B of Reference Bundle, VA as quantity of reward A of Variable Bundle, VB as quantity of reward B of Variable Bundle, CL as choice of any bundle stimulus presented at the left, RefL as Reference Bundle stimulus shown at the left, VT-1 as choice of Variable Bundle in previous trial, and ε as error. We used a binomial fit with logit link function to obtain standardized β-coefficients. The results from this regression suggested that the animals based their choice on the quantities of the combined bundle rewards, rather than on the identity of the bundles, both in choice over zero-reward bundles and in choice between two non-zero bundles (Supplementary Fig. 3a, b).

However, the animal might have based its choice on spatial parameters, rather than reward quantities. We assessed this possibility with the logistic regression

3 $$P\left( {\mathrm{L}} \right) = {\mathrm{\beta }}_0 + {\mathrm{\beta }}_1{\mathrm{CT}} + {\mathrm{\beta }}_2{\mathrm{LA}} + {\mathrm{\beta }}_3{\mathrm{LB}} + {\mathrm{\beta }}_4{\mathrm{RiA}} + {\mathrm{\beta }}_5{\mathrm{RiB}} + {\mathrm{\beta }}_6{\mathrm{RefL}} + {\mathrm{\beta }}_7{\mathrm{LT}} - 1 + {\mathrm{\varepsilon}}$$

with *P* (L) as probability of left choice, CT as trial number within block of consecutive trials, LA as quantity of any bundle reward A whose stimulus was presented at the left on the computer monitor, LB as quantity of bundle reward B presented at left, RiA as quantity of bundle reward A presented at right, RiB as quantity of bundle reward B presented at right, RefL as Reference Bundle stimulus presented at left, and LT-1 as left choice in previous trial. The results from this regression suggested that the animals based their choice on the quantities of the combined bundle rewards, rather than on spatial variables, both in choice over zero-reward bundles and in choice between two non-zero bundles (Supplementary Fig. 3c, d).

### Surgical procedures and electrophysiology

A head-restraining device and a recording chamber (40 × 40 mm, Gray Matter) were implanted on the skull under full general anesthesia and aseptic conditions. The stereotactic coordinates of the chamber enabled neuronal recordings of the orbitofrontal cortex (OFC)^51^. We located the OFC from bone marks on coronal and sagittal radiographs taken with a guide cannula inserted at a known coordinate in reference to the implanted chamber, using a medio-lateral vertical and a 20° degree forward directed approach (Supplementary Fig. 6). Monkey A provided data from the left hemisphere, Monkey B from the right hemisphere, via a craniotomy in each animal ranging from Anterior 30 to 38, and Lateral 0 to 19. We conducted single-neuron electrophysiological recordings using both custom made glass-coated tungsten electrodes^52^ and commercial electrodes (Alpha Omega, Israel) (impedance of ~1 MOhm at 1 KHz). Electrodes were inserted into the cortex with a multi-electrode drive (NaN drive, Israel) with the same angled approach as used for the radiography. Neuronal signals were collected at 20 kHz, amplified using conventional differential amplifiers (CED 1902 Cambridge Electronics Design), and band-passed filtered (high: 300 Hz, low: 5 kHz). We used a Schmitt-trigger to digitize the analog neuronal signal online into a computer-compatible TTL signal. However, we did not use the Schmitt-trigger to separate simultaneous recordings from multiple neurons, in which case we searched for another single-neuron recording or occasionally stored the data in analog form for offline separation by dedicated software (Plexon offline sorter). An infrared eye tracking system monitored eye position (ETL200; ISCAN).

### Trial types for neuronal tests

We conducted electrophysiological recordings during choices between bundles positioned on specific IPs of behavioral ICs. We tested at least ten trials per bundle during two-option choices (between two bundles containing the same two rewards in varying quantity) for general data collection, and three-option choices (between three bundles) for testing Arrow’s WARP, using the same experimental designs as our behavioral study^27^.

With two-option choice sets, we tested two trial types:

- Zero-reward bundle trials: both rewards of the Reference Bundle were set to zero quantity, whereas the Variable Bundle contained at least one non-zero quantity. As the zero-reward bundle was constant, only the explicitly varied non-zero bundle should affect the animal’s choice. Indeed, given their extensive training, the animals unfailingly chose the varying nonzero bundle. This version-related neuronal responses primarily to variations of the nonzero option and aided in their interpretation. These trials were also helpful for keeping the animals attentive and focused on the task. Trial blocks with zero-reward bundles consisted usually of five equally spaced quantities of both rewards of the nonzero bundle.
- Choice trials: one or both rewards of both bundles were set to the quantities of any IP on any IC, and the animal chose the bundle of its preference. These trials provided the core data of the study. Blocks of choice trials consisted of 24 trial types that were repeated ten or more times depending on neuronal recording stability (12 options, 2 left–right pseudorandomly alternating stimulus positions). A subform of choice trials consisted of trials with degenerate bundles that anchored them to the two coordinate axes of the indifference map; the nonzero reward of one bundle was set to a specific quantity on one axis, and the quantity of the other nonzero reward of the alternative bundle was set to a specific quantity on the other axis. These anchoring trials served (i) to establish the basic neuronal relationship to the quantity of individual rewards, (ii) to provide behavioral control measures for motivation and reward-specific satiety^27^, and (iii) to allow regression analysis of chosen value coding using the value of any bundle reward relative to the common currency of blackcurrant juice or blackcurrant–MSG (which served as reference, numeraire) (Eqs (11, 12)). A block of anchor trials consisted typically of 80 trials. The data were excluded whenever a behavioral change in common-currency value was detected (Supplementary Fig. 5).

As some neurons in orbitofrontal cortex (OFC) are known to adapt to the distribution of reward quantity^27,46,50^, we interspersed ~20% zero-reward bundle and anchor trials between full choice trials in order to keep the quantity distribution constant.

When testing WARP, the animal chose between three simultaneously presented bundles. In two of these bundles, one reward of one bundle and the other reward of the other bundle were set to zero quantity, thus anchoring the bundles to the two respective axes of the indifference map. The third bundle contained variable quantities of both rewards, thus being located on an IC away from the axes. The two anchor bundles were set on the same IC, whereas the third bundle was set either on that same IC or on an IC above or below that of the two anchor bundles.

### Assessment of basic task relationships

We measured electrophysiological activity from 694 single OFC neurons during task performance. Tested bundles alternated pseudorandomly; data were post hoc ordered for presentation. Unless otherwise stated, we analyzed neuronal impulses during four task epochs vs. Pretrial control (1 s): visual Bundle stimulus (2 s), Go signal (1 s), Choice (1 s) and Reward (2 s, starting with reward A, followed 0.5 s later by reward B, thus covering both rewards).

We used the paired Wilcoxon test (*P* < 0.01) for comparing the activity in each neuron during each task epoch separately against the Pretrial control epoch. A neuron was considered task-related if its activity in at least one of the four task epochs differed significantly from the activity during the Pretrial control epoch. This Wilcoxon analysis yielded one or more statistically significant task-related responses in 441 OFC neurons out of the 694 tested OFC neurons.

To confirm the Wilcoxon-identified task relationships with a different statistical test (method-independent assessment), we used a one-factor ANOVA with 150 -ms sliding window (bin-width of 50 ms) and required four consecutive windows (total of 600 ms) to define significance against the Pretrial control epoch (*P* < 0.01). The ANOVA analysis yielded task-related responses in 493 OFC neurons out of the 694 tested OFC neurons. All Wilcoxon-significant neurons were also ANOVA significant. Thus, the ANOVA analysis confirmed the results from the fixed-window Wilcoxon analysis. For reasons of conservative statistics, all further analyses focussed on the task-related responses in the 441 Wilcoxon-significant task-related neurons.

Of the 441 neurons with task-related responses identified by the Wilcoxon test, 325 neurons were tested in binary, two-option choice over a zero-reward bundle, 391 neurons (including the 325 neurons) were tested in binary, two-option choice between two non-zero bundles, and 56 neurons were tested in trinary, three-bundle choice for WARP (including six neurons submitted to all three tests).

### Definition of neuronal revealed preference coding

Revealed preference responses of single neurons should follow the typical scheme of behavioral ICs described by Revealed Preference Theory (Fig. 1b), which requires three crucial characteristics:

Characteristic 1: Following the monotonicity assumption on preferences^8^, activity should change monotonically across behavioral ICs with increasing behavioral revealed preference, even when a revealed preferred bundle contains one smaller reward component than the alternative bundle. Such monotonic neuronal response changes reflect increasing quantities of one or both bundle rewards, assuming a positive monotonic subjective value function on reward quantity; the current study did not test bundles and situations that were possibly not associated with positive monotonic value functions, such as negatively valued rewards, post-satiety rewards, or punishers. Importantly, a monotonic activity change (increase or decrease) defines the sensitivity to revealed preference of the neuron under study. Without demonstrating such sensitivity, the next analysis would be meaningless.

Characteristic 2: All bundles along a same-revealed-preference IC should elicit the same, insignificantly varying neuronal response. Their activity should reflect the systematic trade-off between the two bundle rewards while maintaining the same revealed preference. This crucial characteristic requires the same neuronal responses to bundles that are equally revealed preferred, despite different physical bundle composition. Thus, neuronal responses should differ insignificantly, despite different quantities of the two bundle rewards as long as the bundles were equally revealed preferred (i.e., positioned on the same behavioral IC). By contrast, responses varying significantly along individual ICs would not code the crucial graded trade-off, and thus not follow the formalism of Revealed Preference Theory.

Characteristic 3: The ICs are often not symmetric (diagonal) and linear, and the neuronal response should match these behavioral parameters. The IC slope reflects the currency relationship between the two bundle rewards, indicating the revealed preference relation between the two rewards of a bundle, and thus the value of one reward relative to a common-currency reference reward (numeraire). We estimated slope and curvature parameters from regression coefficients of each neuronal response (see below) and compared them with the average slope and curvature of all behavioral ICs of a given bundle type.

To summarize, to suggest multi-component bundle coding compatible with the graphic formalism of Revealed Preference Theory, a given neuronal response must necessarily show revealed preference sensitivity (characteristic 1), reflect the systematic trade-off at equal revealed preference by insignificantly varying with different bundles positioned on the same IC (characteristic 2), and have similar IC slope and curvature as the behavioral IC of the tested bundle type (characteristic 3).

In contrast to these responses concerning combinations of two bundle rewards, single-reward coding responses would vary only with a single bundle reward. Some OFC neurons are known to code only single reward (offer value coding^16^); their existence was confirmed in our study and provided a contrast to revealed preference coding (note that single-reward lexicographic preferences were not observed in the behavioral choices in our study^27^).

### Statistical analysis of neuronal revealed preference coding

We used the three characteristics mentioned above to identify a neuronal signal compatible with the graphic formalism of Revealed Preference Theory. We used a conservative statistical approach with minimal assumptions about the nature of the potential neuronal code. We assumed only positive or negative monotonic relationships with reward quantity and ordinal (rank-ordered) relationships to revealed preference (more is better, without indicating by how much); we did not assume linear numeric relationships. For comparison with other neurophysiological studies, we used the most basic, best-understood statistical tools for analyzing neuronal data. To this end, we employed a combination of three statistical tests that reflected the three characteristics, comprising (1) conventional linear regressions to assess the monotonicity of neuronal response change across ICs in approximation to linearity, but without formally assuming it (*P* < 0.05 for β-coefficients; *t* test) (see below, Eq. (4)), (2) Spearman rank-correlation to confirm ordinal monotonicity of response change across ICs without assuming a particular numeric scale (*P* < 0.05), and (3) two-factor ANOVA to assess significant response change in the spirit of ICs: significance across ICs and insignificance within ICs, without regard of monotonicity of change (*P* < 0.05 for factors). An insignificant change within ICs in the ANOVA is not necessarily equal to an absence of change; however, significance across ICs would suggest sufficient test sensitivity to indicate that insignificance within ICs was not due to test insensitivity or insufficient data. Therefore, we considered an absence of neuronal response change along an IC in the corresponding ANOVA factor as meaningful and indicative of response similarity. We used the two-factor ANOVA instead of a double-linear regression for this purpose, because an absence of significance in a regression indicates insignificant monotonic change while not ruling out non-monotonic changes; an ANOVA would be sensitive also to non-monotonic changes, and an absence of significance in an ANOVA is therefore closer to absence of change. To indicate coding of revealed preference, a neuronal response had to pass all three tests. Due to the conservative nature of these tests, our analysis may have underestimated the incidence and importance of revealed preference signals in OFC. Note that we also obtained population codes for revealed preference from combining single neuron responses not passing all three tests (see below).

To assess the described characteristics, we used the three statistical tests on the 441 Wilcoxon-identified task-related neurons as follows:

Characteristic 1: To capture the scalar neuronal activity representing the vectorial bundle (*y* = f (reward A, reward B)) in the most conservative, assumption-free manner possible, we tested neuronal sensitivity to both bundle components as necessary requirement for revealed preference coding, using the linear regression:

4 $$y = {\mathrm{\beta }}_0 + {\mathrm{\beta }}_1{\mathrm{A}} + {\mathrm{\beta }}_2{\mathrm{B}} + {\mathrm{\varepsilon }}$$

with *y* as neuronal response in any of the four task epochs, measured as impulses/s and z-scored normalized to the Pretrial control epoch of 1.0 s (z-scoring of neuronal responses applied to all regressions listed below), A and B as milliliter quantity of reward A (plotted at *y*-axis on 2D indifference map, Fig. 1b) and reward B (plotted at *x*-axis), respectively, β_0_ as offset coefficient, β_1_ and β_2_ as neuronal coding slope coefficients, and ε as error consisting of the sum of individual errors of each expression (err_0_, err_1_, err_2_ for offset and respective regressors 1 and 2). The coefficients β_1_ and β_2_ needed to be either both positive (indicating positive neuronal relationship, higher neuronal activity reflecting more reward quantity) or both negative (inverse neuronal relationship) to reflect the additive nature of the individual bundle components giving rise to revealed preference (*P* < 0.05, unless otherwise stated; *t* test). Equation (4) together with same-signed βs (either both βs positive or both βs negative) constituted our basic screening statistics that defined the number of neurons potentially coding revealed preference.

By contrast, we tested the coding of single rewards A or B with the reduced regressions:

5 $$y = {\mathrm{{\beta}}}_0 + {\mathrm{{\beta }}}_1{\mathrm{A}} + {\mathrm{\varepsilon }}$$

and

6 $$y = {\mathrm{\beta }}_0 + {\mathrm{\beta }}_1{\mathrm{B}} + {\mathrm{\varepsilon }}$$

with ε = err_0_ + err_1_. To demonstrate single-reward coding, we required significance in (1) Eqs (5) and (6) for only reward A or only reward B, respectively, but not in both Eqs. (2) Eq. (4) for only the A or only the B reward but not both regressors, and (3) the F-test between Eqs (4) and (5) (identifying coding of reward B) and between Eqs (4) and (6) (identifying coding of reward A).

Characteristic 1: All neurons with significant positive or negative changes identified by Eq. (4) needed to be also significant in the Spearman rank-correlation test.

Characteristics 1 and 2: To assess together the first two necessary conditions for revealed preference coding in a direct and intuitive way, we used a two-factor ANOVA on each Wilcoxon-identified task-related response that was significant for both regressors in Eq. (4); the factors were across-IC (ascending rank order of behavioral ICs) and within-IC (same rank order of behavioral IC). To be a candidate for coding revealed preference, changes across ICs should be significant (*P* < 0.05), changes within-IC should be insignificant, and their interaction should be insignificant.

Characteristic 3: Whereas the regression defined by Eq. (4) would provide a conservative estimate of revealed preference coding, the full construction of neuronal ICs for comparison with behavioral ICs requires inclusion of the IC curvature that depends on both rewards. To this end, we extended Eq. (4) by adding the interaction term AB:

7 $$y = {\mathrm{\beta }}_0 + {\mathrm{\beta }}_1{\mathrm{A}} + {\mathrm{\beta }}_2{\mathrm{B}} + {\mathrm{\beta }}_3{\mathrm{AB}} + {\mathrm{\varepsilon }}$$

with ε = err_0_ + err_1_ + err_2_ + err_3_. The neuronal IC slope was estimated from the ratio of coefficients β_2_/β_1_. Note the different meanings of the slope term: the neuronal IC slope (β_2_/β_1_) describes the relative coding strength of the two bundle rewards (reflecting the currency of the two rewards), whereas the neuronal coding slope alone (β) describes the strength of neuronal response. The neuronal IC curvature was the β_3-_coefficient of the interaction term AB (all β‘s *P* < 0.05; *t* test). The regression defined by Eq. (7) is identical to the hyperbolic model used for fitting behavioral ICs (d = ax + by + cxy).

To assess numerically the match in slope and curvature parameters between neuronal responses and behavioral ICs, we searched for an overlap between the means of the neuronal IC slope and curvature parameters and the ±95% confidence interval of the distribution of the behavioral IC slope and curvature parameters, and vice versa. Although these parameters combine into the local slope (negative of Marginal Rate of Substitution, MRS), they may differ in opposite directions and, by mutually canceling their difference, might falsely indicate an MRS match; therefore, we performed separate comparisons for slope and curvature.

To compare graphically the neuronal responses in any of the four task epochs with the behavioral ICs, we plotted two-dimensional neuronal ICs along which all neuronal responses were equal. As per definition a response *y* would be constant along a whole neuronal IC, we merged *y* with the constants offset (β_0_) and error (ε) into a common final constant *k*:

7a $$k = {\mathrm{\beta }}_1{\mathrm{A}} + {\mathrm{\beta }}_2{\mathrm{B}} + {\mathrm{\beta }}_3{\mathrm{AB}}$$

To draw the neuronal IC, we computed the quantity of component A as a function of component B from the derived equation:

7b $$A = k - ({\mathrm{\beta }}_2/{\mathrm{\beta }}_1){\mathrm{B}} + {\mathrm{\beta }}_3{\mathrm{B}}$$

To obtain a two-dimensional map of neuronal ICs, we plotted the preset quantity of component B on the *x*-axis and the quantity of component A computed from Eq. (7b) on the *y*-axis. For details, see below (Population plots).

Further regressions served as controls for the validity of our basic analysis (Eqs (4) and (7)), such as the quadratic model:

8 $$y = {\mathrm{\beta }}_0 + {\mathrm{\beta }}_1{\mathrm{A}} + {\mathrm{\beta }}_2{\mathrm{B}} + {\mathrm{\beta }}_3{\mathrm{A}}^2 + {\mathrm{\beta }}_4{\mathrm{B}}^2 + {\mathrm{\varepsilon }}$$

with ε = err_0_ + err_1_ + err_2_ + err_3_ + err_4_. We also used a higher-order interaction model:

9 $$y = {\mathrm{\beta }}_0 + {\mathrm{\beta }}_1{\mathrm{A}} + {\mathrm{\beta }}_2{\mathrm{B}} + {\mathrm{\beta }}_3{\mathrm{A}}^2 + {\mathrm{\beta }}_4{\mathrm{B}}^2 + {\mathrm{\beta }}_5{\mathrm{AB}} + {\mathrm{\varepsilon }}$$

with ε = err_0_ + err_1_ + err_2_ + err_3_ + err_4_ + err_5_.

However, we based our neuronal analysis on the most conservative model involving Eq. (4), as it avoided up to 5% false positives due to its fewer regressors and thus allowed us to be as conservative as possible to reduce the number of assumptions about this previously unreported neuronal signal.

For a more comprehensive assessment, we incorporated all individual bundle components, together with additional variables, into a single regression:

10 $${{y}} =\,\, 	 {\mathrm{\beta }}_0 + {\mathrm{\beta }}_1{\mathrm{CT}} + {\mathrm{\beta }}_2{\mathrm{RA}} + {\mathrm{\beta }}_3{\mathrm{RB}} + {\mathrm{\beta }}_4{\mathrm{VA}} + {\mathrm{\beta }}_5{\mathrm{VB}} + {\mathrm{\beta }}_6{\mathrm{CV}} \\ 	\quad+ {\mathrm{\beta }}_7{\mathrm{VT}} - 1 + {\mathrm{\beta }}_8{\mathrm{CL}} + {\mathrm{\varepsilon }}$$

with *y* as neuronal response, β_0_ as offset coefficient, β_1_–β_8_ as neuronal coding slope coefficients, CT as trial number within block of consecutive trials, RA as reward A in Reference Bundle, RB as reward B in Reference Bundle, VA as reward A in Variable Bundle, VB as reward B in Variable Bundle, CVB as choice of Variable Bundle in current trial, VT-1 as choice of Variable Bundle in previous trial, and CL as choice of any bundle stimulus presented on the left.

### Polar plot of neuronal reward sensitivity

The purpose of this analysis was to graphically display the general sensitivity of OFC neurons to single or multiple rewards and align the presentation with data from previous studies^15,16^. This analysis provided quantitative information about one of the basic requirements of revealed preference coding, namely the monotonic increase or decrease of activity with increasing quantity of both bundle rewards across ICs (characteristic 1 above), but without addressing trade-off, slope, and curvature (characteristics 2 and 3). The 2D polar plot of Supplementary Fig. 11a shows such monotonic changes and indicates the relative coding strength of each of the two bundle rewards based on the neuronal coding slope (β-coefficients in linear regression Eq. (4)); further characteristics of revealed preference such as systematic trade-off across multiple IPs and IC curvature played no role in these graphs. The alignment of the colored dots along the diagonal axis reflects the relative strength of the β-coefficients for each bundle reward (A+ B+ and A−B− responses, defined by significance of both β-coefficients; the sign indicates positive or inverse monotonic coding). A deviation of the alignment angle from the diagonal line indicates a non-1:1 currency relationship between the two bundle rewards (for definition of common currency, see below). The polar plot of Supplementary Fig. 11a shows also data from neuronal responses that coded only one of the bundle rewards (A+, B+, A−, B− responses). For these neurons, the β-coefficients from the regression analysis with Eq. (4) were significant for only one reward and aligned toward the vertical and horizontal axes of the 2D plots. These categories occurred with all tested bundle combinations (Tables 1a and 3a) and in all four task epochs (Supplementary Figs. 11b–f, 12). These plots display the monotonic quantitative coding of individual rewards as necessary (but not sufficient) condition for coding revealed preference among multi-reward bundles. However, the plots did not capture a neuronal code for the trade-off as crucial formalism of Revealed Preference Theory.

### Neuronal chosen value coding

Chosen value is defined as the value of a choice option the animal considers, would obtain or has obtained by its choice. In analogy, unchosen value refers to the value of the option not chosen. As each option consisted of two components, we defined the chosen value (CV) of each option in two ways. The conservative approach made minimal assumptions and used a linear combination of the quantity of the two-component rewards A (blackcurrant juice) and B (any of the other five rewards):

11 $${\mathrm{CV}} = {\mathrm{A}} + {\mathrm{k}}_1{\mathrm{B}}$$

A more sophisticated approach accounted for IC nonlinearity by adding the interaction term AB:

11a $${\mathrm{CV}} = {\mathrm{A}} + {k}_1{\mathrm{B}} + {\mathrm{k}}_2{\mathrm{AB}}$$

In analogy, we defined unchosen value (UCV) by linear combination as:

12 $${\mathrm{UCV}} = {\mathrm{A}} + {{k}}_1{\mathrm{B}}$$

and with interaction term AB as:

12a $${\mathrm{UCV}} = {\mathrm{A}} + {{k}}_1{\mathrm{B}} + {{k}}_2{\mathrm{AB}}$$

Weighting parameter *k*_1_ served to adjust for differences in subjective value between rewards A and B, such that the quantity of reward B enters the regression on a common-currency scale defined by reward A. Weighting parameter *k*_2_ is identical to coefficient β_3_ of Eq. (7) and served to account for interaction between the two bundles to reflect IC nonlinearity.

In detail, we established parameter k_1_ during neuronal recording sessions from behavioral choice IPs using quantitative psychophysics in anchor trials (80 trials per test, see above Trial types for neuronal tests), rather than reading it from fitted ICs. Thus, *k*_1_ equals the ratio of coefficients β_2_/β_1_ (see Eq. (7)). We established a common-currency scale in ml for all tested rewards by defining blackcurrant juice or blackcurrant–MSG (reward A) as reference (numeraire); the subjective value of any reward is expressed as real-number multiple k_1_ of the quantity of the numeraire at choice indifference.

Specifically, the animal chose between the Variable Bundle that contained a psychophysically varied quantity of blackcurrant juice (the other bundle reward being set to 0 ml) and the Reference Bundle that contained a fixed quantity of the other reward (blackcurrant juice being set to 0 ml). At choice indifference, the quantity of blackcurrant juice (reward A) in the Variable Bundle defined the common-currency value of the other reward, from which we calculated parameter *k*_1_ as A/B. A *k*_1_ of <1 indicates that more quantity is required for choice indifference against blackcurrant juice, and that *k*_1_ brings down this higher quantity to a level comparable with that of reward A; thus, a *k*_1_ < 1 suggests that the tested reward has lower subjective value than blackcurrant juice. By contrast, a *k*_1_ > 1 suggests higher subjective value, as less quantity is required for choice indifference, and that *k*_1_ elevates this lower quantity to the level of reward A. An example result is seen on the highest IC in Supplementary Fig. 2b; the quantity of 0.8 ml of water (reward B: *y* = 0, *x* = 0.8 ml) was positioned on the same IC established from psychophysically assessed IPs, and thus was subjectively equally valuable to the animal, as 0.5 ml of blackcurrant juice (reward A: *y* = 0.5 ml, *x* = 0). In this case, *k*_1_ = A/B = 0.5/0.8 = 0.625. Thus, water was subjectively worth 0.625 of the value of blackcurrant juice; the *k*_1_ of 0.625 put the water indifference quantity of 0.8 ml to a subjectively equivalent value of 0.5 ml for Eqs (11) and (12).

We assessed the coding of chosen value and unchosen value in all neurons coding revealed preference in choices between two nonzero bundles, using the following regression:

13 $$y = {\mathrm{\beta }}_0 + {\mathrm{\beta }}_1{\mathrm{CV}} + {\mathrm{\beta }}_2{\mathrm{UCV}} + {\mathrm{err}}$$

with err as a compound error for all regressors.

We defined the following forms of chosen and unchosen value coding according to the significance of the two β-coefficients (*P* < 0.05; *t* test; Table 3): absolute chosen value refers only to the option the animal is choosing; relative chosen value refers to the difference chosen value minus unchosen value; unchosen value refers only to the option the animal is not choosing; total value refers to the sum chosen value plus unchosen value.

For a more comprehensive assessment, we incorporated additional choice variables:

14 $${{y}} =	 {\,\,}{\mathrm{\beta }}_0 + {\mathrm{\beta }}_1{\mathrm{CT}} + {\mathrm{\beta }}_2{\mathrm{CV}} + {\mathrm{\beta }}_3{\mathrm{UCV}} + {\mathrm{\beta }}_4{\mathrm{CV}} - 1 + {\mathrm{\beta }}_5{\mathrm{UCV}} - 1 + {\mathrm{\beta }}_6{\mathrm{RefL}} \\ 	+ {\mathrm{\beta }}_7{\mathrm{VT}} - 1 + {\mathrm{\beta }}_8{\mathrm{CL}} + {\mathrm{\varepsilon }}$$

with CT as a consecutive trial number, CV as a chosen value, UCV as a value of the unchosen option, CV-1 as a chosen value in previous trial, UCV-1 as an unchosen value in previous trial, RefL as a Reference Bundle stimulus shown on the left, VT-1 as a choice of Variable Bundle in previous trial, and CL as a choice of left bundle. Coefficient CL varied with left choice, as opposed to left position of the reference bundle, RefL, irrespective of choice. Applying regression Eq. (14) without CL resulted in insignificant variation in the other coefficients up to 6.56% ± 2.17 (mean ± SEM).

### Neuronal population plots

We obtained the two-dimensional ICs of neuronal population responses and their numeric analyses shown in Figs. 3 and 6e, f, and Supplementary Figs. 17–19 and 25 from the following steps.

1a) For the population plots of revealed preference responses shown in Fig. 3a–d and Supplementary Figs. 17, 18, and 25, we used the individual revealed preference responses identified by our conservative three-test procedure with the double linear regression (Eq. (4)). Each neuron could have a distinct response in one or more of the four task epochs of Bundle stimulus, Go signal, Choice, and Reward. Thus, the number of responses was expected to be equal to or higher than the number of responding neurons.

1b) For the aggregate population plots shown in Fig. 6e, f and Supplementary Fig. 25, we used the individual single-reward responses identified by Eqs (5) or (6) and confirmed by significance for the same, but not the other reward in the double-linear regression (Eq. (4)) plus significance in the F-test between Eq. (4) and either Eq. (5) or Eq. (6).

2) To obtain an average neuronal impulse count for a given response to a given bundle in any of the four task epochs, we counted the total number of neuronal impulses in the analysis window for the respective task epoch. Then we divided that count by the number of test trials.

3) To obtain a normalized count for each response, we calculated the z-score for each response obtained in step 2 (subtraction from mean neuronal activity during the Pretrial epoch and division by standard deviation of that activity).

4) To obtain a neuronal z-score population count for each bundle, we averaged all z-scored neuronal responses to that bundle (Fig. 3a, b). Thus, neurons with significant changes in >1 task epoch contributed multiple responses to the population average. As subjective revealed preferences are by definition specific to each individual animal, we never averaged neuronal responses across animals (nor their behavioral data).

5a) For the two-dimensional population plots of revealed preference responses shown in Fig. 3a–d and Supplementary Figs. 17 and 18, we regressed all z-scored individual neuronal revealed preference responses, which had been initially identified by the three-test procedure (see point 1a above), using Eqs (7) and (7b) and plotted the neuronal IC with the newly estimated regression coefficients (βs). Thus, each neuronal IC reflected the best regression fit to insignificantly differing responses along that IC. We plotted each neuronal IC from the preset quantity of component B on the *x*-axis and the quantity of component A computed from Eq. (7b) on the *y*-axis. We indicated the z-scored strength of every significant neuronal revealed reference response in any of the four task epochs by coloring the bundle position on the two-dimensional map (Fig. 3a, b and 11g–j). Thus, the *x*–*y* position of each dot corresponded to the reward quantity of the two bundle components (A, B), the color of each dot reflected the response strength (from blue to orange and red), and same-colored dots represented similar response strength. Note that the neuronal responses had initially been identified by our conservative three-test procedure (using Eq. (4) for the regression, see point 1a above).

5b) To validate the populations plots by the out-of-sample tests shown in Fig. 3e, f, we set new bundles not used for modeling neuronal ICs to specific *x*–*y* positions on the two-dimensional map. Then we established the strength of the positive or inverse coding response to each new bundle and indicated it by coloring the bundle position on the two-dimensional map. Then we computed the distance between the position of the new bundle and the neuronal IC that represented the strength of the response closest to that induced by the bundle (same color as bundle position). As metric for Fig. 3e, f, we followed the notion of Eq. (7b) and used the vertical (*y*-axis) distance between bundle position and neuronal IC of matching color (i.e., in units of ml of blackcurrant juice or blackcurrant–MSG juice).

5c) For the aggregate population plots shown in Fig. 6e, f, we regressed all z-scored single-reward coding responses on the quantity of reward A and reward B and plotted the neuronal IC using Eq. (7b) with the regression coefficients (βs) derived from Eqs (5) and (6), respectively, and averaged from single responses.

5d) For the mixed-aggregate population plots shown in Supplementary Fig. 25, we regressed all z-scored revealed preference and single-reward coding responses on the quantity of reward A and reward B and plotted the neuronal IC using Eq. (7b) with the regression coefficients (βs) derived from Eqs (5–7), respectively, and averaged from single responses.

6) To obtain the ±95% neuronal confidence intervals for Fig. 3a, b and Supplementary Figs. 17 and 18, we used the err_0_ term from the offset in the regression (Eq. (7)) and plotted it, in the direction of the *y*-axis, above and below the curve derived from Eq. (7b).

### Neuronal decoders

We used linear support vector machine (SVM) and linear discriminant analysis (LDA) algorithms to decode neuronal activity according to bundles on different behavioral ICs during choice over zero-reward bundle (bundle distinction) and, separately, according to behavioral choice between two nonzero bundles located on different ICs (choice prediction). We implemented both decoders as custom-written software in MATLAB R2015b (Mathworks). The SVM decoder with linear kernel was accomplished with svmtrain and svmclassify procedures (our previous work had shown that use of nonlinear svm kernels does not improve decoding^44^). The SVM decoder was trained to find the optimal linear hyperplane for the best separation between two neuronal populations relative to lower vs. higher ICs. The LDA decoder was obtained with fitcdiscr and predict procedures. The LDA decoder was trained to find the axes (linear discriminants) for best separation between lower vs. higher ICs by maximizing the ratio of between-class variance to within-class variance, what may be described as reducing the data variation in the same class when increasing at the same time the data separation between classes. The first two linear discriminant components were additionally used to graphically represent the separation between lower and higher ICs for selected datasets.

All analyses employed single-neuron data, consisting of single-trial impulse counts that had been z-normalized to the activity during the Pretrial epoch in all trials recorded with the neuron under study. The analysis included activity from all neurons showing revealed preference coding during any of the four task epochs, as identified by our three-test statistics, except where noted. The neurons were recorded one at a time; therefore the analysis concerned aggregated pseudo-populations of neuronal responses.

The decoding analysis used 10 trials per neuron for each of two ICs (total of 20 trials). Extensive analysis suggested that higher inclusion of 15–20 trials per group did not provide significantly better decoding rates (while reducing the number of included neurons). For neurons that had been recorded with >10 trials per IC, we selected randomly 10 trials from each neuron for each of the two ICs. We used a leave-one-out cross-validation method, in which we removed one of the 20 trials and trained the SVM/LDA decoder on the remaining 19 trials. We then used the SVM/LDA decoder to assess whether it accurately detected the IC of the left-out trial. We repeated this procedure 20 times, every time leaving out another one of the 20 trials. These 20 repetitions resulted in a percentage of accurate decoding (% out of *n* *=* 20). The final percentage estimate of accurate decoding resulted from averaging the results from 150 iterations of this 20-trial random selection procedure.

To distinguish from chance decoding, we randomly shuffled the assignment of neuronal responses to the tested ICs, which should result in chance decoding (accuracy of 50% correct). A significant decoding with the real, non-shuffled data would be expressed as statistically significant difference against the shuffled data (*P* < 0.01; Wilcoxon rank-sum test).

For visualization with LDA, we selected randomly ten trials from all neurons that were tested with two bundles located on two ICs (lowest and highest IC). The bundles were selected for a specific analysis, such as choice over zero-reward bundle, choice between two nonzero bundles, revealed preferred bundles with one lower reward quantity than in the alternative bundle, individual task epochs, or any combination thereof. We attributed the first linear discriminant to separation between bundles on two different ICs, and the second linear discriminant to separation of bundles along same ICs. For the two-dimensional graph, we randomly selected five combinations of randomly selected ten trials of neuronal responses to each bundle and superimposed the data (see Fig. 4e, f).

### Reporting summary

Further information on research design is available in the Nature Research Reporting Summary linked to this article.

## Supplementary information

Supplementary Information

Reporting Summary

## Data Availability

The data are available from the authors upon reasonable request. A Reporting Summary for this article is available as a Supplementary Information file.
