# Supplementary material for: Orbitofrontal signals for two-component choice options comply with indifference curves of Revealed Preference Theory
Source: Nat Commun. 2019 Oct 25;10:4885. doi: 10.1038/s41467-019-12792-4 (PMC6814743; doi:10.1038/s41467-019-12792-4)
Supplement: Supplementary file 1 — Supplementary Information [file 41467_2019_12792_MOESM1_ESM.pdf]

Supplementary Information

Integrated and distinct orbitofrontal signals for two-component choice options comply with indifference curves of Revealed Preference Theory

Pastor-Bernier et al.

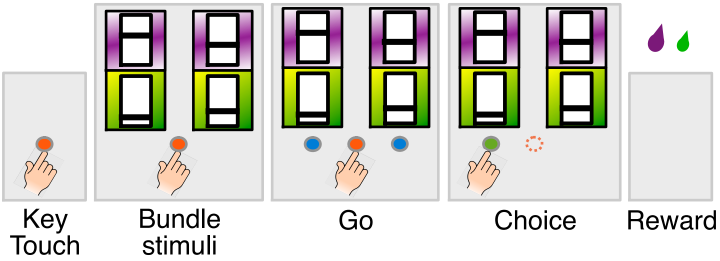

Supplementary Fig. 1. Task sequence. Each choice option consisted of a bundle containing the same two rewards (violet, green) with independently set quantities. Each quantity was indicated by the vertical position of a bar within a rectangle (higher is more).

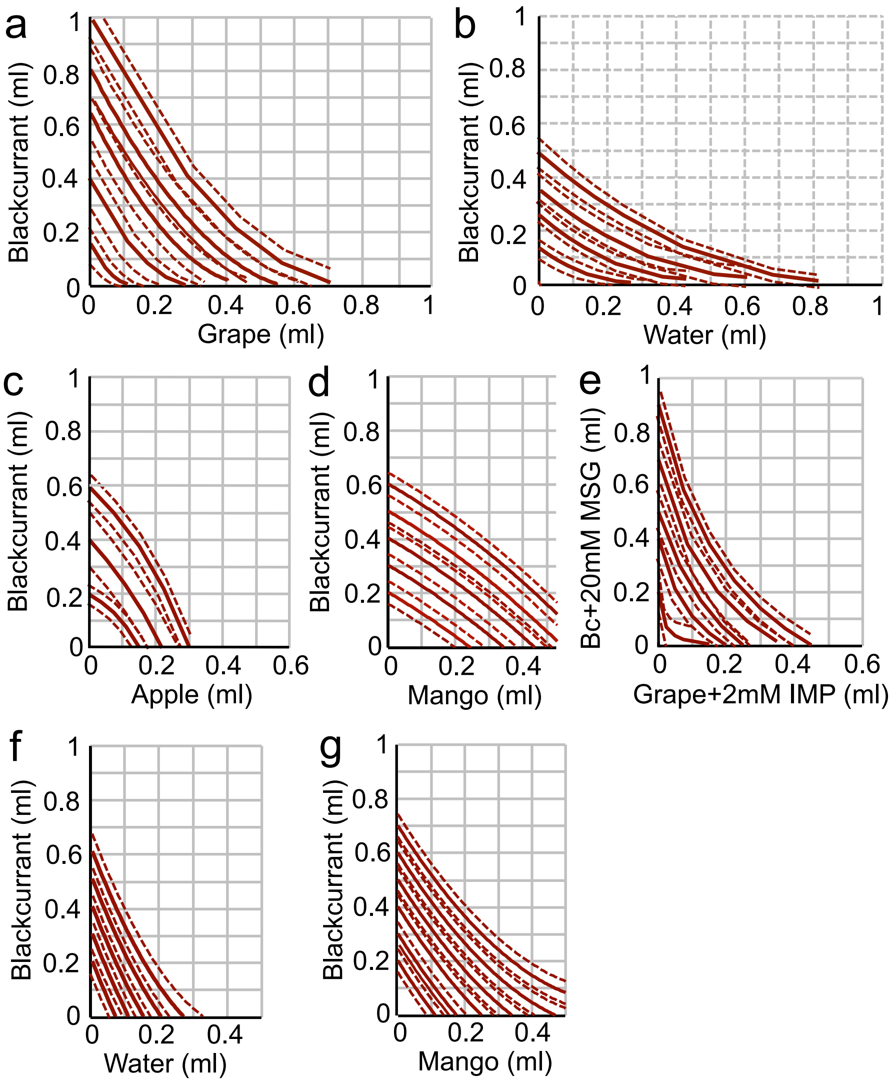

Supplementary Fig. 2. Behavioral indifference curves. **a-e** Behavioral indifference curves (ICs) for all bundle types used in the current experiment with Monkey A. Heavy lines show ICs fitted to indifference points of same color by hyperbolic function (d=ax+by+cxy, Eq. 1; equivalent to regression with interaction, Eq. 7); dotted thin lines show ± 95% confidence intervals for respective ICs. Reward A is plotted on the y-axis, reward B on the x-axis. Bc, blackcurrant juice; MSG, monosodium glutamate; IMP, inosine monophosphate. **f, g** ICs for bundle types used with Monkey B.

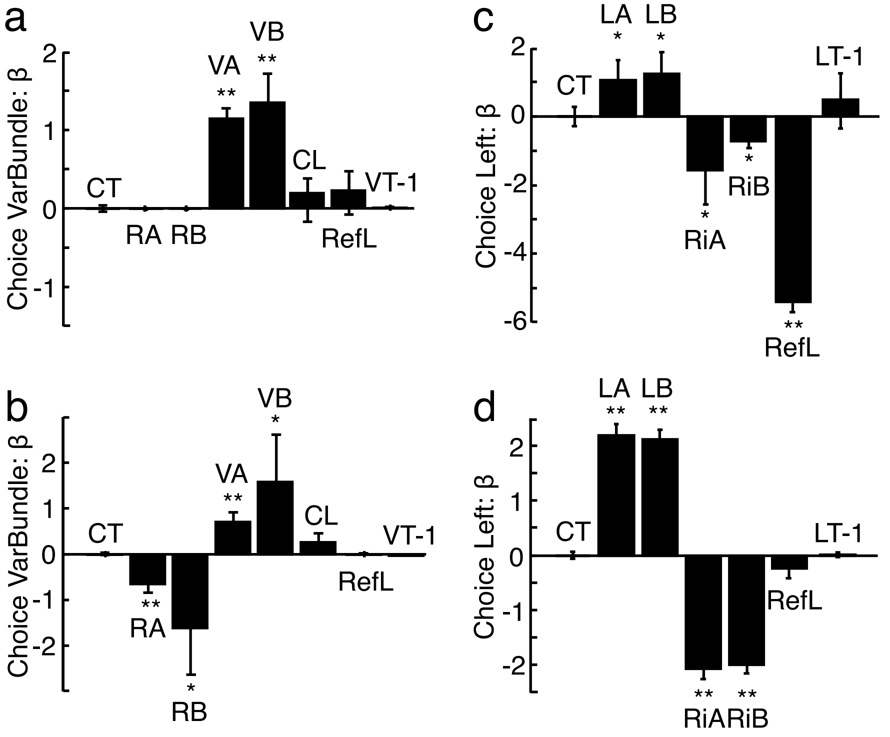

Supplementary Fig. 3. Behavioral controls. **a** Value control. Logistic regression on choice of Variable Bundle (choice over zero-value Reference Bundle, both rewards zero quantity; Eq. 2; see Methods: Control regressions for behavioral choice). The β (slope) coefficients indicate the strength of correlation with choice of the Variable Bundle (Choice VarBundle: β). Choice of Variable Bundle correlated with quantity of rewards A and B of Variable Bundle (VA and VB) (no influence of zero-rewards of Reference Bundle, RA and RB). Bundle choice varied only insignificantly with consecutive trial number (CT), left-right choice (CL), left-right position of zero-value Reference Bundle stimulus (RefL), and choice of Variable Bundle in previous trial (VT-1). * $P < 0.05$; ** $P < 0.01$; t-test on βs. Thus, choice depended on reward quantity of bundle components, rather than on consecutive trial number, spatial parameters, or previous choice. Total of 90,000 trials, both animals. **b** as **a** but choice of Variable Bundle over non-zero Reference Bundle. The choices followed significantly the quantity of individual bundle rewards (RA, RB, VA, VB) but varied insignificantly with consecutive trial number (CT), left-right choice (CL), left position of Reference Bundle stimulus (RefL), and previous Variable Bundle choice (VT-1). **c** Spatial control. Logistic regression on choice of bundle presented at the left on computer monitor (choice over zero-bundle; Eq. 3). The β coefficients indicate correlation strength with left choice (Choice Left: β). Left choice correlated with quantity of reward components A and B presented at the left (LA and LB) and, inversely, at the right (RiA and RiB). Left choice correlated inversely with left position of the zero-value Reference Bundle stimulus (RefL, i. e. the zero-value Reference Bundle was not chosen). Choice varied only insignificantly with consecutive trial number (CT) and left-right choice in previous trial (LT-1). * $P < 0.05$; ** $P < 0.01$; t-test on βs. Thus, choice depended on reward quantity of bundle component, rather than on consecutive trial number or previous choice. **d** Same as **c** but choice of Variable Bundle over non-zero Reference Bundle. The choices followed significantly the quantity of individual bundle rewards (LA, LB, RiA, RiB) but varied insignificantly with consecutive trial number (CT), left position of Reference Bundle stimulus (RefL), and previous left-right choice (LT-1). Same trial number as for **c**. Total of 125,000 trials, both animals.

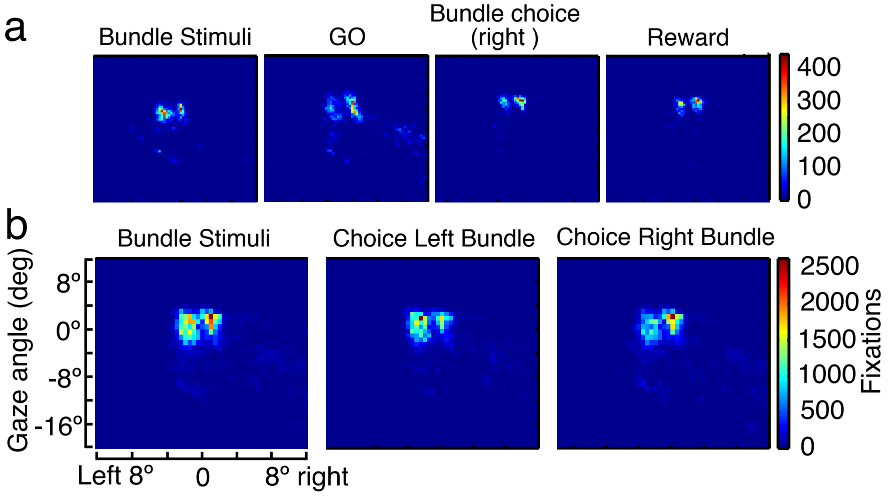

Supplementary Fig. 4. Oculomotor control. **a** Ocular fixation patterns (eye positions) during the four task epochs (one continuous block of 80 trials). **b** Ocular fixation patterns during the most effective task epochs (presentation of two bundle stimuli on computer monitor, bundle choice, one testing day with 585 trials). **c** Satiety control: settings of anchor test bundles: (0.4 ml blackcurrant juice, 0.0 ml grape juice); (0.0 ml blackcurrant juice, 0.1 – 0.6 ml varying grape juice).

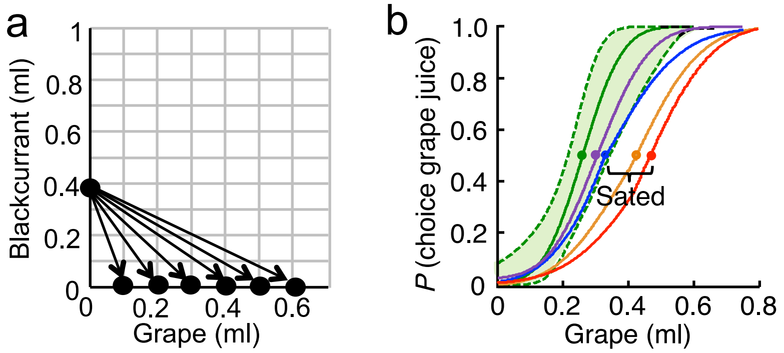

Supplementary Fig. 5. Satiety control. **a** Test scheme and tested quantities of variable grape juice in choice against fixed quantity of blackcurrant juice. **b** Psychophysical assessment of IPs (colored dots) during choice between varying grape juice quantities and constant blackcurrant juice quantity (80 trials per choice function). Green zone with green and maroon curves: absence of significant currency change between the two juices (dotted green lines: ± 95% confidence interval, CI, of choice function). Neuronal recordings were only conducted while IPs were within the green zone but abandoned after IPs or choice functions went outside the green CI, indicating significant currency change (blue, orange, red) that would reflect relative satiety for one juice (grape juice) vs. the alternative juice (blackcurrant).

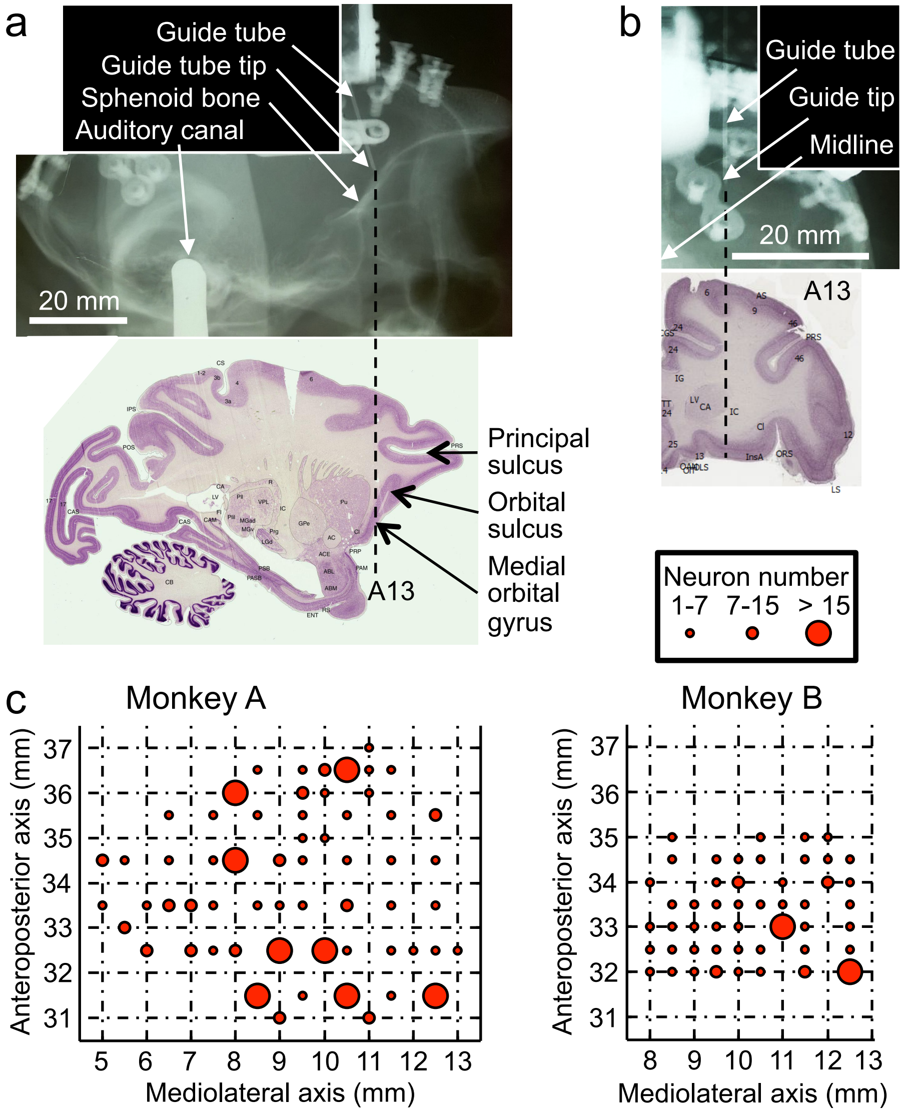

Supplementary Fig. 6. Localisation of recording sites in orbitofrontal cortex. **a** Top: Lateral radiograph of monkey skull. White arrows indicate guide tube (containing microelectrode during recording session) and skull landmarks (sphenoid bone, auditory canal indicated by metal earbar). Bottom: Cresyl violet stained histological parasagittal section of monkey brain. Black arrows indicate anatomical landmarks. Dotted vertical line indicates A13 level according to brain atlas, as approximate position of tip of a guide cannula inserted at 20° forward-directed angle[3]. **b** Coronal section. **c** Surface view of recording locations in Monkeys A and B.

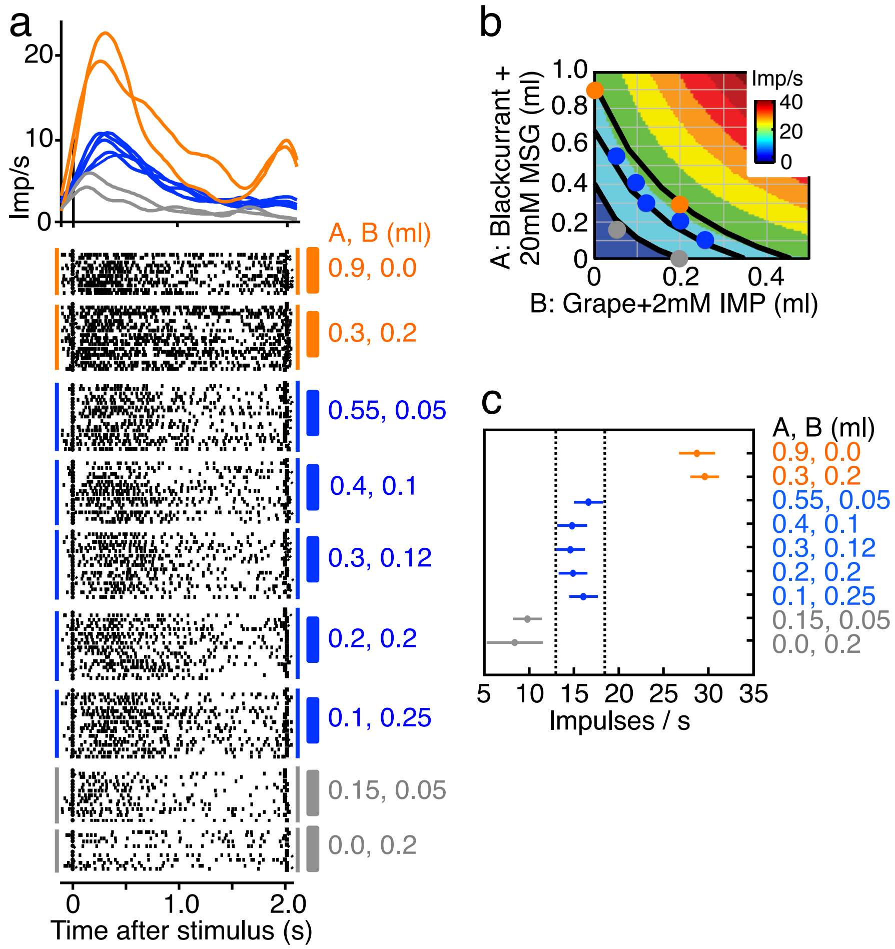

Supplementary Fig. 7. Complete revealed preference test on convex indifference curve (IC) in single orbitofrontal neuron shown in Fig. 2a-c. **a** Same response along same IC representing same revealed preference (blue), and response increase to bundle stimuli across higher ICs (gray to blue to orange; colors refer to dots in **b**; choice over zero-bundle). A, B (ml) refers to quantity of reward A (y-axis in **b**) and reward B (x-axis in **b**) in chosen non-zero bundle (choice over zero-bundle). Neuronal impulses are shown as post-stimulus time histograms above neuronal rasters. Tested bundles are indicated in **b** and were tested in pseudorandom alternation; histograms and rasters were post-hoc ordered. **b** ICs with color dots marking locations of bundles tested in **a**. Black curves show behavioral ICs. Color bands show neuronal ICs estimated from stimulus responses for bundles whose components are specified by their x-y coordinates. **c** Response means and confidence intervals for neuronal responses shown in **a**, demonstrating similar, insignificantly varying response along same-revealed-preference ICs (orange, blue, grey, respectively) ($P > 0.05$; within-IC factor in two-factor Anova) and significant response increase across increasing ICs (from grey to orange) ($P = 1.96e\text{-}102$; $F = 70.78$; across-IC factor in two-factor Anova).

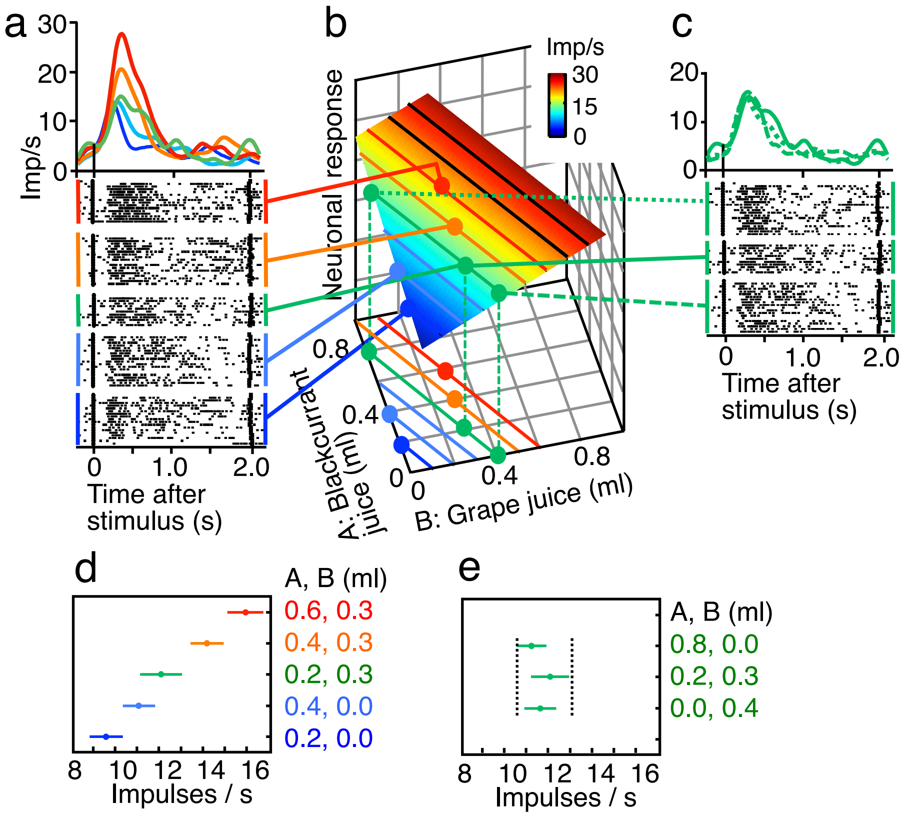

Supplementary Fig. 8. Revealed preference coding for linear indifference curve (IC) in single orbitofrontal neuron. **a** Increasing neuronal responses to stimuli of revealed preferred bundles (from blue via green to red). Choice over zero-reward bundle. Bundle locations on IC are indicated as dots in **b**. Responses increased with quantity of blackcurrant juice (dark blue to light blue, and green via orange to red) and with quantity of grape juice (dark blue to green, and light blue to orange). **b** Bundle positions (colored dots) on hyperbolically fitted behavioral ICs (green, red, blue) tested in **a** and **c**, colored bands show fitted response strength of this neuron using regression with interaction (Eq. 7; see Methods: Statistical analysis of neuronal revealed preference coding) (imp/s: impulses/second). Vertical dotted lines connect the 3D map to its 2D projection at bottom. **c** Similar, insignificantly varying responses to bundles on equal-preference IC despite bundle composition variation (green dots along green IC in **b**; same reward sensitive neuron as shown in **a**, **b**), reflecting equal-preference trade-off between differently composed bundles. Similar responses in top left bundle (blackcurrant juice 0.8 ml, grape juice 0 ml) (highest green dot) and center bundle on same IC (blackcurrant and grape juice each 0.3 ml) (center green dot), suggesting that the gain of 0.3 ml grape juice substituted in neuronal response for the loss of 0.5 ml blackcurrant juice (analogous to Fig. 1e, f). **d** Means and confidence intervals (CIs) of significant response increase across different ICs for the tests shown in **a** (P = 1.53e-25; F = 33.5; across-IC factor in two-factor Anova; 1.0 s analysis window after bundle stimulus onset). Similar CIs in all five responses indicate similar variability of neuronal responses (20 trials with each bundle). A, B (ml) refers to quantity of blackcurrant juice and grape juice, respectively, in chosen non-zero bundle, as plotted at respective y- and x- positions in color map of **b**. **e** Means and CIs of similar, insignificantly varying responses along equal-preference IC despite bundle composition variation (from tests shown in **c**; P = 0.13; F = 2.05; within-IC factor in two-factor Anova).

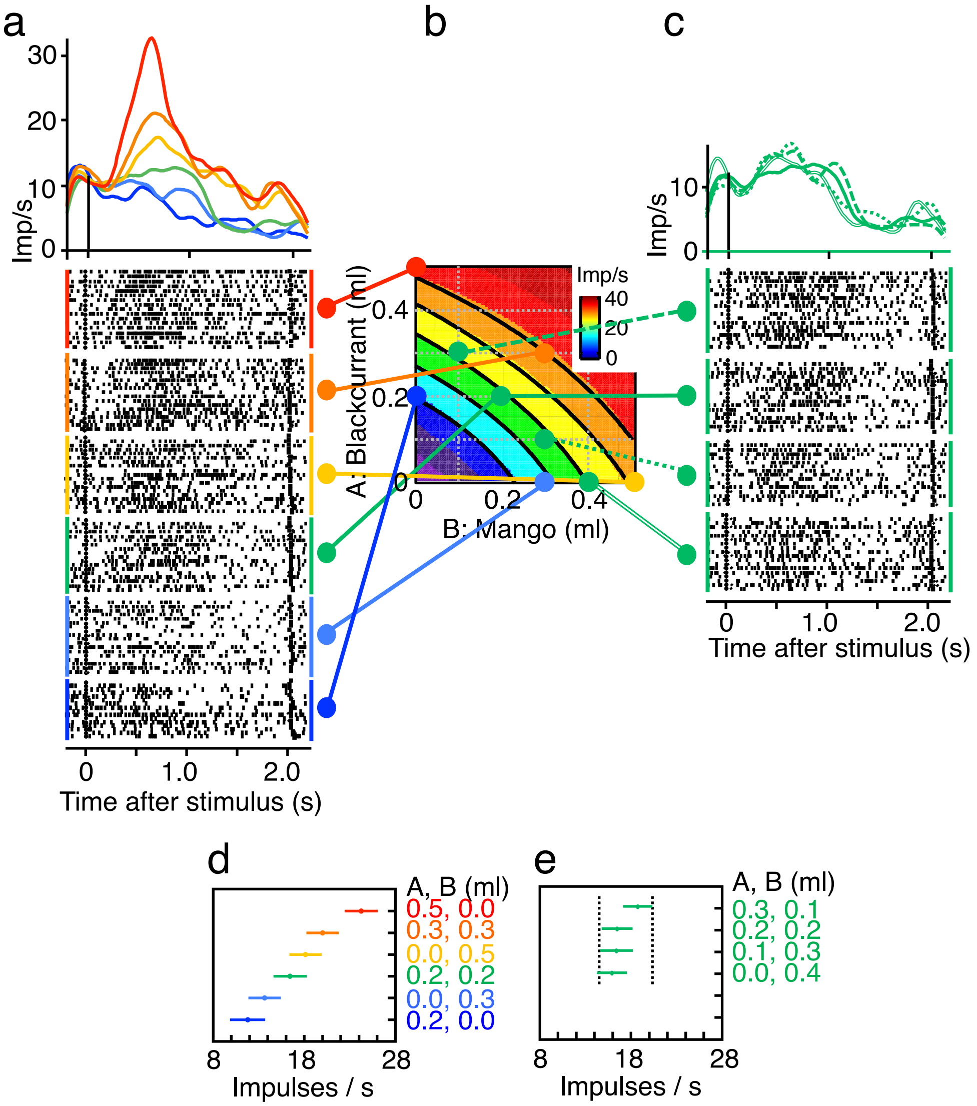

Supplementary Fig. 9. Revealed preference coding for concave indifference curve (IC) in single orbitofrontal neuron. **a-c** Neuronal response increase across increasing concave ICs (**f**) and similar responses reflecting trade-off along same-preference IC (**c**) (choice over zero-bundle). Same conventions as for Fig. 7. **d** Response means and confidence intervals for significant neuronal response increase to bundle stimuli across ICs shown in **a** (black to blue to red; P = 2.1e-12, F = 24.1; across-IC factor in two-factor Anova). A, B (ml) refers to quantity of blackcurrant juice and mango juice, respectively, in the chosen non-zero bundle, as plotted at respective y- and x- positions in the color map of **b**. **e** Response means and confidence intervals for insignificantly differing neuronal response to bundles on same IC shown in **c** (same colors; P = 0.11; F = 1.99; within-IC factor in two-factor Anova).

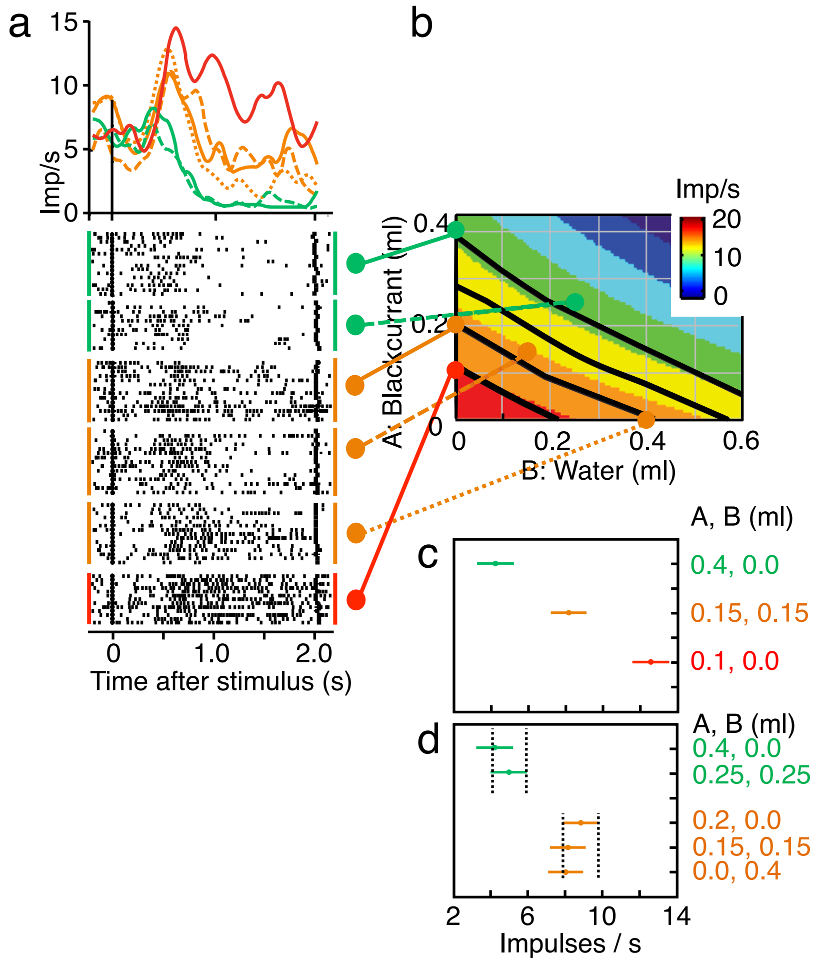

Supplementary Fig. 10. Inverse revealed preference coding in single orbitofrontal neuron. **a** Decreasing neuronal responses to bundle stimuli across increasing indifference curves (ICs) (from red via orange to green), and similar responses between different bundles reflecting trade-off on same-revealed-preference IC (green and orange solid and dotted lines). These responses were recorded in choices over zero-bundle and reflect the revealed preference for the non-zero bundle. **b** Color dots marking locations of bundles tested in **a**. Black curves show behavioral ICs. Color bands show neuronal ICs estimated from similar stimulus responses to bundles whose components are indicated at x-y positions. **c** Response means and confidence intervals demonstrating significant response decreases across increasing ICs (P=6.38e-32; F= 37.3; across-IC factor in two-factor Anova) (original data shown in **a**). A, B (ml) refers to quantity of blackcurrant juice and water, respectively, in the chosen non-zero bundle, as plotted at respective x-y positions in the color map of **b**. **d** Response means and confidence intervals demonstrating insignificant response changes along two ICs (both green and orange bundles; P> 0.05, within-IC factor in two-factor Anova) (original data shown in **a**).

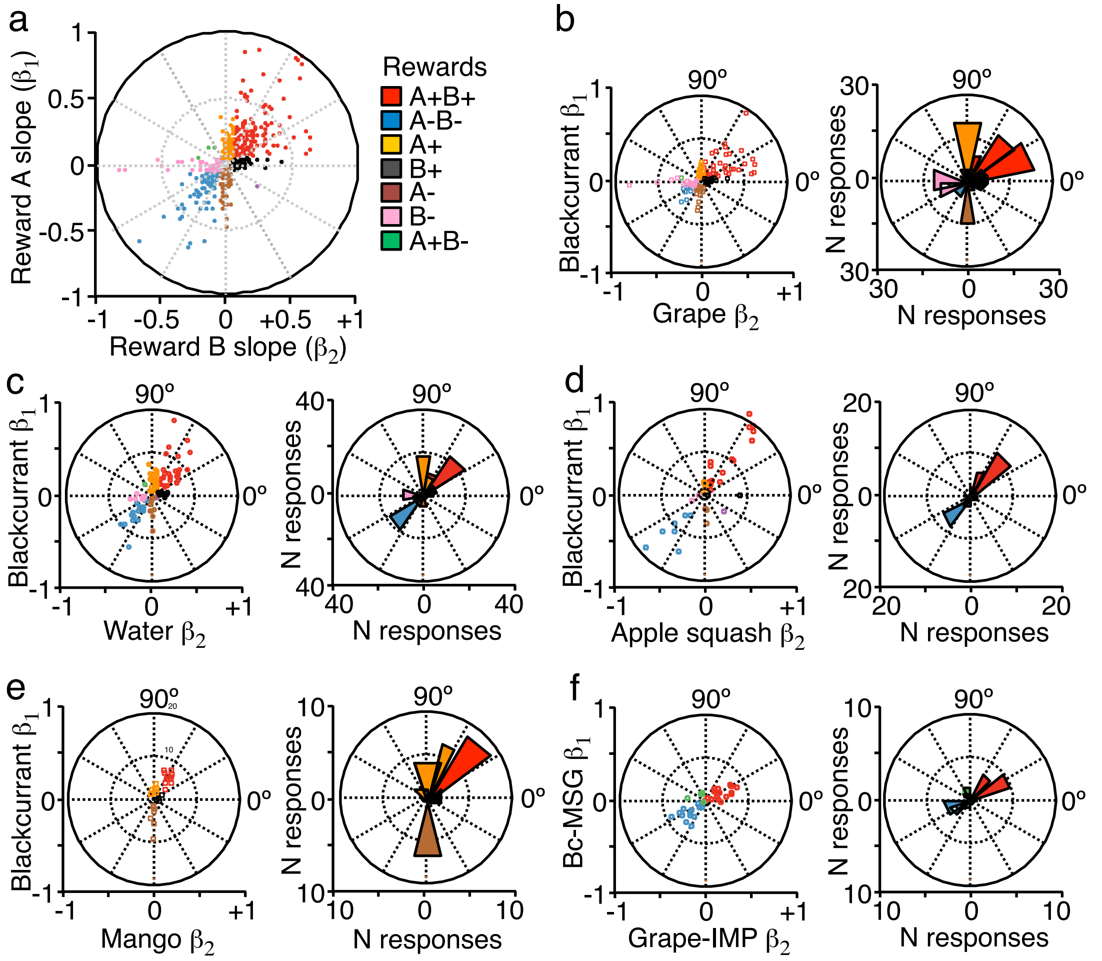

Supplementary Fig. 11. Polar and vector plots of reward coding. **a** Polar plot: coding of one or both bundle rewards. Each dot on the 2D plot shows the two β regression coefficients for neuronal responses (Eq. 4; $P < 0.01$, t-test) for each of the two rewards in any of the four task epochs. The distance from center indicates the z-scored response magnitude (sqrt $(\beta_1^2 + \beta_2^2)$), coding sign (positive or negative) and relative weight (angle; arctangent (β_1 / β_2)) of the two β coefficients. Coefficient β_1 refers to reward A (blackcurrant or blackcurrant+MSG); β_2 refers to any of the other rewards. Dot colors indicate categories based on significance of slope coefficients; A+B+ and A-B-: 187 positive and 76 negative revealed preference responses from 98 and 41 neurons, respectively; A+, B+, A- and B-: 86, 44, 55 and 45 single-reward responses from 51, 30, 36 and 27 neurons, respectively; A+B-: 3 nonclassified reward responses from 3 neurons (Tables 1, 3). The changes for A+B+ and A-B- responses were identified across ICs by multiple linear regression, Spearman rank-correlation and two-factor Anova with significant 1st factor. **b** Polar plot (left) and vector plot (right) of neuronal responses coding revealed preference (A+B+, A-B-), single rewards (A+, B+, A- and B-) and nonclassified (A+B-) for bundle (blackcurrant juice, grape juice). In vector plot, vector length indicates number of significant neuronal responses. **c-f** As **b** but for different bundles as indicated. Common to **a-f**, regression coefficient β_1 refers to neuronal coding of blackcurrant juice; β_2 refers to grape juice (Eq. 4; $P < 0.01$, t-test); choice over zero-bundle; n = number of responses. See Methods: Polar plot of OFC reward sensitivity.

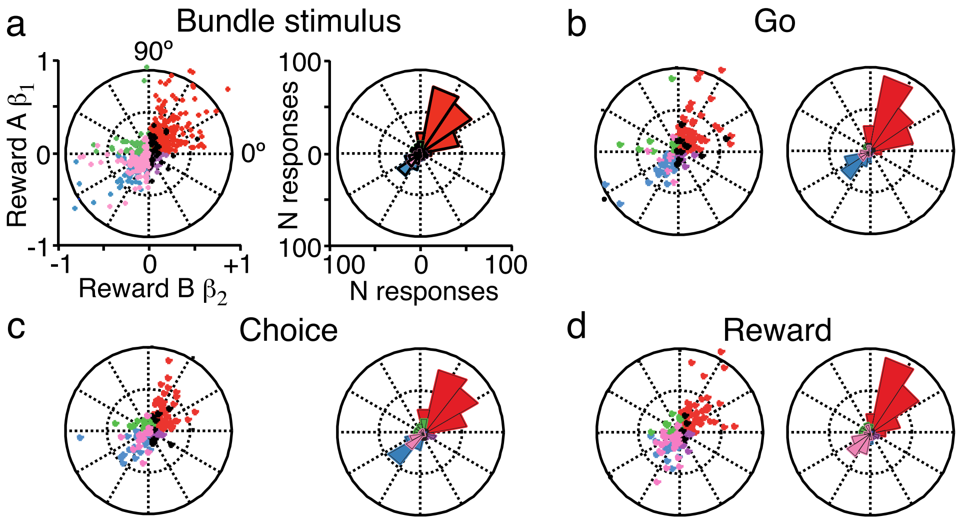

Supplementary Fig. 12. Polar and vector plots of value responses in the four task epochs. Data are from all five bundle types shown in Supplementary Fig. 11. The β regression coefficients were estimated using Eq. 4 ($P < 0.05$, t-test). Scales in **a** apply to **a-d**. For conventions and explanations, see Supplementary Fig. 11.

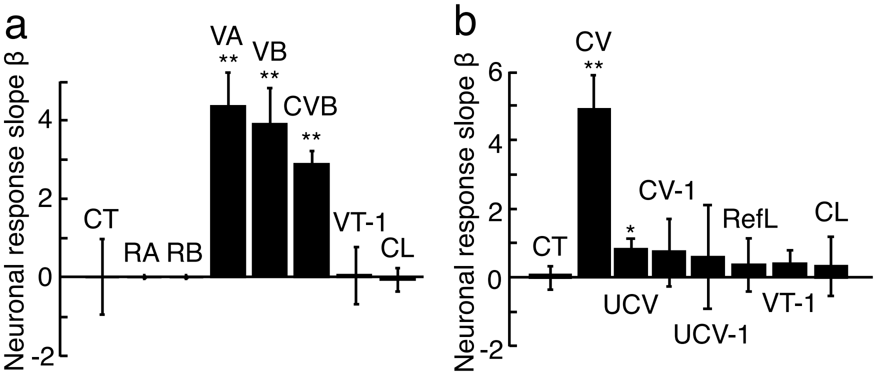

Supplementary Fig. 13. Neuronal relationships to individual bundle components. **a** Choice over zero-bundle (regression Eq. 7). Both rewards of the Reference Bundle were set to zero quantity. The regression slope coefficients β indicate neuronal response strength relative to quantity of reward components in the Reference Bundle (RA and RB) or the Variable Bundle (VA and VB). The responses in the current trial coded significantly the reward quantity of the Variable Bundle (VA and VB) and the choice of the Variable Bundle in the current trial (CVB), but not choice of the Variable Bundle in the previous trial (VT-1). Correlations with the zero-value Reference Bundles (RA and RB) were insignificant. Trial number within block of consecutive trials (CT) and spatial choice (CL) had no significant influence. * $P < 0.05$; ** $P < 0.01$; t-test on βs. Total of 90,000 trials (same as used for Supplementary Fig. 3a, c). Thus, the neurons coded significantly only the reward quantities of the non-zero Variable Bundle. **b** Choice between two non-zero bundles (regression Eq. 10). The responses coded significantly chosen value in the current trial (CV), and to some extent unchosen value (UCV) but not in the previous trial (CV-1 and UCV-1). Consecutive trials (CT), spatial choice (CL), Reference Bundle stimulus position (RefL) and previous choice of Variable Bundle (VT-1) had no significant influence. Chosen value coding was defined from regressions (Eq. 13 together with Eqs. 11 and 12). Total of 125,000 trials (same as used for Supplementary Fig. 3b, d).

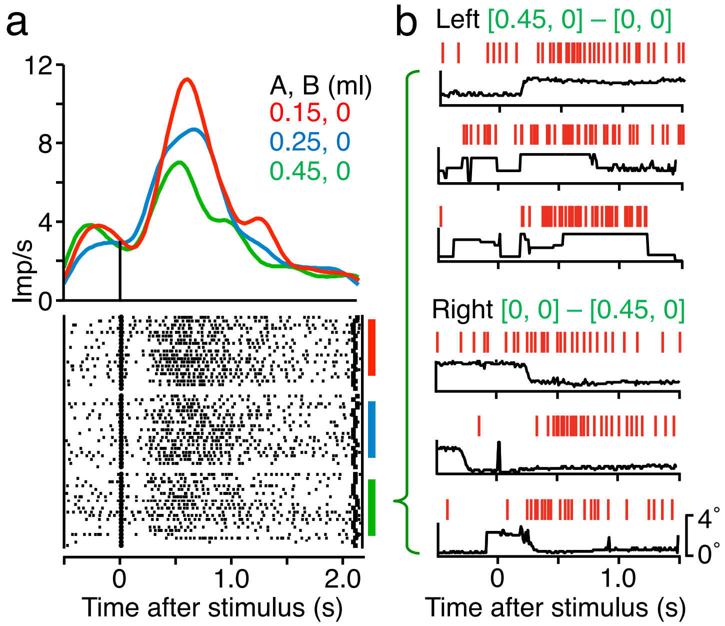

Supplementary Fig. 14. Oculomotor relationship in single reward-sensitive OFC neuron. **a** Bundles response to stimuli for three different bundles: monotonic decrease with increasing quantity of reward A (blackcurrant juice) (inverse coding). Bundle reward B was set to 0 ml in all three bundles. Both rewards in the alternative bundle had been set to 0 ml (choice over zero-reward bundle). Trials are from pseudorandomly intermixed left and right stimulus positions of the same non-zero bundle on the computer monitor, post-hoc separated according to quantity of reward A. **b** Oculomotor relationship: slightly, but significantly stronger neuronal responses with bundle stimulus presentation and behavioral choice at left compared to right (rho = 0.13, $P < 0.001$; Spearman rank-correlation on eye positions from left to right during 2.0 s following bundle stimulus (total angle of 4º); upward deflection indicates left eye movement). The six displayed trials were pseudorandomly selected from the responses to the highest reward, as indicated by green vertical bar in **c**.

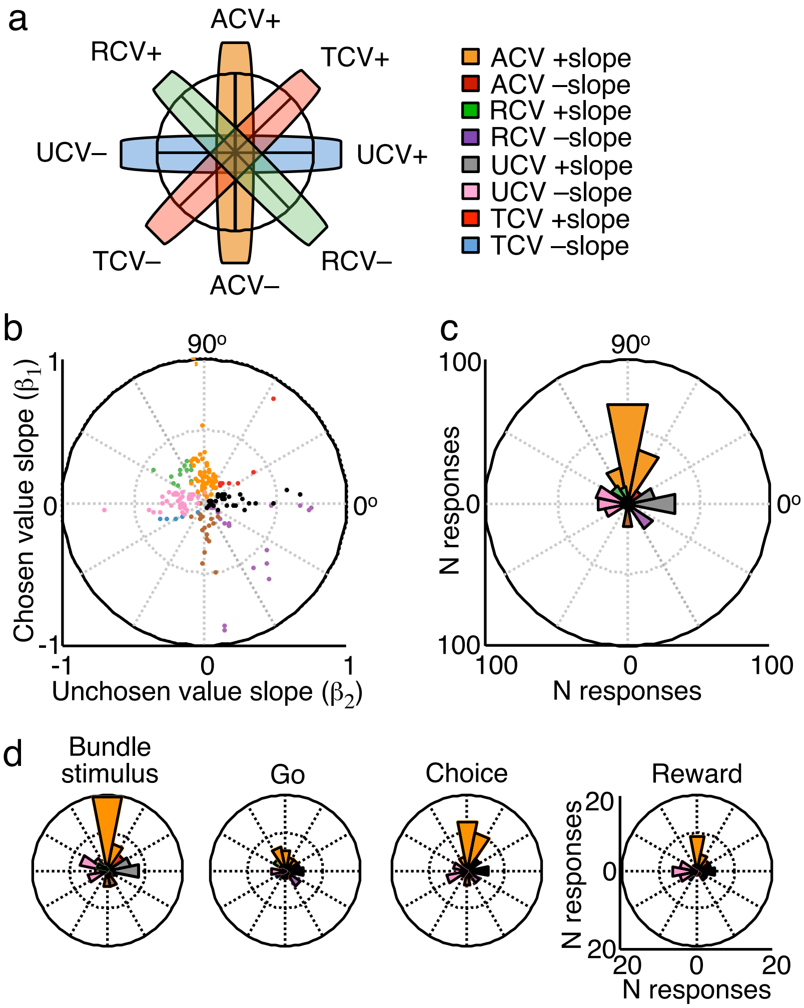

Supplementary Fig. 15. Quantification of chosen value coding. Value coding was estimated with regressions that combine reward quantities linearly. **a** Polar plot scheme of positive and inverse (negative) coding of absolute chosen value (ACV), unchosen value (UCV), relative chosen value (RCV = ACV - UCV) and total chosen value (ACV + UCV). Color code applies to **a-d**. **b** Polar plot of the different forms of chosen value coding schematized in **a**. Each dot shows a neuronal response in any of the four task epochs with any of the bundle types. The position of each dot reflects neuronal coding slopes (distance from center; sqrt $(\beta_1^2 + \beta_2^2)$; β's of z-score normalized responses from Eq. 4), positive or negative sign of the two β regression coefficients (Eq. 4), and relative weight of the two β regression coefficients for chosen and unchosen value (angle; arctangent (β_1 / β_2); β's from Eq. 13 based on Eqs. 11 and 12 that combine reward quantities linearly; $P < 0.05$, t-test). **c** Vector plot indicating numbers of different combinations of chosen value coding, binned for form of chosen value coding indicated in **a**. n = number of responses. **d** Vector plots of chosen value responses in the four task epochs. n = number of responses.

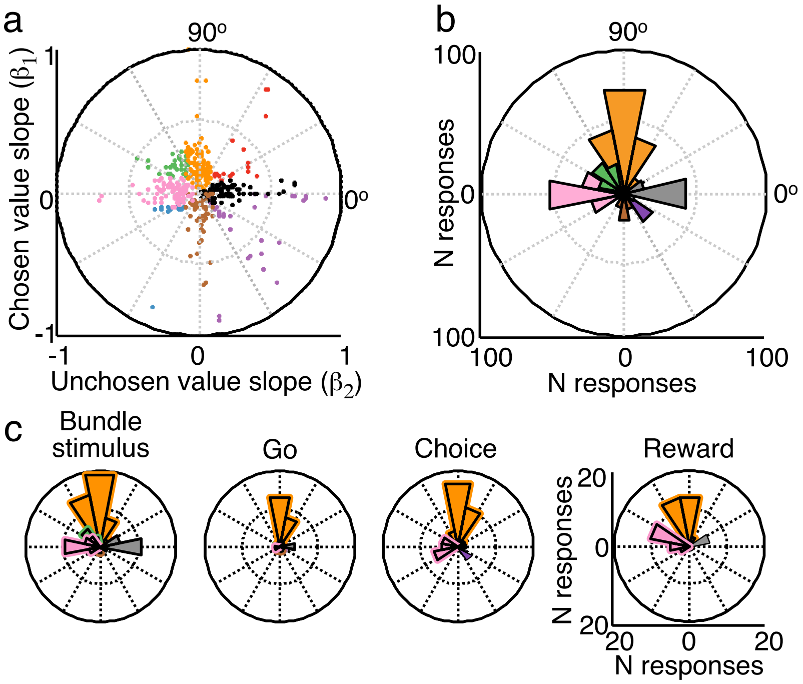

Supplementary Fig. 16. Quantification of chosen value coding estimated by regressions with an interaction term between the two reward quantities that accounts for nonlinear ICs (Eq. 13 based on Eqs. 11a and 12a). All other variables and conventions are as in Supplementary Fig. 15.

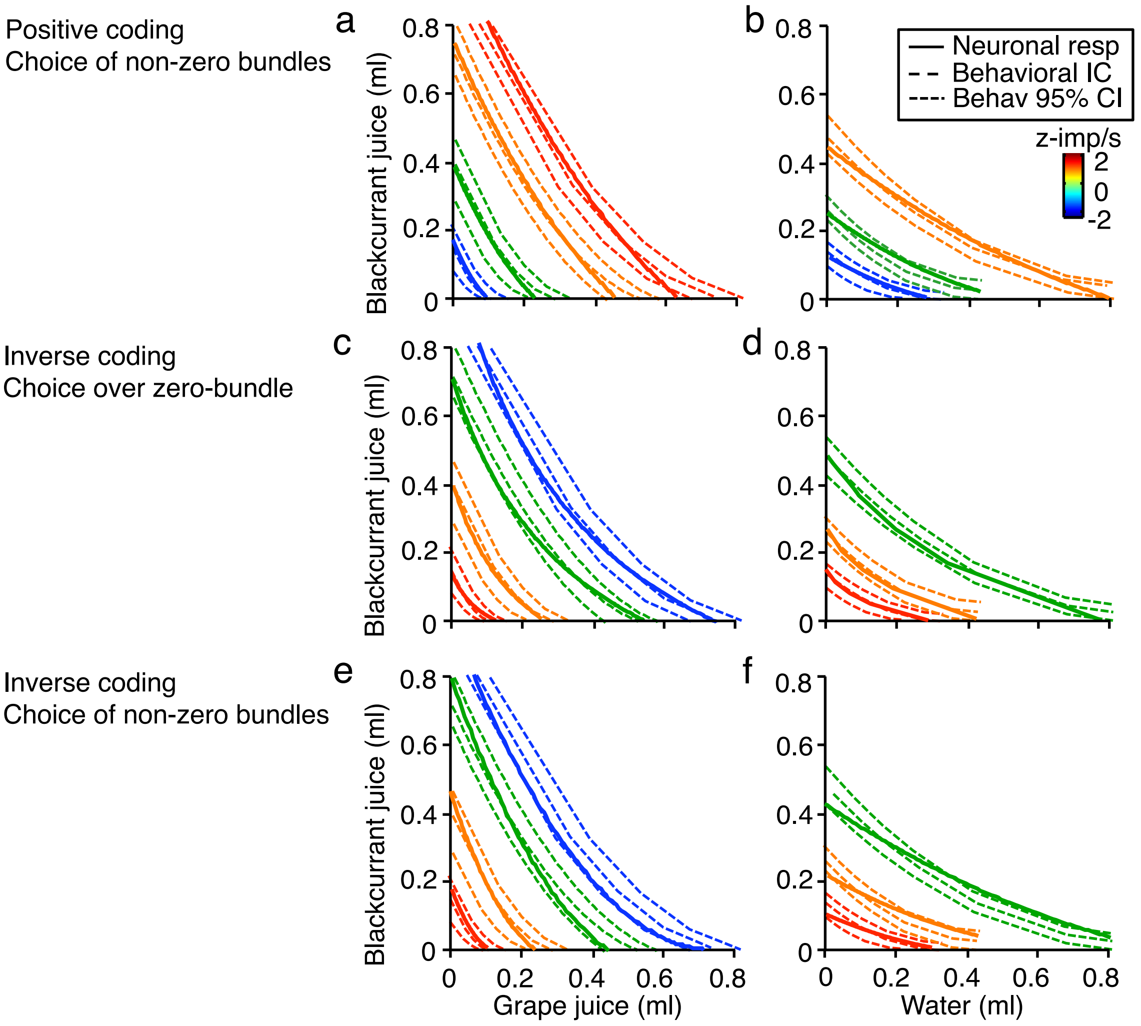

Supplementary Fig. 17. Further neuronal indifference curves (IC) in any task epoch, showing correspondence between neuronal ICs (solid lines) and behavioral ICs (dotted lines, ± 95% confidence interval). **a, b** Neuronal ICs during choice between two non-zero bundles, during any of the four task epochs. Data are from 53 and 45 positively coding responses of 32 and 22 revealed preference neurons tested with bundles (blackcurrant juice, grape juice) and (blackcurrant juice, water), respectively (Table 2; Monkey A only). Neuronal ICs (solid lines) align same neuronal responses at different x-y bundles and were constructed from regression with interaction (Eq. 7, equivalent to behavioral hyperbolic IC fit, Eq. 1); behavioral ICs were plotted from hyperbolic fits (Eq. 1; see Methods: Population plots). Each response derived from multiple trials in one neuron during one task epoch and regressed significantly on the tested bundles (Eq. 7). **c, d** ICs estimated from inversely coding responses, choice over zero-bundle. Data are from 8 and 25 responses of 6 and 14 revealed preference neurons tested with bundles (blackcurrant juice, grape juice) and (blackcurrant juice, water), respectively (27 and 59 different bundles; Monkey Supplementary Table 6; Monkey A). **e, f** As **c**, **d**, but for choice between two non-zero bundles. Data are from 12 and 21 responses of 6 and 10 revealed preference neurons tested with bundles (blackcurrant juice, grape juice) and (blackcurrant juice, water), respectively (Table 2; Monkey A only).

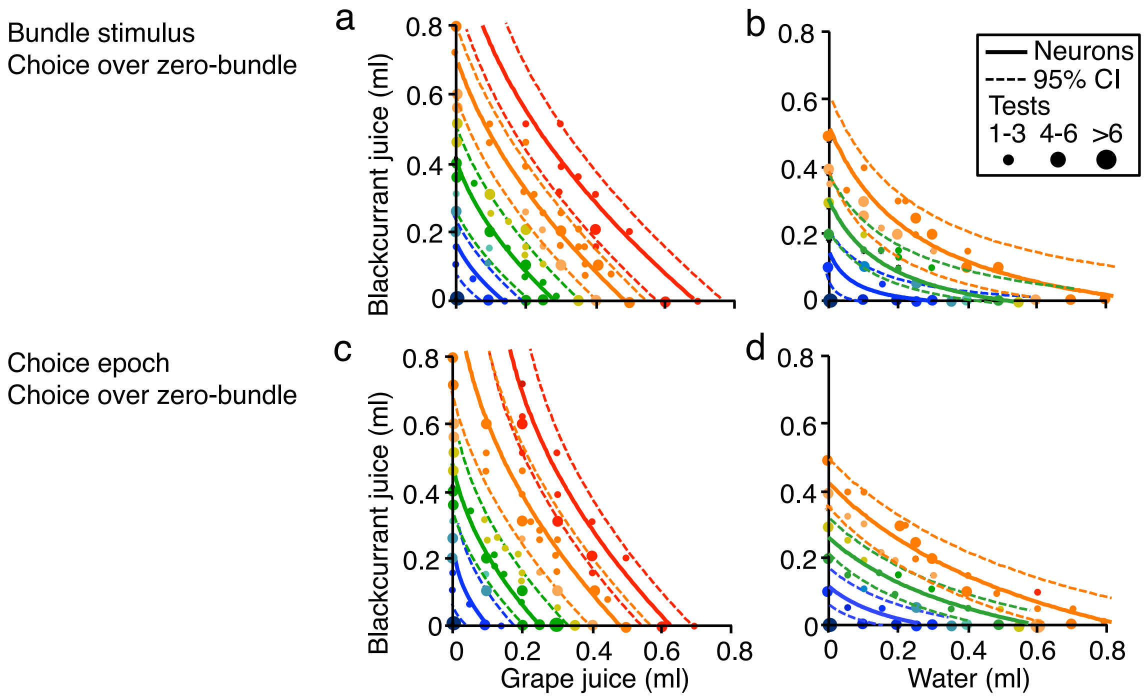

Supplementary Fig. 18. Neuronal indifference curves (IC) from responses during specific task epochs. **a, b** Positively coding neuronal responses to Bundle stimulus in choice over zero-bundle following bundle stimulus. Each dot indicates 1 to > 6 tested bundles (x-y coordinates: liquid quantities; color: response strength). Each neuronal IC was estimated from responses of similar strength (same-color dots). Data in **a** are from 14 responses of 22 revealed preference neurons tested with bundle (blackcurrant juice, grape juice), and in **b** from 13 responses of 23 revealed preference neurons tested with bundle (blackcurrant juice, water). Note that 95% CI refers to neuronal responses. **c, d** As **a, b**, but during Choice epoch. Data in **c** are from 10 responses of 22 revealed preference neurons, and in **d** from 8 responses of 23 revealed preference neurons. Data are from Monkey A, same neuronal response scale (z-imp/s: impulses/second, z-score normalized to pretrial control epoch). For further conventions, see Fig. 3a, b; Supplementary Fig. 17.

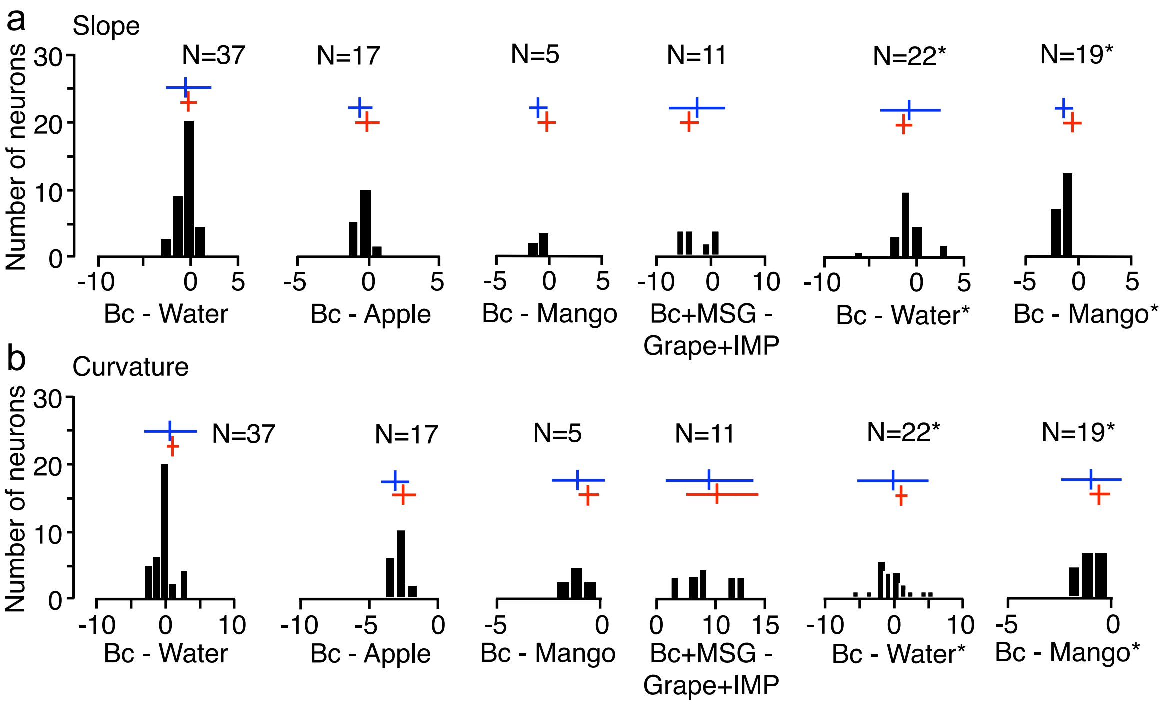

Supplementary Fig. 19. Further comparisons on indifference curve parameters. **a** Similarity between neuronal and behavioral IC slope parameter for several bundle types. Blue bars: neurons (vertical: mean slope, horizontal: ± 95% confidence interval). Red bars: analogous parameters for behavioral ICs. *n* = number of revealed preference neurons with positive or inverse coding responses in any task epoch during choice over zero-bundle, Monkey A (Table 1; Supplementary Table 6), except where indicated by * (Monkey B). This figure extends Fig. 3g. **b** Similarity between neuronal and behavioral IC curvature parameter for same bundle types and neuronal responses as used for **a** (same conventions). This figure extends Fig. 3h.

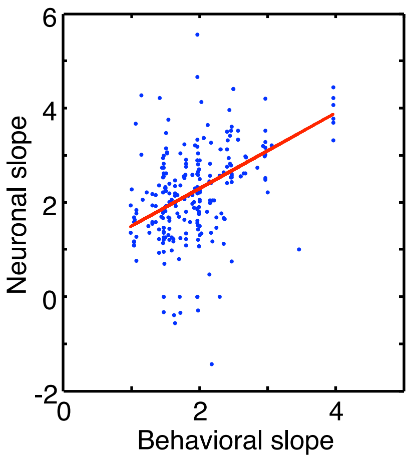

Supplementary Fig. 20. Correlation between neuronal IC slopes and behavioral IC slopes for revealed preference responses for all bundle types combined. Data are from 255 responses in anchor trials from 152 revealed preference neurons during choice between two non-zero bundles.

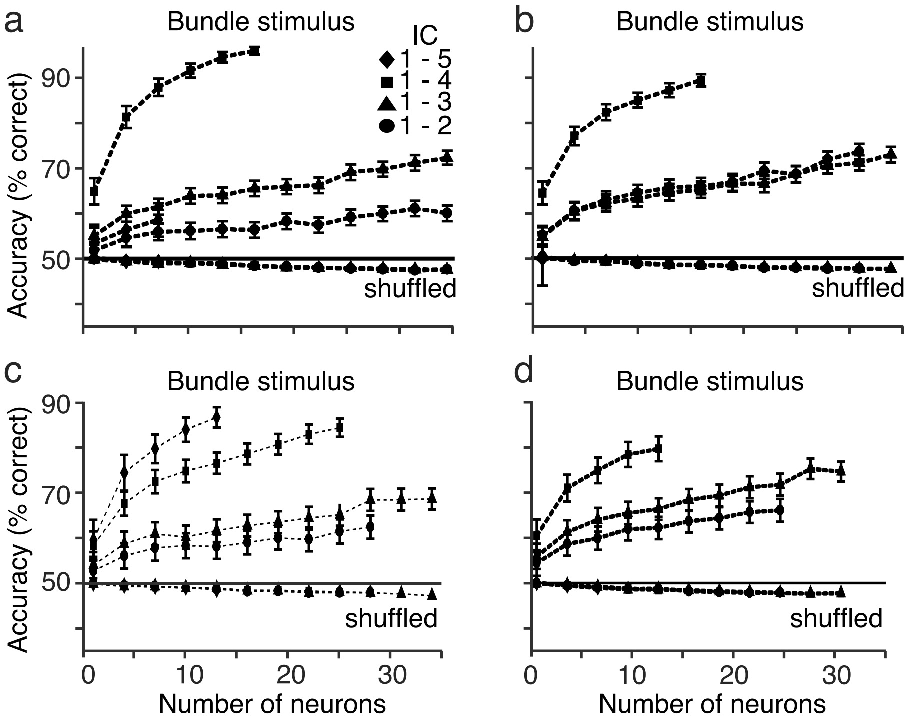

Supplementary Fig. 21. Support vector machine (SVM) decoding during bundle stimulus task epoch. Accuracy increased with neuron number and exceeded chance with shuffled data beyond one neuron ($P < 10^{-20}$, Wilcoxon rank-sum test). **a** Bundle distinction when both components of revealed preferred bundles on indifference curves (IC) 2-4 were larger than in bundles on lower IC 1. Each curve shows decoding of bundles between a higher IC (2-4, farther from origin) and the lowest IC (1, closest to origin; inset) during choice over zero-bundle, based on z-score normalized activity during the Bundle stimulus epoch in neurons showing revealed preference coding during any of the four task epochs, as identified by our three-test statistics ($n = 18$ bundles). **b** As **a** but decoding of bundle choice between two non-zero bundles ($n = 27$ bundles). **c** As **a** but for neuronal activity during Bundle stimulus epoch, and one component of the revealed preferred bundles on ICs 2-5 was larger than, equal to or smaller than in bundles on IC 1 ($n = 56$ bundles). **d** As **c** but decoding of bundle choice between two non-zero bundles. The revealed preferred bundles on ICs 2-5 had one smaller component than the bundles on IC 1, thus dissociating revealed preference rank from physical order ($n = 81$ bundles).

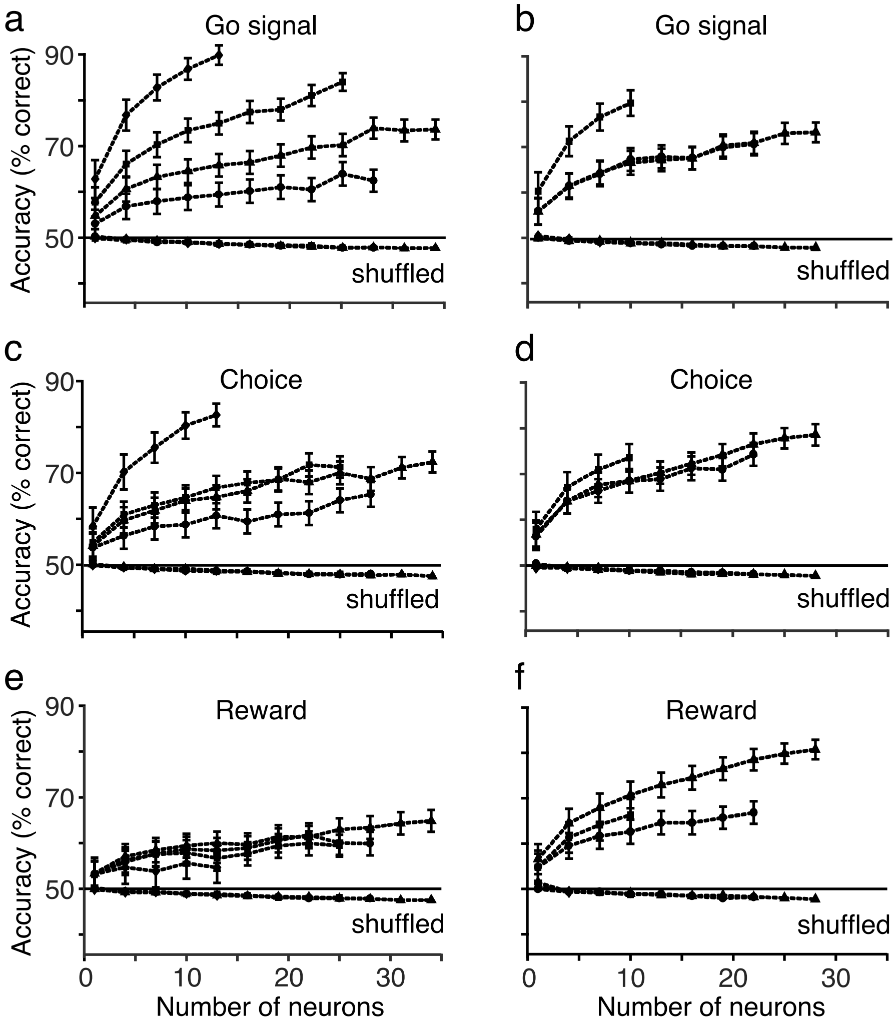

Supplementary Fig. 22. Support vector machine (SVM) decoding during task epochs beyond bundle stimulus. **a** Bundle distinction during Go signal epoch when one component of the revealed preferred bundles on ICs 2-5 was larger than, equal to or smaller than in bundles on IC 1 (choice over zero-bundle). **b** As **a** but decoding of bundle choice between two non-zero bundles. **c** As **a** but for neuronal activity during Choice epoch. **d** As **b** but for neuronal activity during Choice epoch. **e** As **a** but for neuronal activity during Reward epoch. **f** As **b** but for neuronal activity during Reward epoch.

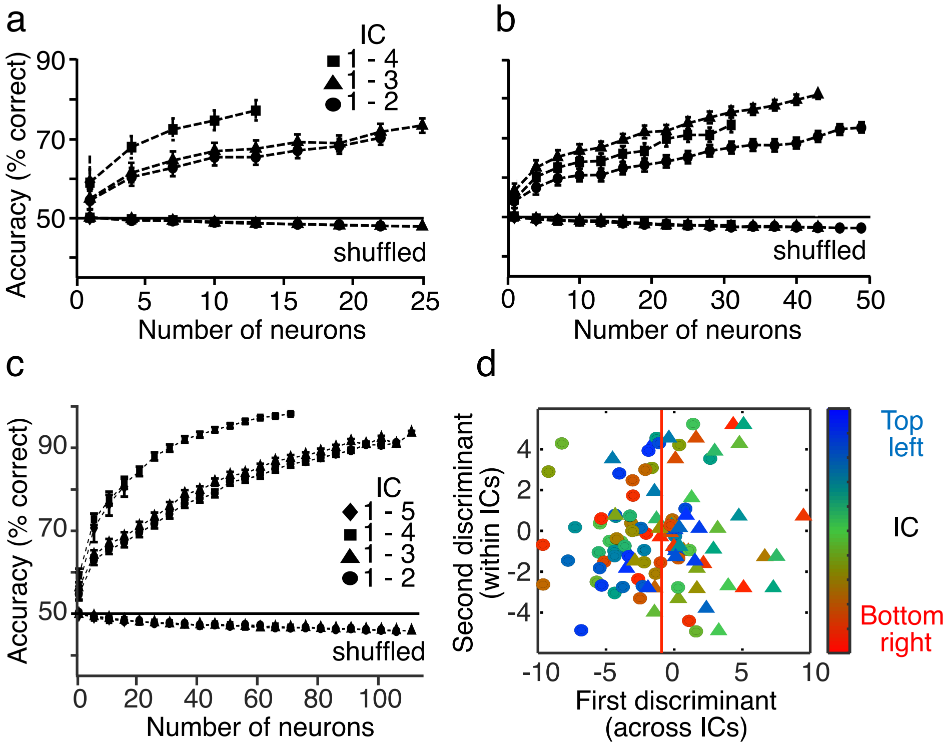

Supplementary Fig. 23. Bundle decoding from unmodulated or unselected neuronal activity. **a** Choice prediction from unmodulated neuronal activity following the bundle stimulus during choice between two non-zero bundles (support vector machine decoder, SVM). The graph includes only data from neuronal activity that coded neither revealed preference nor single rewards. One component of the revealed preferred bundles on indifference curves (IC) 2-4 was smaller than in lower-IC bundles (n = 42 bundles. Decoding accuracy significantly exceeded chance with shuffled data with more than one neuron ($P < 10^{-20}$; Wilcoxon rank-sum test). **b** As **a** but both components of the revealed preferred bundles on ICs 2-4 were larger than in lower-IC bundles (n = 183 bundles). **c** Choice prediction from unselected neuronal activity following the bundle stimulus during choice between two non-zero bundles (SVM decoder). The graph includes all neuronal activity irrespective of any modulation. One component of the revealed preferred bundles on ICs 2-4 was smaller than in lower-IC bundles (n = 252 bundles. Decoding accuracy significantly exceeded chance with shuffled data ($P < 10^{-20}$; Wilcoxon rank-sum test). By contrast, no significant decoding of bundles on same ICs (IC1: $P = 0.0924 \pm 0.0172$, mean ± SEM; IC4: $P = 0.188 \pm 0.031$). **d** Linear discriminant analysis (LDA) visualization of choice prediction from unselected z-scored activity of all task-related neurons following the bundle stimulus during choice between two non-zero bundles located on IC1 and IC4, respectively ($n = 71$ neurons). The revealed preferred bundles on IC4 had one smaller component than the bundles on IC1. The decoder demonstrated numeric significance with the first discriminant (across ICs; $P < 10^{-5}$ against shuffled data; Wilcoxon rank-sum test) but non-significance with the second discriminant (within same ICs; IC1: $P = 0.081 \pm 0.011$, $n = 173$ bundles; IC4: $P = 0.107 \pm 0.024$, $n = 113$ bundles). The plot shows discrimination across ICs (first discriminant, dots vs. triangles, red line separating ICs) but not within ICs (second discriminant, colors indicate bundle position from top left to bottom right of ICs), using superimposed estimated bundle positions from three random selections of 10 trials each of neuronal bundle responses.

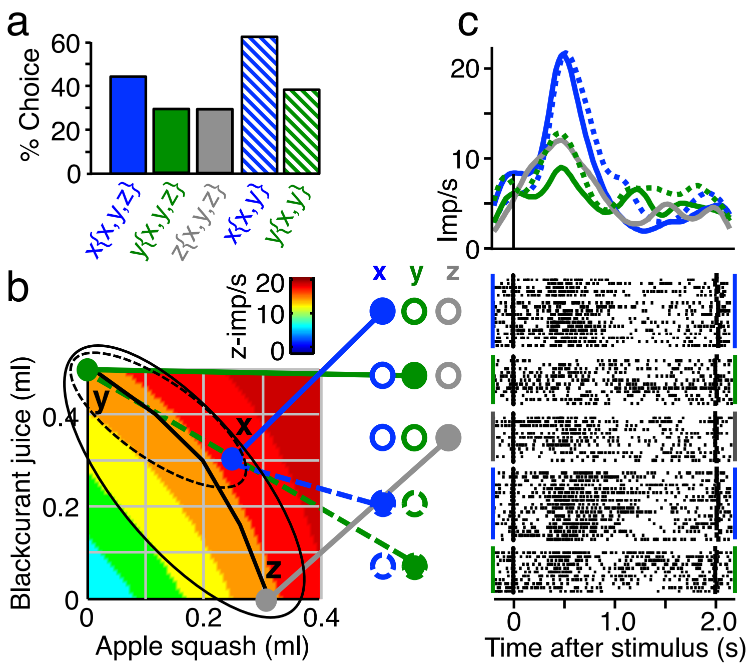

Supplementary Fig. 24. Arrow's Weak Axiom of Revealed Preference (WARP) with concave indifference curve. **a** Behavioral compliance: bundle x remained revealed preferred when restricting the 3-option set **{x,y,z}** to 2 options **{x,y}**. *n* = 70 choices between two non-zero bundles of (blackcurrant, apple squash) while recording from the neuron shown in **b**, **c**. For conventions, see Fig. 5a. **b** Test bundles: colored heat map of neuronal stimulus responses to bundles located on behavioral indifference curve (**y**, **z**; black) and above (**x**). Note that bundle **x** (blue) elicited higher response compared to bundles y and z (green, grey) despite containing one smaller reward; the responses followed the animal's revealed preference irrespective of the underlying bundle composition (similar to Figs. 2a; 5b) and irrespective of full set **{x,y,z}** or restricted set **{x,y}**. Filled dots at right indicate chosen bundles. **c** Neuronal correlate: chosen value responses to revealed preferred bundle x remained highest in the 3-option set **{x,y,z}** (solid blue) as in the 2-option set **{x,y}** (dotted blue), as compared to bundles **y** and **z**. Same neuron as shown in **b**. All choices were between non-zero bundles.

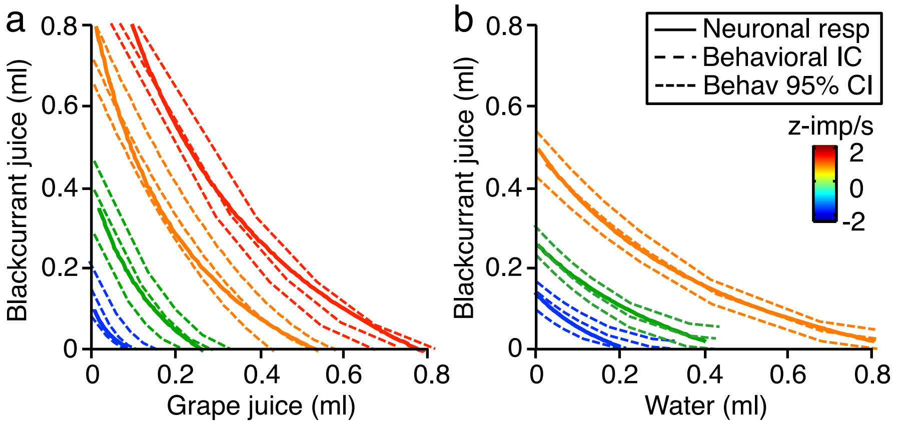

Supplementary Fig. 25. Revealed preference population signal from heterogeneous signals. **a** Correspondence between neuronal ICs (solid lines) and behavioral ICs (dotted lines) for bundle (blackcurrant juice, grape juice). Neuronal ICs were constructed from combining revealed preference and single-reward, positive and negative monotonically coding, z-score normalized responses during choice over zero-bundle in Monkey A (113 responses from 71 neurons). Neuronal responses had been identified by our three-test statistics; neuronal ICs were estimated from regression Eq. 7; behavioral ICs were estimated from equivalent hyperbolic fit (see Methods Population plots point 5d). For conventions, see Fig. 3a, b. **b** As **a** but for bundle (blackcurrant juice, water). Neuronal ICs were constructed from 124 responses from established parameter k_1 81 neurons.

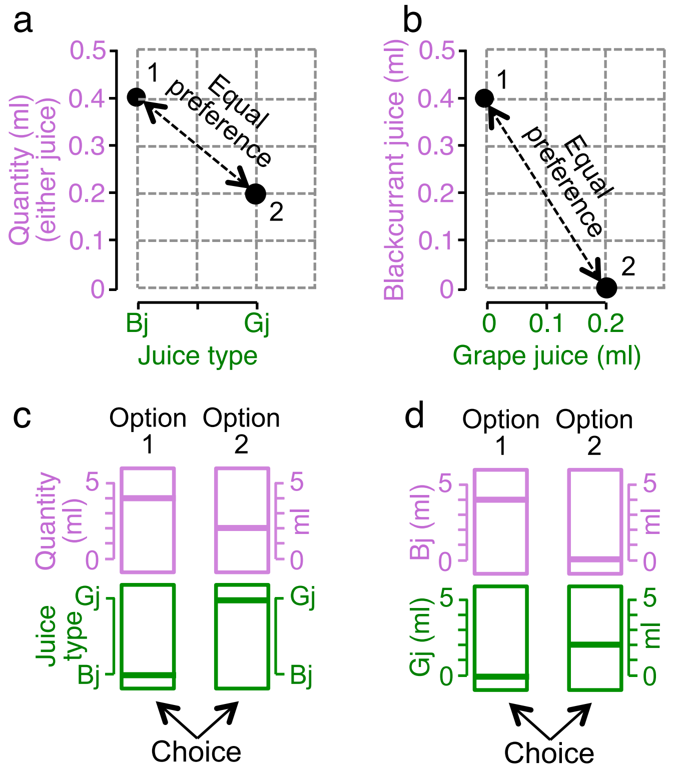

Supplementary Fig. 26. Task design comparisons. **a** Schematic of non-graded trade-off between two equally preferred bundles during psychophysical assessment of choice indifference, as used previously[1,2]. Bundles 1 and 2 contained, respectively, 0.4 ml of blackcurrant juice (Bj) and 0.2 ml of grape juice (Gj). Bundle components were juice quantity (pink) and juice type (green). **b** Same as **a** but plotted according to the quantities of the two juices analogous to Fig. 1a. Bundle components were blackcurrant juice (pink) and grape juice (green). **c**, **d** Hypothetical visual presentation of the two bundles shown in **b** and **c** in the format of Fig. 1c.

Supplementary Table 1. Revealed preference coding in task epochs: choice over zero-bundle.

Bundle type	Numbers of neurons	Bundle stimulus	Go	Choice	Reward	Total
Blackcurrant, grape	22	14+0	9+2	10+3	4+3	37+8=45
Blackcurrant, water*	39	24+15	18+6	18+12	12+8	72+41=113
Blackcurrant, apple	12	8+2	2+3	6+3	4+2	20+10=30
Blackcurrant, mango*	20	17+3	9+2	10+2	6+1	42+8=50
Bc-MSG, grape-IMP	5	7+2	2+3	5+2	2+2	16+9=25
SUM	98	70+22=92	40+16=56	49+22=71	28+16=44	263

In table cells with multiple entries, the first two numbers refer respectively to positive and negative (inverse) relationships to increasing reward quantity, as inferred from the regression slope of neuronal coding (β in Eq. 4). Data are from both animals. For conventions, see Table 1.

Supplementary Table 2. Comparison of RP statistics: choice over zero-bundle.

Model	Linear: Eq. 4	Nonlinear: Interaction Eq. 7	Nonlinear: Quadratic Eq. 8	Nonlinear: Higher-Order Eq. 9	
Bundle type	Adj R2	Adj R2	Adj R2	Adj R2	Neurons
Blackcurrant, grape	0.60	0.75	0.66	0.68	28
Blackcurrant, water*	0.63	0.79	0.77	0.64	59
Blackcurrant, apple	0.65	0.65	0.64	0.67	17
Blackcurrant, mango*	0.55	0.84	0.78	0.65	24
Bc-MSG, grape-IMP	0.58	0.83	0.84	0.62	11
Average	0.60	0.77	0.74	0.65	139
R2 P-value against Eq. 4		0.0025	0.0180	0.0210	139
R2 P-value against Eq. 7			0.0150	0.0160	139
R2 P-value against Eq. 8				0.0210	139

Comparison of variance-explained (adjusted R2) for revealed preference (RP) coding obtained from different regression models, choice over zero-bundle. Neuron numbers refer to the 139 revealed preference coding neurons with significant responses in Eq. 4 ($P < 0.05$), as listed in Table 1. Only these 139 neurons were tested with regressions defined by Eqs. 7, 8 and 9. The bottom three rows show results of comparison of adjusted R2s obtained from Eqs. 7, 8 and 9 to R2s obtained from Eqs. 4, 5 and 6, respectively (Fisher's Test). The terms Linear and Nonlinear refer to the nature of the curvature of the indifference curve. * Data collapsed from Monkeys A and B. For conventions, see Table 1.

Supplementary Table 3. Comparison of RP statistics in task epochs: choice over zero-bundle.

Model	Linear: Eq. 4		Nonlinear: Interaction Eq. 7		Nonlinear: Quadratic Eq. 8		Nonlinear: Higher-Order Eq. 9		Common fits
Task epoch	Adj R2	Neurons	Adj R2	Neurons	Adj R2	Neurons	Adj R2	Neurons	Neurons
Bundle stimulus	0.55	139	0.60	132	0.63	136	0.69	132	62
Choice	0.56	139	0.63	135	0.68	150	0.73	105	97

Comparison of variance-explained (adjusted R2) for revealed preference (RP) coding obtained from different regression models, choice over zero-bundle. Neuron numbers refer only to significant revealed preference coding ($P < 0.05$ for Eqs. 4, 7, 8 or 9, respectively). The Common fits column lists the number of neurons with significant responses in all four regressions ($P < 0.05$). The 139 neurons (second column) are the same revealed preference coding neurons shown in Table 1 and Supplementary Table 2 ($P < 0.05$; Eq. 4). Data are from all bundle types in Monkeys A and B. For conventions, see Supplementary Table 2.

Supplementary Table 4. Comparison of RP statistics: choice between two non-zero bundles.

Model	Linear: Eq. 4	Nonlinear: Interaction Eq. 7	Nonlinear: Quadratic Eq. 8	Nonlinear: Higher-Order Eq. 9	
Bundle type	Adj R2	Adj R2	Adj R2	Adj R2	Neurons
Blackcurrant, grape	0.60	0.80	0.77	0.76	38
Blackcurrant, water*	0.53	0.74	0.70	0.71	50
Blackcurrant, apple	0.60	0.79	0.75	0.74	23
Blackcurrant, mango*	0.56	0.82	0.79	0.71	28
Bc-MSG, grape-IMP	0.58	0.80	0.78	0.78	13
Average	0.57	0.79	0.76	0.74	152
R2 P-value against Eq. 4		0.0027	0.0193	0.0231	152
R2 P-value against Eq. 7			0.0200	0.0174	152
R2 P-value against Eq. 8				0.0042	152

As Supplementary Table 2 but using responses during choice between two non-zero bundles. Neuron numbers refer to the 152 revealed preference (RP) neurons with significant responses in Eq. 4 ($P < 0.05$), as listed in Table 1. Only these 152 neurons were tested with regressions defined by Eqs. 7, 8 and 9. The bottom three rows show results of comparison of R2s obtained from Eqs. 7, 8 and 9 to R2s obtained from Eqs. 4, 5 and 6, respectively (Fisher's Test). * Data collapsed from Monkeys A and B. For conventions, see Table 1 and Supplementary Table 2.

Supplementary Table 5. Comparison of RP statistics in task epochs: choice of non-zero bundles.

Model	Linear: Eq. 4		Nonlinear: Interaction Eq. 7		Nonlinear: Quadratic Eq. 8		Nonlinear: Higher-Order Eq. 9		Common fits
Task epoch	Adj R2	Neurons	Adj R2	Neurons	Adj R2	Neurons	Adj R2	Neurons	Neurons
Bundle stimulus	0.48	152	0.49	160	0.50	152	0.55	137	74
Choice	0.62	152	0.61	130	0.81	261	0.82	282	96

As Supplementary Table 3 but using revealed preference (RP) responses during choice between two non-zero bundles. The 152 neurons (third column) are the same revealed preference coding neurons shown in Table 1 and Supplementary Table 4 ($P < 0.05$; Eq. 4). For conventions, see Supplementary Tables 3 and 4.

Supplementary Table 6. Data used for constructing neuronal ICs from RP neurons.

Bundle type	Neurons	Responses	Bundles tested
Blackcurrant, grape	22+6=28	37+8=45	78+27=105
Blackcurrant, water	23+14=37	36+25=61	82+59=141
Blackcurrant, apple	12+5=17	20+10=30	46+22=68
Blackcurrant, mango	5+0=5	15+0=15	25+0=25
Bc-MSG, grape-IMP	5+6=11	16+9=25	23+33=56
Blackcurrant, water*	16+6=22	36+16=52	90+61=151
Blackcurrant, mango*	15+4=19	27+8=35	52+20=72

Numbers of neurons, responses and tested bundles for constructing neuronal indifference curves ICs from single neurons identified as coding revealed preference (RP) (choice over zero-bundle). In table cells with multiple entries, the first two numbers refer respectively to positive and negative (inverse) relationships to increasing reward quantity, as inferred from the regression slope of neuronal coding (β in Eq. 4). Revealed preference coding was defined by a combination of significance in multiple linear regression ($P < 0.05$; Eq. 4), Spearman rank-correlation ($P < 0.05$) and two-factor Anova ($P < 0.05$ across-IC, $P \geq 0.05$ within-IC). Bundles tested are defined by bundle type and quantity of bundle rewards. Data were obtained from Monkey A (unmarked) and Monkey B (marked by *). The neuronal indifference curves from the first two rows are shown graphically in Fig. 3a-d and Supplementary Figs. 17 and 25. These data were also used for the quantitative neuronal-behavioral comparisons shown in Fig. 3e, f and Supplementary Fig 19 (see Methods: Neuronal population plots).

Supplementary Table 7. SVM accuracy on ICs closest to origin: choice over zero-bundle.

Measured responses	B2	B3	B4	B5
B1	53.4	54.6	55.5	49.9
B2		46.3	51.2	49.5
B3			48.0	52.9
B4				50.5
Shuffled responses				
B1	51.5	48.8	49.5	50.2
B2		51.4	50.1	49.7
B3			51.6	49.8
B4				49.2

Accuracy (%) of support vector machine (SVM) decoding of bundles along same indifference curves (IC), choice over zero-bundle. Mean percent accuracy of classification for pairwise bundle distinction on same indifference curve (e. g. bundle B1 vs. bundle B2), using z-scored, neuronal responses following bundle stimulus onset. Only neurons with revealed preference response to bundle stimulus are included, as detected by our three-test statistics. Decoding within same ICs was non-significant: $P = 0.34 \pm 0.11$; mean ± SEM from 150 iterations of 10 pairwise comparisons between measured and shuffled activity (Wilcoxon rank-sum test between measured and shuffled responses). B1, B2, etc. indicate bundles, numbered from top left to bottom right.

Supplementary Table 8. SVM accuracy on ICs farthest from origin: choice over zero-bundle.

Measured responses	B2	B3	B4	B5	B7	B8	B9	B11	B12
B1	53.9	49.4	52.5	52.4	53.2	50.3	51.0	50.9	60.2
B2		56.8	58.0	57.2	56.4	48.7	55.6	49.2	54.3
B3			48.9	48.7	53.3	55.1	49.0	56.5	66.7
B4				51.9	51.8	56.4	48.0	59.2	68.7
B5					49.3	57.4	48.0	56.9	70.8
B7						59.1	48.5	57.0	71.4
B8							58.6	50.1	55.5
B9								58.2	68.8
B11									56.6
Shuffled responses									
B1	51.7	49.4	49.3	50.8	52.1	51.8	51.8	50.5	49.9
B2		50.9	50.7	49.3	50.3	48.1	49.3	48.7	49.3
B3			50.5	48.4	52.6	52.9	51.8	52.1	47.5
B4				49.0	50.3	50.8	49.5	48.5	49.2
B5					51.6	49.2	52.4	51.7	49.4
B6						50.3	49.1	51.6	51.4
B7							48.1	51.4	50.3
B8								51.3	51.5
B9									49.7

Accuracy (%) of support vector machine (SVM) decoding of bundles along same indifference curves (IC), choice over zero-bundle. Decoding within same IC was non-significant: $P = 0.23 \pm 0.05$; 150 iterations of 45 pairwise comparisons between measured and shuffled activity (Wilcoxon rank-sum test between measured and shuffled responses). B1, B2, etc. indicate bundles. Some bundles are missing due to insuffficent trial numbers of neuronal recording. For conventions, see Supplementary Table 7.

Supplementary Table 9. SVM accuracy on ICs closest to origin: choice of non-zero bundles.

Measured responses	B2	B3	B4	B5	B6	B7
B1	86.5	75.1	94.9	75.1	94.6	88.0
B2		53.9	62.4	53.7	59.3	49.9
B3			68.8	46.1	67.0	59.3
B4				69.9	50.2	57.3
B5					67.1	57.2
B6						52.6
Shuffled responses						
B1	49.7	52.6	49.5	51.7	48.4	49.8
B2		50.0	50.7	48.5	49.0	52.5
B3			51.0	51.5	49.3	49.6
B4				49.1	49.7	51.9
B5					51.4	51.5
B6						49.1

Accuracy (%) of support vector machine (SVM) decoding of bundles along same indifference curves (IC), choice between two non-zero bundles. Decoding within same indifference curve (IC) was non-significant: $P = 0.24 \pm 0.06$, mean ± SEM; 150 iterations of 21 pairwise comparisons between measured and shuffled activity (Wilcoxon rank-sum test between measured and shuffled responses). B1, B2, etc. indicate bundles. For conventions, see Supplementary Table 7.

Supplementary Table 10. SVM accuracy on ICs farthest from origin: choice of non-zero bundles.

Measured responses	B2	B3	B4	B5	B6	B7	B8	B9	B10
B1	54.8	51.6	47.6	50.8	51.5	53.9	50.6	53.9	52.8
B2		51.2	52.0	51.4	58.0	47.7	54.9	58.1	60.3
B3			54.2	49.2	52.2	46.0	52.0	60.9	56.7
B4				53.3	58.2	54.0	49.1	48.7	58.5
B5					49.6	51.7	47.5	55.4	54.3
B6						55.8	50.0	58.6	48.0
B7							53.1	62.7	59.8
B8								48.7	52.4
B9									59.1
Shuffled responses									
B1	50.6	53.6	49.4	50.1	51.0	52.2	51.2	49.7	50.0
B2		51.3	49.8	49.9	49.4	49.4	50.8	51.3	47.1
B3			48.8	52.2	48.4	53.1	50.9	48.8	52.1
B4				50.2	50.6	51.1	50.1	50.7	50.0
B5					50.8	52.6	49.6	47.9	47.3
B6						47.5	48.1	51.7	47.4
B7							50.9	51.3	51.8
B8								49.8	51.0
B9									52.1

Accuracy (%) of support vector machine (SVM) decoding of bundles along same indifference curve (IC), choice between two non-zero bundles. Mean percent accuracy of classification for pairwise bundle prediction on same indifference curve. Decoding within same ICs was non-significant: $P = 0.26 \pm 0.04$; 150 iterations of 45 pairwise comparisons between measured and shuffled activity (Wilcoxon rank-sum test between measured and shuffled responses). B1, B2, etc. indicate bundles. For conventions, see Supplementary Table 7.

Supplementary Table 11. LDA accuracy on ICs closest to origin: choice over zero-bundle.

Measured responses	B2	B3	B4	B5
B1	48.4	49.6	49.2	38.6
B2		38.6	47.8	40.8
B3			46.2	34.5
B4				50.2
Shuffled responses				
B1	42.9	37.5	42.1	40.2
B2		39.7	42.9	43.3
B3			36.6	34.6
B4				41.1

Accuracy (%) of linear discriminant analysis (LDA) of bundles along same indifference curves (IC), choice over zero-bundle. Decoding within same ICs was non-significant: $P = 0.232 \pm 0.107$, mean ± SEM; 150 iterations of 10 pairwise comparisons between measured and shuffled activity (Wilcoxon rank-sum test between measured and shuffled responses). B1, B2, etc. indicate bundles. Same conventions as Supplementary Table 7.

Supplementary Table 12. LDA accuracy on ICs farthest from origin: choice over zero-bundle.

Measured responses	B2	B3	B4	B5	B6	B7	B8	B9	B10
B1	46.0	38.1	47.2	52.8	57.0	42.9	50.0	46.6	53.2
B2		47.7	51.4	52.7	54.0	42.0	48.3	41.1	47.6
B3			39.6	52.2	52.1	43.2	48.6	53.2	59.8
B4				48.8	43.2	47.2	44.6	52.7	63.0
B5					35.8	56.3	39.7	51.0	68.8
B6						56.9	41.2	54.7	70.1
B7							55.0	42.5	49.5
B8								48.0	64.9
B9									50.7
Shuffled responses									
B1	39.6	43.9	40.4	41.4	42.9	42.1	39.1	39.0	39.0
B2		40.7	40.7	42.3	40.9	40.8	43.2	40.2	38.4
B3			43.6	39.1	42.6	38.8	41.0	36.6	39.8
B4				40.0	39.5	41.5	41.7	41.1	41.2
B5					39.2	41.7	41.7	38.4	41.7
B6						41.1	42.6	40.9	37.6
B7							41.1	44.9	43.9
B8								40.2	38.6
B9									42.8

Accuracy (%) of linear discriminant analysis (LDA) of bundles along same indifference curves (IC), choice over zero-bundle. Decoding within same ICs was non-significant: $P = 0.089 \pm 0.029$; 150 iterations of 45 pairwise comparisons between measured and shuffled activity (Wilcoxon rank-sum test between measured and shuffled responses). B1, B2, etc. indicate bundles. For conventions, see Supplementary Table 7.

Supplementary Table 13. LDA accuracy on ICs closest to origin: choice of non-zero bundles.

Measured responses	B2	B3	B4	B5	B6	B7
B1	84.4	76.0	92.2	79.4	92.4	86.7
B2		47.2	54.7	47.3	50.2	40.1
B3			64.7	39.3	63.3	50.3
B4				63.8	45.4	49.8
B5					62.7	51.6
B6						49.7
Shuffled responses						
B1	40.2	37.7	40.4	44.8	34.6	38.8
B2		40.8	39.6	41.4	40.4	41.9
B3			43.2	43.8	41.0	40.2
B4				41.0	43.3	42.3
B5					44.3	42.7
B6						41.1

Accuracy (%) of linear discriminant analysis (LDA) of bundles along same indifference curves (IC), choice between two non-zero bundles. Decoding within same ICs was non-significant: $P = 0.162 \pm 0.045$, mean ± SEM; 150 iterations of 21 pairwise comparisons between measured and shuffled activity (Wilcoxon rank-sum test between measured and shuffled responses). B1, B2, etc. indicate bundles. For conventions, see Supplementary Table 7.

Supplementary Table 14. LDA accuracy on ICs farthest from origin: choice of non-zero bundles.

Measured responses	B2	B3	B4	B5	B6	B7	B8	B9	B10
B1	59.9	44.5	52.7	38.6	42.9	47.6	37.6	43.6	50.5
B2		45.2	46.9	54.5	69.4	20.6	55.8	59.0	71.6
B3			46.1	37.5	54.0	26.6	44.3	52.2	56.2
B4				47.8	62.0	44.0	45.4	43.0	62.8
B5					51.4	39.0	38.8	48.2	54.3
B6						52.4	46.6	59.8	38.1
B7							43.5	54.8	58.1
B8								46.7	45.4
B9									58.2
Shuffled responses									
B1	38.3	43.1	42.0	40.1	41.1	34.4	41.3	42.3	38.5
B2		40.1	40.4	43.6	42.4	35.6	40.8	34.9	39.1
B3			40.5	41.3	41.7	38.0	41.7	42.6	41.3
B4				40.9	40.1	36.6	35.8	39.5	41.9
B5					42.9	37.0	33.8	40.2	38.3
B6						42.7	41.2	42.4	37.9
B7							38.0	35.2	35.6
B8								39.2	42.3
B9									40.8

Accuracy (%) of linear discriminant analysis (LDA) of bundles along same indifference curves (IC), choice between two non-zero bundles. Decoding within same ICs was non-significant: $P = 0.098 \pm 0.031$; 150 iterations of 45 pairwise comparisons between measured and shuffled activity (Wilcoxon rank-sum test between measured and shuffled responses). B1, B2, etc. indicate bundles. For conventions, see Supplementary Table 7.

Supplementary Table 15. Single-reward coding: choice over zero-bundle.

Bundle type	Tested neurons	Neurons responding	Responses
Blackcurrant, grape	81	24+19=43	34+34=68
Blackcurrant, water*	138	35+26=61	64+37=101
Blackcurrant, apple	29	4+4=8	7+5=12
Blackcurrant, mango*	53	13+10=23	29+23=52
Bc-MSG, grape-IMP	24	5+4=9	6+7=13
SUM	325	144 (44%)	246

Tested neurons refers to task-related neurons in both animals, as assessed by significance in the Wilcoxon and one-factor Anova tests ($P < 0.01$) against pretrial control activity. In table cells with multiple entries, the first two numbers refer respectively to positive and negative (inverse) relationships to increasing reward quantity. Coding of single reward A or B was defined by significance in Eqs. 5 and 6, respectively, and by F-test, Eqs. 4 vs. 5 and Eqs. 4 vs. 6, respectively ($P < 0.05$). Each response derived from multiple trials in one neuron in one of the four epochs and regressed significantly on all bundles tested on that neuron. Thus, a given neuron could have distinct responses in more than one task epoch (the four task epochs were Bundle stimulus, Go signal, Choice and Reward); therefore, the number of significant responses typically exceeded the number of significant neurons. A neuron was designated as single-reward neuron if it had a significant response in our three-test statistics in at least one of the four task epochs. * Data collapsed from Monkeys A and B.

Supplementary Table 16. Single-reward coding: choice between two non-zero bundles.

Bundle type	Tested neurons	Neurons responding	Responses
Blackcurrant, grape	89	28+22=50	45+45=90
Blackcurrant, water*	159	60+46=106	120+83=203
Blackcurrant, apple	35	9+3=12	19+7=26
Blackcurrant, mango*	70	25+17=42	54+38=92
Bc-MSG, grape-IMP	29	10+3=13	15+6=21
SUM	391	223 (57%)	432

For conventions, see Supplementary Table 15.

Supplementary Table 17. Chosen value coding in single-reward neurons.

	Neurons	Responses
Absolute chosen value	54+14=68 (30%)	137+27=164 (38%)
Relative chosen value	2+16=18 (8%)	4+39=43 (10%)
Unchosen value	21+18=39 (17%)	62+53=115 (27%)
Total chosen value	0+3=3 (1%)	0+9=9 (2%)
Tested	223	432

For conventions, see Supplementary Table 15.

Supplementary References

1. Padoa-Schioppa, C. & Assad, J. A. Neurons in the orbitofrontal cortex encode economic value. *Nature* **441**, 223-226 (2006).
2. Lak, A., Stauffer, W. R. & Schultz W. Dopamine prediction error responses integrate subjective value from different reward dimensions. *Proc. Natl. Acad. Sci. (USA)* **111**, 2343-2348 (2014).
3. Paxinos, G., Huang, X.-F. & Toga, A. W. *The Rhesus Monkey Brain in Stereotaxic Coordinates.* Academic Press, San Diego, 2000.
